# Supplementary material for: Systematic review and network meta-analysis with individual participant data on cord management at preterm birth (iCOMP): study protocol
Source: BMJ Open. 2020 Mar 29;10(3):e034595. doi: 10.1136/bmjopen-2019-034595 (PMC7170588; doi:10.1136/bmjopen-2019-034595)
Supplement: Supplementary data [file bmjopen-2019-034595supp001.pdf]

iCOMP

iCOMP Trial Master List January 2020

This table shows all trials that we have identified as being eligible for inclusion in iCOMP. All trials that are part of the iCOMP collaboration have been marked in blue.

Table: Eligible randomised trials to date for the pairwise & network meta-analysis with individual participant data on Cord Management at Preterm Birth (iCOMP)

Trial Country (PI)	Publication year	Start year/ completion year	Sample size	Participants	Intervention	Comparator	Primary outcome/s
Argentina [1] (Carroli)	n/a	2016/2020	700	Singletons, 24-30^6 weeks' GA	DCC: at 90 sec	DCC: ~30 sec	Sepsis (proven and very probable)
Australia [2] (Badurdeen)	n/a	2018/2020	120 (not all preterm)	≥32 weeks' GA*, require resuscitation at delivery	DCC: resuscitation prior to cord clamping (for PPV, clamping delayed until at least 60 sec after colour change of pedicap/neostat; for CPAP, clamping occurs at least 2 min after delivery)	ICC (followed by resuscitation)	Average heart rate between 60-120 sec after birth
Australia [3] (McDonnell)	1997	1994/1994	46	26 to 33 weeks' GA	DCC: 30 sec	ICC	Venous haematocrit
Australia [4] (Kamlin)	n/a	2014/2015	27 (not all preterm)	32-42 weeks' GA*	Arm 1: DCC at 90-180 sec<br>Arm 2: DCC 10 sec after crying and breathing established	DCC: <60 sec	Heart rate 90 sec after birth
Australia [5] (Tarnow-Mordi 2009)	n/a (Pilot for Tarnow-Mordi 2017)	2009/2010	100	<32 weeks' GA	Arm 1: Cord milking during resuscitation - cord cut long (3cm from placenta/ introitus)<br>Arm 2: DCC at 30-60 sec. If baby in extremis, immediate clamping.	ICC: within 10 sec	Haemoglobin 6 hours after birth

Seidler AL, *et al. BMJ Open* 2020; 10:e034595. doi: 10.1136/bmjopen-2019-034595

iCOMP

Trial Country (PI)	Publication year	Start year/ completion year	Sample size	Participants	Intervention	Comparator	Primary outcome/s
					Arm 3: DCC at 30-60 sec + milking		
Australia [6] (Tarnow-Mordi 2017)	2017	2010/2017	1634	<30 weeks' GA	DCC: ≥60 sec	ICC within 10 sec	Composite: Death or major morbidity (severe brain injury, severe ROP, NEC, or late onset sepsis) at 36 weeks' PMA
Austria [7] (Urlesberger)	n/a	2018/2021	80 (not all preterm)	>=28 weeks' GA*, caesarean	DCC: <30 sec, cord milking after long clamping at 30cm, 1x 10cm/sec	Standard care (cord cutting, no milking)	Changes in cerebral blood volume within 15 min after birth
Bangladesh [8] (Yasmeen)	2014	2012/2013	40	<37 weeks' GA	DCC: 3 minutes	DCC: 1 minute	Haemoglobin, iron and ferritin
Canada [9] (Chu/Murphy)	n/a	2007/2010	296	Singletons, 24-32 weeks' GA	DCC: at 30-45 sec	ICC	Composite: IVH or late onset sepsis
Canada [10] (El-Naggar)	2018	2011/2014	73	24-30^{6} weeks' GA	UCM: x3, at or below the level of the placenta, ~20 cm milked, before clamping	ICC	Systemic blood flow (Superior vena cava flow at 4-6 hours after birth)
Canada [11] (Saigal)**	1972	n/a	125 (preterm)	Premature infants 28-36 weeks GA and weighing 1020g-3250g OR full-term infants 28-42 weeks GA and weighing 2685g-4350g*	Arm 1: DCC at 1min<br>Arm 2: DCC at 5min	ICC	RBC volume, blood volume, haematocrit, plasma volume
China [12] (Dai)	2014	n/a	52 (preterm)	Singletons, term and preterm infants*	DCC: Wait until cord pulsation ceased	ICC: 5-10 sec	RBC count (72-96 hrs after birth), Anaemia (2 wks), clinically significant pathological polycythaemia, white blood cell count (72-96 hrs after birth), fetal bilirubin from birth to day 5, jaundice within 24 hrs of birth, Apgar

Seidler AL, *et al. BMJ Open* 2020; 10:e034595. doi: 10.1136/bmjopen-2019-034595

iCOMP

Trial Country (PI)	Publication year	Start year/ completion year	Sample size	Participants	Intervention	Comparator	Primary outcome/s
							(1 min, 5 min), respiratory distress, rectal temperature 5 min after birth, neonate well-being at 1 month
China [13] (Dong)	2016	2015/2015	90	Singletons, <32 weeks' GA	DCC: 45 sec	ICC: <10 sec	Severe IVH – grades 3 and 4
China [14] (Hao)	n/a	2018/2019	48	30-31^6 weeks' GA	UCM	DCC	Cerebral haemodynamics 15 min after birth
China [15] (Hu)	2015 (master's thesis)	n/a	120	28-35 weeks' GA, vaginal birth	Arm 1: DCC at 30 sec<br>Arm 2: DCC at 60 sec<br>Arm 3: DCC at 120 sec	ICC < 10 sec	Haematocrit and haemoglobin levels at 24 hrs and 1 wk after birth
China [16] (Hua)	2010?	2009/2011	176 (49 of those preterm)	Any GA*	Normal birth<br>Arm 1: DCC – wait until cord ceases pulsing<br>Arm 2: DCC – at 90 sec<br>Asphyxia<br>Arm 1: DCC – wait until cord ceases pulsing, resuscitate on bed site with cord intact	Normal birth<br>ICC <10 sec<br>Asphyxia<br>ICC <10 sec, resuscitate after on irradiation table	Haemoglobin 1 month after birth
China [17] (Li)	2018	2017/2017	102	delivered vaginally between 28^0-36^6 weeks', and premature prolonged rupture of membranes	UCM: x4 at a speed of 10cm/sec, then clamped	ICC	Incidence of certain or probable infection in neonates
China [18] (Liu)	n/a	2019/2019	948 (not all preterm)	34^0-38^6 weeks' GA*, caesarean section	DCC: 60 sec	ICC: within 10 sec	Rate of respiratory distress within 24 hours after birth
China [19] (Shi)	2017	n/a	60 preterm (and 460 term)	Single foetus deliveries*	DCC	ICC: 5-10 sec	Hemoglobin (newborn cord blood & after 24 hrs), neonatal complications, bleeding volume, third labour

Seidler AL, *et al. BMJ Open* 2020; 10:e034595. doi: 10.1136/bmjopen-2019-034595

iCOMP

Trial Country (PI)	Publication year	Start year/ completion year	Sample size	Participants	Intervention	Comparator	Primary outcome/s
							time, incidence of placental adhesion and peeling
China [20] (Xie)	n/a	2017/2019	300	Singletons, <34 weeks' GA	UCM: x2-3, 25cm/2 sec, below placenta level, before clamping	ICC	Haemoglobin, Haematocrit, and ferritin level at 48 hours
Egypt [21] (Allam)	n/a	2018/2019	210	Singletons, 30-34 weeks' GA	DCC: until cord stops pulsing or 1-2 min	ICC: <5 sec	Fetal haemoglobin, bilirubin, death
Egypt [22] (Nour 2017a)	n/a	2017/2019	90	<34 weeks' GA	UCM: x3 at 10cm/sec, below placenta level, cord held 20-25cm from baby	ICC	Peripheral venous CD34 at admission
Egypt [23] (Nour 2017b)	n/a	2017/2018	90	<34 weeks' GA	Arm 1: ICC, with placental insufficiency<br>Arm 2: DCC at 60sec, with placental insufficiency	Normal placenta with DCC at 60 sec	Peripheral venous CD34 at admission
Germany [24] (Nelle)	1998	n/a	19	PT <1500g*, born by caesarean section	DCC: 30 sec, 30 cm below placenta	ICC	Mean Blood Pressure, left ventricular output, mean cerebral blood flow velocity in the arteria carotis interna, haemoglobin, haematocrit , systemic and cerebral haemoglobin transport, systemic vascular resistance
Germany [25] (Rabe 2000)	2000	2006/2008	40	<33 weeks' GA	DCC: 45 sec	DCC: 20 sec	Feasibility, effects on post-partal adaption and anaemia of prematurity
India [26] (Aghai 2018)	n/a	2018/2020	1400 (not all preterm)	Depressed neonates, 35-42 weeks' GA*	UCM: x4, 30cm over 2 sec	ICC: immediately after birth	Number of infants with moderate to severe HIE or death
India [27] (Anusha)	n/a	2017/2019	148	birth weight <1500g*	DCC: 30 sec	ICC: within 10 sec	Haemodynamic stability, haematological status, serum ferritin, and requirement of

Seidler AL, *et al. BMJ Open* 2020; 10:e034595. doi: 10.1136/bmjopen-2019-034595

iCOMP

Trial Country (PI)	Publication year	Start year/ completion year	Sample size	Participants	Intervention	Comparator	Primary outcome/s
							blood transfusion between birth and 6 months of age
India [28] (Bhriguvanshi)	n/a	2017/2018	236	> 28 weeks' GA, requiring resuscitation*	UCM: x3 towards baby at 10cm/sec, then clamped	ICC: within 30 sec	Haemoglobin and haematocrit at birth and 6 weeks of age
India [29] (Chopra)	2018	2013/2015	142	growth retarded babies (IUGR) ≥ 35 weeks' GA*	DCC: 60 sec	ICC: 10 sec	Haemoglobin and ferritin levels
India [30] (Das/Sundaram)	2018	2012/2013	461	30-33^{6} weeks' GA	DCC: 60 sec below placenta. If baby depressed, immediate clamping keeping cord long, milked x2-3 during resuscitation	ICC: within 10 sec	Composite of mortality or abnormal neurological examination at 40 weeks PMA
India [31] (Datta)	2017	2011/2013	120	Singletons, 34-36^{6} weeks' GA	DCC: at >30-<60 sec	ICC: <20 sec	Neurobehavioural Assessment of Preterm Infant at 37 weeks' post-conceptional age
India [32] (Dhaliwal)	2014	n/a	300	34-37 weeks' GA	DCC: 60 sec	ICC: <10 sec	Risk of neonatal mortality & abnormal neurological examination at 40 weeks' GA
India [33] (Dipak)	2017	2012/2013	78	27-31^{6} weeks' GA	Arm 1: DCC: 60 sec<br>Arm 2: DCC: 60 sec with intramuscular ergometrine	ICC: <10 sec	Hematocrit 4 h after birth
India [34] (George/Isac)	n/a	2017/2018	180 (not all preterm)	Mothers at 34-40^{6} weeks' GA*	UCM: milking whole length at 10cm/sec x3, then clamped	ICC	Infant haemoglobin and haematocrit at 72hrs and 6 weeks
India [35] (Gupta)	n/a	2018/2020	110	<34 weeks' GA	DCC: 30 sec	ICC	Ferritin and PCV at 8 weeks
India [36] (Kumar)	2015	2013/2014	200	32-36^{6} weeks' GA, vaginal or caesarean	UCM: x3, 10cm/sec	ICC	Haemoglobin and ferritin at 1.5 months

Seidler AL, *et al. BMJ Open* 2020; 10:e034595. doi: 10.1136/bmjopen-2019-034595

iCOMP

Trial Country (PI)	Publication year	Start year/ completion year	Sample size	Participants	Intervention	Comparator	Primary outcome/s
India [37] (Ram Mohan)	2018	2015/2016	60	<37 weeks' GA, requiring resuscitation	UCM: 20-25 cm umbilical cord x3 at 10cm/sec within 30 sec of birth	No milking	Haemoglobin and serum ferritin at 6 weeks
India [38] (Rana/Agarwal)	2018	2013/2013	100	<34 weeks' GA	DCC: 120 sec	ICC: ≤30 sec	serum total bilirubin and haematocrit levels at 48 hrs and 7 days
India [39] (Ranjit)	2015	2010/2010	100	30-36[6] weeks' GA	DCC: >2min	ICC	Haematocrit and serum ferritin at 6 weeks
India [40] (Kumar Mangla/ Thukral)	n/a	2016/2017	144 (not all preterm)	Late preterm and term neonates*	Deferred UCM: cord clamped at 60 sec	UCM: Cord milking in 10 sec	Venous haematocrit at 48 hours of life
India [41] (Upadhyay 2010)	2013	2010/2011	170 (not all preterm)	>35 weeks' GA*	UCM: x3 at 10cm/sec, then clamped at ~25 cm of length within 30 sec of birth	ICC: <30 sec	Haemoglobin and serum ferritin at 6 weeks
India [42] (Varanattu)	n/a	2018/2019	250	<32 weeks' GA	UCM: x3 over 20 sec at 20cm/2sec with 2 second pause between	ICC: clamped immediately	Haemoglobin levels at birth and IVH (incidence and severity) at 7 days
Iran [43] (Armanian)	2017	2014/2015	63	≤34 weeks' GA	DCC: at 30-45 sec	ICC: at 5-10 sec	Time of cord clamping
Iran [44] (Mojaveri)	2017	2014/2015	70	<32 weeks' GA, caesarean, birth weight < 1500g, not requiring advanced resuscitation	DCC: at 30-45 sec	ICC: <10 sec	IVH (days 3 to 7), survival infant (up to 28 days)
Iran [45] (Mirzaeian)	n/a	2017/2018	160	28-34 weeks' GA	UCM: milked x3 in 10 sec	ICC	Amount of transfused blood, bilirubin levels
Iran [46] (Sekhavat)	2008	n/a	52	26-34 weeks' GA	DCC: 30-60 sec	ICC: 10-15 sec	Blood pressure, haematocrit, blood glucose

Seidler AL, *et al. BMJ Open* 2020; 10:e034595. doi: 10.1136/bmjopen-2019-034595

iCOMP

Trial Country (PI)	Publication year	Start year/ completion year	Sample size	Participants	Intervention	Comparator	Primary outcome/s
Iran [47] (Shahgheibi)	n/a	2017/2018	90	Women with preterm labour	DCC: 180 sec	DCC: 30 sec	blood parameters, weaning from ventilator, NICU discharge time
Ireland [48] (Dempsey)	n/a	2015/2016	45	<32 weeks' GA	Arm 1: DCC at 60 sec on mobile resuscitation trolley at/below placenta level<br>Arm 2: UCM – Cord stripped 3 times at 20cm/2 sec at/below placenta level	ICC: <20 sec	Neonatal: Brain activity (6 & 12 hours post-partum, EEG and NIRS)<br>Maternal: hemoglobin at 24-36 hours post-partum
Israel [49] (Kugelman)	2007	2004/2005	65	24-35^{6} weeks' GA	DCC: 30-45 sec, below placenta level	ICC: <10 sec	Haematocrit, blood pressure
Italy [50] (Pratesi)	2018	2016/2017	40	23-29^{6} weeks' GA	Bedside assistance with intact placental circulation	UCM: x4, 10cm/sec, before clamping	Feasibility (recruitment rate, compliance, completeness, receiving echographic assessment) and safety
Japan [51] (Hosono 2008)	2008	2001/2002	40	24-28 weeks' GA, singletons	UCM: 20 cm of the cord, x2-3, before clamping, 20cm/2sec	ICC	Probability of not needing transfusion, number of RBC transfusions
Japan [52] (Hosono 2016)	2016	2008/2016	203	24-27^{6} weeks' GA	UCM: cord cut 30 cm from infant, cord milked x1	ICC: <30 sec	1) Probability needing transfusion and death<br>2) Amount of blood transfusion first 4 weeks
Korea [53] (Song)	2017	2012/2015	66	24-32^{6} weeks' GA	UCM: x4 at 20cm/2sec, with 2 sec pause between	ICC: immediately after delivery	Short term safety: Apgar score, prevalence of hypothermia, early intubation, initial blood gas analyses, bilirubin levels, duration of phototherapy, use of cross-transfusion, respiratory distress.

Seidler AL, *et al. BMJ Open* 2020; 10:e034595. doi: 10.1136/bmjopen-2019-034595

iCOMP

Trial Country (PI)	Publication year	Start year/ completion year	Sample size	Participants	Intervention	Comparator	Primary outcome/s
Macedonia [54] (Zisovska)	2008	n/a	57	Premature newborns	DCC: 1 min	ICC	Hematological parameters, number of RBC transfusions
Nepal [55] (Andersson)	2017	2014/2017	540 (not all preterm)	34-41 weeks' GA*	DCC: at ≤180 sec	ICC: ≤30 sec	Haemoglobin at 8±1 months
Nepal [56] (Ashish KC)	n/a	2016/2016	1510 (not all preterm)	Singletons, normal vaginal delivery, ≥33 weeks' GA*	DCC: at 180 sec	DCC: <60 sec	Neonatal heart rate continuously until 10 min after birth and at 1,3&5 min
Netherlands [57] (Knol)	n/a	2018/2019	64	<32 weeks' GA	Physiology-based cord clamping:stabilisation while the cord is intact, cord clamped when infant is respiratory stable (regular spontaneous breathing, heart rate >100 bpm, oxygen>90%, supplemental oxygen <40%)	ICC/DCC: immediately or delayed 30-60 sec, depending on clinical condition of infant	Time needed to stabilise the infant
Netherlands [58] (Te Pas)	n/a	2019/2020	660	<30 weeks' GA	Physiology-based cord clamping: Resuscitation with cord intact, clamp when infant is stable (heart rate >100 bpm, oxygen>85%, supplemental oxygen <40%)	ICC/DCC: immediately or delayed 30-60 sec, depending on clinical condition of infant	Intact survival at NICU discharge without cerebral injury (IVH ≥ grade 2 and/or PVL ≥ grade 2 and/or periventricular venous infarction) and/or NEC (Bell stage ≥ 2)
Netherlands [59] (Ultee)	2008	n/a	37	34 to 36^6 weeks' GA	DCC: 3 min	ICC: <30 sec	blood glucose levels at 1,2, and 3hrs of age, haemoglobin and haematocrit at 1hr and 10 weeks, ferritin at 10 weeks
New Zealand [60] (Meyer/Nevill)	n/a	2016/2020	120	<31 weeks' GA, not breathing regularly at 15sec	Positive pressure ventilation and continuous positive	DCC: 50sec with thermal wrap	RBC transfusion rates

Seidler AL, *et al. BMJ Open* 2020; 10:e034595. doi: 10.1136/bmjopen-2019-034595

iCOMP

Trial Country (PI)	Publication year	Start year/ completion year	Sample size	Participants	Intervention	Comparator	Primary outcome/s
					airway pressure until cord clamping at 50s		
Pakistan [61] (Malik)	2013	2009-2009	80	30-37 weeks' GA	DCC: 120 sec	DCC: 30 sec	Haematocrit
Saudi Arabia [62] (Al-Wassia)	n/a	2017/2019	180	<32 weeks' GA	UCM: milked 20cm segment over 2-3 sec x3	DCC: 60 sec	IVH at 28 days
Saudi Arabia [63] (Gomaa)	n/a	2016/2018	200	24 to 34^6 weeks' GA	DCC: 45-60 sec, baby at level or below placenta	UCM: milked x4-5 from maternal end of cord to baby abdomen, 2 sec pause between milking	Haematological parameters - haematocrit
South Africa [64] (Hofmeyr 1988)	1988	n/a	38	Singleton, <35 weeks' GA	Arm 1: DCC at 60sec<br>Arm 2: DCC at 60sec + ergometrine	ICC	PVH/IVH at 6-72hrs after birth, Apgar score at 5min, birthweight, systolic blood pressure at 5min, cord blood gas and death.
South Africa [65] (Hofmeyr 1993)	1993	n/a	86	<2000 g birthweight*	DCC: 1-2 min	ICC	death of the baby, PVH/IVH at 6-72hrs after birth, Apgar score at 5min, cord-pH, bilirubin
South Africa [66] (Tiemersma)	2015	2012/2012	104 (not all preterm)	Birth weight <2500g ± 500g*	DCC: 2-3 minutes	ICC: within 30 sec	Haemoglobin from cord blood and at 2 months
Spain [67] (De Paco Matallana)	n/a	2011/2014	100	24- 34 weeks' GA	DCC: 45-60 sec	ICC: <10 sec	Neonatal haemoglobin, haematocrit and bilirubin levels (within 7 days after birth)
Spain [68] (Domingo Puiggrós)	n/a	2014/2016	40	<34 weeks' GA, caesarean	UCM: x3 at 20 cm/2sec	DCC: 30 sec	Haemoglobin at 1 and 24 hrs

Seidler AL, *et al. BMJ Open* 2020; 10:e034595. doi: 10.1136/bmjopen-2019-034595

iCOMP

Trial Country (PI)	Publication year	Start year/ completion year	Sample size	Participants	Intervention	Comparator	Primary outcome/s
Spain [69] (Leal)	2019	2013/2016	138	24^0-36^6 weeks' GA	UCM: nearly 20cm cord milked towards umbilicus x4 before clamping	ICC: <20 sec	Requirement of RBC transfusions or phototherapy
Spain [70] (Socias)	n/a	2014/2017	150	26-32^6 weeks' GA	DCC: 30-60 sec	ICC: <30sec	RBC transfusions (number & volume), IVH, postpartum haemorrhage
Switzerland [71] (Baenziger)	2007	1996/1997	39	24-32 weeks' GA	DCC: 60-90 sec, below placenta, syntocinon	ICC: <20 sec	Cerebral oxygenation at 4, 24 and 72 hrs of age
Taiwan [72] (Shen)	n/a	2015/2019	100	<30 weeks' GA	UCM: milked once after ICC, 20cm section at speed of 10cm/sec and clamped at 2-3cm.	ICC and no milking	Neonate's haemoglobin, haematocrit, and mean arterial pressure at admission
Thailand [73] (Chamnanvanakij)	2017	2015/2016	46	25-34 weeks' GA	UCM: x3-4, 30 cm, before clamping	DCC: at 60 sec	Haematocrit level 2 hrs after birth
Thailand [74] (Jomjak)	n/a	2018/2018	110	Singleton, 24-36^6 weeks' GA	DCC	ICC	Haematocrit at 2 and 48 hrs
Thailand [75] (Mungkornkaew)	2015	2014/2014	200 (not all preterm)	Singleton, 34-42 weeks' GA*, vaginal delivery	DCC: 2 minutes	DCC: 1 minute	Fetal haematocrit
Thailand [76] (Panichkul)	n/a	2015/2016	70	34-36 weeks' GA	DCC: at 60 sec	ICC: at 10 sec	Haematocrit 2 hours after birth
Thailand [77] (Prachukthum)	n/a	2016/2018	120	28-33^6 weeks' GA	Arm 1: UCM x3 before cord clamping at 45 sec<br>Arm 2: DCC at 45 sec, followed by UCM x3	DCC: 45 sec	RBC transfusion received
Thailand [78] (Ruangkit)	2019	2016/2017	47	Multiples, 28-36 weeks' GA	DCC: at 30-60 sec	ICC: <10 sec	Haematocrit level at birth
Thailand [79] (Salae)	2016	2014/2015	86	34-36^6 weeks' GA	DCC: at 2 minutes	ICC: within 30 sec	Haematocrit at 48 hours

Seidler AL, *et al. BMJ Open* 2020; 10:e034595. doi: 10.1136/bmjopen-2019-034595

iCOMP

Trial Country (PI)	Publication year	Start year/ completion year	Sample size	Participants	Intervention	Comparator	Primary outcome/s
Thailand [80] (Tanthawat)	n/a	2016/2016	40	<32 weeks' GA	UCM: Cut cord at 30cm, cord milking x1 at 10cm/sec, clamp and cut cord at 1-2cm from umbilical stump	ICC: <10 sec	Haemoglobin and haematocrit level at admission
Turkey [81] (Alan)	2014	2011/2013	44	≤32 weeks' GA ≤1500 g	UCM: at 25-30 cm x3 at 5cm/s before clamping	ICC: <10 sec	Number and volume of packed RBC transfusions received by infant during first 35 days of life
Turkey [82] (Gokmen)	2011	2008/2009	42	24-31^{6} weeks' GA	DCC: 30-45 sec	ICC: 5-10 sec	peripheral blood hematopoietic progenitor cells before any blood product administered to infants
Turkey [83] (Kilicdag)	2015	2012/2013	54	≤32 weeks' GA	UCM: x4 before clamping (20cm/2sec)	ICC	absolute neutrophil counts
Turkey [84] (Silahli)	2018	2015/2016	75	≤32 weeks' GA	UCM: at 20 cm x3, before clamping	ICC: <10 sec	Thymic size
UK [85] (Aladangady)	2006	n/a	46	24-32^{6} weeks' GA	DCC: 30-90 sec, below placenta, oxytocic agent, with ventilation/ resuscitation if necessary	ICC	Infants' blood volume
UK [86] (Duley)	2018	2013/2015	261	<32 weeks' GA	DCC: after at least 2 min	ICC: <20 sec	Death before hospital discharge, intraventricular haemorrhage
UK (Holland)	Not published	1998/2001	?	<33 weeks' gestation	DCC: 40-90 s	ICC (?)	Median arterial/alveolar PO2 ratio over the first 24 hrs of life
UK [87] (Kinmond)	1993	n/a	36	>27 & <32 weeks' GA, vaginal delivery	DCC: 30 sec, 20 cm below placenta	ICC: 10 sec median	Initial packed cell volume, peak serum bilirubin concentrations, red

Seidler AL, *et al. BMJ Open* 2020; 10:e034595. doi: 10.1136/bmjopen-2019-034595

iCOMP

Trial Country (PI)	Publication year	Start year/ completion year	Sample size	Participants	Intervention	Comparator	Primary outcome/s
							cell transfusions, respiratory impairment
England [88] (Medina)	2013	n/a	51	24-31 weeks' GA	DCC	ICC	Haemodynamic parameters, included vena cava blood flow, ventricular outflow, and flow velocity.
UK [89] (Rabe)	2011	2006/2008	58	Singleton, <34 weeks' GA	UCM: x4	DCC: at 30 sec	neonatal blood haematocrit and haemoglobin at 1 hr after birth
USA [90] (Backes)	2016	2009/2013	40	Singletons, 22^5-27^6 weeks' GA	DCC: 30-45 sec, below placenta	ICC: <10 sec	Safety, feasibility, haematological and circulatory outcomes
USA [91] (Bauer)	n/a	2014/2019	300	>24 and <30 weeks' GA	Arm 1: DCC at 45 sec and indomethacin within 6 hrs<br>Arm 2: DCC at 45 sec and placebo within 6 hrs	Arm 3: ICC and indomethacin<br>Arm 4: ICC and placebo	Fraction of survivors with no severe IVH (grades 3 or 4) or PVL within first 60 days of life
USA [92] (Berens)	n/a	2018/2019	100 (not all preterm)	≥35 weeks GA*, at least 1 previous child that received phototherapy for hyperbilirubinemia	DCC: 60 sec	ICC: <15 sec	Neonatal bilirubin level 24 hours after birth
USA [93] (Bienstock)	n/a	2011/2013	22	24^0-32^6 weeks' GA	UCM: x4 over 10 min	ICC	Haemoglobin within 24 hours of birth and through NICU stay
USA [94] (March/deVeciana)	2013	2009/2011	113	Singletons, 24-28^6 weeks' GA	UCM: 10cm, immediately after delivery, ~20cm actively milked x 3	ICC	RBC transfusion within 28 days of life
USA [95] (Driggers)	n/a	2011/2013	2	Infants delivered at 24^0 to 28^6 weeks' GA	Arm 1: DCC at 30 sec<br>Arm 2: UCM x4 in 10 sec	Arm 3: ICC	Adverse neonatal event: composite of BPD, NEC,

iCOMP

Trial Country (PI)	Publication year	Start year/ completion year	Sample size	Participants	Intervention	Comparator	Primary outcome/s
							grade 3 or 4 IVH or PVL, or death prior to discharge
USA [96] (Elimian)	2014	2008/2011	200	Singletons, 24-34 weeks' GA	DCC: at 30-35 sec (3-4 passes of milking toward the neonate was allowed)	ICC: <5 sec	Need for blood transfusion
USA [97] (Ibrahim)	2000	n/a	32	Birthweight 501g-1250g, 24 to <29 weeks' GA	DCC: 20 sec	ICC	Number of blood transfusions
USA [98] (Josephsen)	n/a	2012/2016	80	24-27^6 weeks' GA	UCM: below level of placenta and ~20 cm cord milked x3 over 10-20 sec before clamping	ICC	Haemoglobin and haematocrit concentrations (within 4 hrs birth) Incidence and number blood transfusions until discharge
USA [99] (Katheria 2011)	2014 & 2017	2011/2013	60	<32 weeks' GA	UCM: x3, below placenta, about 20cm of cord over 2 sec	ICC	Superior vena cava flow at 6 hours
USA [100] (Katheria 2013)	2015 & 2017	2013/2018	197	23-31^6 weeks' GA	UCM: x4 at 20 cm/2 sec	DCC: at 45-60 sec	Superior vena cava flow at <12 hrs
USA [101] (Katheria 2016)	2016	2014/2015	150	<32 weeks' GA	CPAP + DCC at 60s	DCC: 60s	Peak haematocrit in first 24 hours of life
USA [102] (Katheria 2017)	n/a	2017/2022	1200	23-32^6 weeks' GA	UCM: x4 at 20cm/2 sec	DCC: at least 60 sec	Incidence of IVH or death at discharge, up to 6 months corrected gestational age
USA [103] (Katheria 2019)	n/a	2019/2020	1000 (not all preterm)	Non-vigorous newborns born at 35-42 weeks' GA*	UCM: x4, entire umbilical length over 2 sec.	ICC: within 30 sec	Admission to NICU in the first 48 hrs of life
USA [104] (Kattwinkel)	n/a	2016/2021	940	23^0-28^6 weeks' GA	DCC: Assisted ventilation (face mask, CPAP or PPV) prior to DCC at 120 sec	DCC: 30-60 sec, assisted ventilation only after cord clamping	IVH (7-10 days)

Seidler AL, *et al. BMJ Open* 2020; 10:e034595. doi: 10.1136/bmjopen-2019-034595

iCOMP

Trial Country (PI)	Publication year	Start year/ completion year	Sample size	Participants	Intervention	Comparator	Primary outcome/s
USA [105] (Krueger)	2015	2012/2013	67	Singletons, 22-31^6 weeks' GA	DCC & UCM: cord milking x4 with 4-5 sec between each, then DCC at 30 sec	DCC: 30 sec, without cord milking	Initial fetal haematocrit
USA [106] (Martin)	n/a	2012/2014	72	Singletons, 23-37 weeks' GA	Arm 1: DCC at 60 sec<br>Arm 2: DCC at 40 sec	DCC: 20 sec	IVH number and severity (15 months)
USA [107] (Mercer 2003)	2003	1998/2001	32	Singletons, 24-31^6 weeks' GA	DCC: 30-45 sec	ICC: 5-10 sec	Initial mean blood pressure on arrival in NICU
USA [108] (Mercer 2006)	2006	2003/2004	72	Singletons, <32 weeks' GA	DCC: 30-45 sec	ICC: 5-10 sec	BPD, suspected NEC
USA [109] (Mercer 2008)	2011 & 2016 & 2018	2008/2014	211	Singletons, 24-31^6 weeks' GA	DCC & UCM: milking x1 then DCC at 30-45 sec. If clamping cannot be deferred, cord milked x2-3 quickly	ICC: <10 sec	IVH, late onset sepsis
USA [110] (Oh)	2011	2000/2001	33	Singletons, 24-27^6 weeks' GA	DCC: at 30-45 sec	ICC: <10 sec	venous haematocrit at 4 hours of age
USA [111] (Perlman)	n/a	2015/2019	150	28-34^6 weeks' GA	DCC: at 60 sec	DCC: at 30 sec	Haematocrit 1 hour after birth
USA [112] (Smith)	n/a	2014/2018	282	23^0-34^6 weeks' GA	UCM: x4, before clamping	DCC: at 30 sec	Haemoglobin & haematocrit in NICU from admission to discharge
USA [113] (Strauss)	2008	n/a	158[101]	≤36 weeks' GA	DCC: 60 sec	ICC	RBC volume/mass, per biotin labelling
USA [114] (Yared/Young)	n/a	2015/2016	39	Very low birth weight (500 to 1500 grams)*	DCC: at 60 sec	DCC: at 30 sec	IVH (during NICU admission up to 6 months)
Thailand [115] (Pongmee)	2010 (abstract)		43	<35 weeks' GA	UCM: x2 along 30 cm after cutting	ICC	Initial haematocrit, need for blood transfusion

* only those born <37 weeks gestation eligible

** PI advised individual participant data not available due to time elapsed since trial

PI = Principal Investigator cm = centimetres sec = seconds min = minutes

iCOMP

NICU = neonatal intensive care unit	GA = gestational age	PMA = postmenstrual age	ICC = immediate cord clamping
DCC = deferred cord clamping	UCM = umbilical cord milking	PBCC = physiological based cord clamping	RBC = red blood cell
CPAP = continuous positive airway pressure	PO2 = partial pressure of oxygen	PPV = positive pressure ventilation	NIRS = near-infrared spectroscopy
PVL = periventricular leukomalacia	ROP = retinopathy of prematurity	BPD = bronchopulmonary dysplasia	EEG = electroencephalogram
IVH = intraventricular haemorrhage	PVH = periventricular haemorrhage	HIE = hypoxic ischemic encephalopathy	
NEC = necrotising enterocolitis	IUGR = intrauterine growth retardation	PCV = polycythaemia	

1. ISRCTN registry website: **Early compared to delayed umbilical cord clamping in very small prematurely born babies: A study to know which one is better for infant health. http://www.isrctn.com/ISRCTN12219110. Accessed August 27, 2018.**
2. ANZCTR registry website: **Resuscitating newborn infants with the umbilical cord intact- The Baby-Directed Umbilical Cord Cutting (Baby-DUCC) Trial. http://www.anzctr.org.au/ACTRN12618000621213.aspx. Accessed 20th February, 2019.**
3. McDonnell M, Henderson-Smart D: **Delayed umbilical cord clamping in preterm infants: a feasibility study**. *Journal of paediatrics and child health* 1997, **33**(4):308-310.
4. ANZCTR website: **CORD CLAMPING STUDY: Early versus delayed cord clamping and its effects on infant heart rate and oxygen saturation in the first minutes after birth. https://www.anzctr.org.au/Trial/Registration/TrialReview.aspx?id=365659. Access 20th February, 2019.**
5. Australian New Zealand Clinical Trials Registry: **Australian Placental Transfusion Pilot Study: investigating standard cord clamping procedures versus three methods of autologous placental blood transfusion in pre term infants. https://www.anzctr.org.au/Trial/Registration/TrialReview.aspx?ACTRN=12609000248268. Accessed February 20, 2019.**
6. Tarnow-Mordi W, Morris J, Kirby A, Robledo K, Askie L, Brown R, Evans N, Finlayson S, Fogarty M, Gebski V *et al*: **Delayed versus Immediate Cord Clamping in Preterm Infants**. *New England Journal of Medicine* 2017, **377**(25):2445-2455.
7. ClinicalTrials.gov registry: **C-UCM and Cerebral Oxygenation and Perfusion. Available at https://clinicaltrials.gov/ct2/show/NCT03748914. Accessed February 20, 2019.**
8. Yasmeen S, Shahidullah M, Mannan M, Dey A, Chowdhury R, Haque A, Hassan MZ: **Iron Status in Early versus Delayed Cord Clamping Groups of Preterm Neonates Delivered in a Tertiary Level Hospital**. *Journal of Armed Forces Medical College, Bangladesh* 2015, **10**(2):62-65.
9. Chu K, Whittle W, Windrim R, Shah P, Murphy K: **The DUC trial: A pilot randomized controlled trial of immediate vs. delayed umbilical cord clamping in preterm infants born between 24 and 32 weeks gestation**. *American Journal of Obstetrics and Gynecology* 2011, **204**(1 Suppl):S201.
10. El-Naggar W, Simpson D, Hussain A, Armson A, Dodds L, Warren A, Whyte R, McMillan D: **Cord milking versus immediate clamping in preterm infants: a randomised controlled trial**. *Archives of Disease in Childhood - Fetal and Neonatal Edition* 2019, **104**(2):F145-F150.
11. Saigal S, O'Neill A, Suraindar Y, Chua LB, Usher R: **Placental transfusion and hyperbilirubinemia in the premature**. *Pediatrics* 1972, **49**(3):406-419.
12. Dai S, Jin X, Qi HJ: **Effect of different cord clamping times on the prognosis of newborns (不同断脐时间对新生儿预后的影响)**. *Modern Pract Med* 2014, **26**:69-71.
13. Dong XY, Sun XF, Li MM, Yu ZB, Han SP: **Influence of delayed cord clamping on preterm infants with a gestational age of <32 weeks [in Chinese]**. *Zhongguo Dang Dai Er Ke Za Zhi* 2016, **18**:635-638.

iCOMP

14. Chinese Clinical Trial Registry website: **Effect of delayed cord clamping versus unbilical cod milking on cebral blood flow in preterm infant: a randomized, double-blind controlled trial. http://www.chictr.org.cn/showprojen.aspx?proj=30981. Accessed February 20, 2019.**
15. Hu X, Xu X: **The Effects of Different Cord Clamping Time in Preterm Infants by Vaginal Delivery [Master's Degree Thesis]** Zhejiang University; 2015.
16. Clinical Trials website: **Immediate VS Delayed Cord Clamping on Newborns**. *https://clinicaltrialsgov/ct2/show/NCT01029496 last accessed August 30, 2018*.
17. Li J, Yu B, Wang W, Luo D, Dai Q-l, Gan X-q: **Does intact umbilical cord milking increase infection rates in preterm infants with premature prolonged rupture of membranes?** *The Journal of Maternal-Fetal & Neonatal Medicine* 2018:1-7.
18. Chinese Clinical Trial Registry website: **Delayed cord clamping prevents respiratory distress of infants delivered by selective cesarean section, a randomized controlled trial. http://www.chictr.org.cn/showprojen.aspx?proj=30199. Accessed February 20, 2019.**
19. Shi W, Peng J, Chen N: **Effect of delayed cord clamping on outcome of delivery**. *Henan Journal of Preventive Medicine* 2017, **28**:85-87.
20. Clinical Trials website: **The Study on Umbilical Cord Milking to Prevent and Decrease the Severity of Anemia in Preterms**. *https://clinicaltrialsgov/ct2/show/NCT03023917 last accessed August 31, 2018*.
21. Australian New Zealand Clinical Trials Registry: **Delayed fetal cord clamping in preterm labour, Risk and benefits. https://www.anzctr.org.au/Trial/Registration/TrialReview.aspx?id=374731. Accessed February 21, 2019.**
22. ClinicalTrials.gov registry: **Impact of Umbilical Cord Milking in Preterm Neonates With Placental Insufficiency**. *https://clinicaltrialsgov/ct2/show/NCT03731611 Accessed February 21, 2019*.
23. ClinicalTrials.gov registry: **Effect of Delayed Cord Clamping in Preterm Neonates With Placental Insufficiency, available at https://clinicaltrials.gov/ct2/show/NCT03731546. Last accessed Feb 21, 2019.**
24. Nelle M, Fisher S, Conze S, Beedgen B, Linderkamp O: **Effects of late cord clamping on circulation in prematures (VLBWI)**. *Pediatric Research* 1998, **44**(3):454.
25. Rabe H, Wacker A, Hülskamp G, Hörnig-Franz I, Schulze-Everding A, Harms E, Cirkel U, Louwen F, Witteler R, Schneider HP: **A randomised controlled trial of delayed cord clamping in very low birth weight preterm infants**. *Eur J Pediatr* 2000, **159**(10):775-777.
26. ClinicalTrials.gov registry: **Umbilical Cord Milking in Neonates Who Are Depressed at Birth (MIDAB). Available at https://clinicaltrials.gov/ct2/show/NCT03657394. Last accessed February 21, 2019.**
27. Clinical Trials Registry India website: **Comparison of advantages and disadvantages of early and delayed umbilical cord clamping in low birth weight newborns. http://ctri.nic.in/Clinicaltrials/pdf_generate.php?trialid=16653&EncHid=&modid=&compid=%27,%2716653det%27 last accessed February 21, 2019.**
28. Clinical Trials Registry India website: **The effects of umbilical cord squeezing in newborn babies requiring some form of intervention to help and support breathing at birth. http://www.ctri.nic.in/Clinicaltrials/pdf_generate.php?trialid=18641&EncHid=&modid=&compid=%27,%2718641det%27, last accessed February 21, 2019.**
29. Chopra A, Thakur A, Garg P, Kler N, Gujral K: **Early versus delayed cord clamping in small for gestational age infants and iron stores at 3 months of age-a randomized controlled trial**. *BMC pediatrics* 2018, **18**(1):234.

30. Das B, Sundaram V, Tarnow-Mordi W, Ghadge A, Dhaliwal LK, Kumar P: **Placental transfusion in preterm neonates of 30–33 weeks' gestation: a randomized controlled trial**. *Journal of Perinatology* 2018, **38**:496–504.
31. Datta V, Kumar A, Yadav R: **A randomized controlled trial to evaluate the role of brief delay in cord clamping in preterm neonates (34-36 weeks) on short-term neurobehavioural outcome**. *Journal of Tropical Pediatrics* 2017, **63**(6):418–424.
32. Dhaliwal LK, Anjumunnisa S, Kumar P, Saha PK, Venkataseshan S: **Effects of placental transfusion in preterm birth**. *J Matern Fetal Neonatal Med* 2014, **27**(S1):369-370.
33. Dipak NK, Nanavat RN, Kabra NK, Srinivasan A, Ananthan A: **Effect of Delayed Cord Clamping on Hematocrit, and Thermal and Hemodynamic Stability in Preterm Neonates: A Randomized Controlled Trial.** *Indian Pediatr* 2017, **54**(2):112-115.
34. Clinical Trials Registry India website: **Effect of umbilical cord milking of late preterm and term infants on maternal and neonatal outcomes in a tertiary care hospital in South India: A randomized control trial. http://www.ctri.nic.in/Clinicaltrials/pmaindet2.php?trialid=19631 last accessed February 21, 2019**.
35. Clinical Trials Registry India website: **Early versus Delayed Cord clamping in IUGR Preterms a Randomised controlled study. http://ctri.nic.in/Clinicaltrials/showallp.php?mid1=25064&EncHid=&userName=015204 last accessed February 21, 2019.**
36. Kumar B, Upadhyay A, Gothwal S, Jaiswal V, Joshi P, Dubey K: **Umbilical Cord Milking and Hematological Parameters in Moderate to Late Preterm Neonates: A Randomized Controlled Trial**. *Indian Pediatr* 2015, **52**(9):753-757.
37. Clinical Trials Registry India website: **Umbilical cord milking in preterm neonates requiring resuscitation: A randomized controlled trial** *http://wwwctrinicin/Clinicaltrials/pmaindet2php?trialid=10609 last accessed August 31, 2018*.
38. Rana A, Agarwal K, Ramji S, Gandhi G, Sahu L: **Safety of delayed umbilical cord clamping in preterm neonates of less than 34 weeks of gestation: a randomized controlled trial**. *Obstetrics & Gynecology Science* 2018, **61**(6):655-661.
39. Ranjit T, Nesargi S, Rao PNS, Sahoo JP, Ashok C, Chandrakala BS, Bhat S: **Effect of Early versus Delayed Cord Clamping on Hematological Status of Preterm Infants at 6 wk of Age**. *The Indian Journal of Pediatrics* 2015, **82**(1):29-34.
40. Clinical Trials Registry India website: **Comparison of Umbilical cord milking with Delayed cord clamping in late preterm and term neonates: Randomized control trial. www.ctri.nic.in/Clinicaltrials/pmaindet2.php?trialid=14204. Accessed August 31, 2018**.
41. CLinical Trials Registry India website: **Effect of cord milking on hemodynemic status and morbidities in neonates more than 35 weeks of gestation**. *http://wwwctrinicin/Clinicaltrials/pmaindet2php?trialid=3182 last accessed August 31, 2018*.
42. ClinicalTrials.gov registry: **Effect of Intact Umbilical Cord Milking on Neonatal and First Year Neurodevelopmental Outcomes in Very Preterm Infants. (Cord Milking)**. *https://clinicaltrialsgov/ct2/show/NCT03200301 last accessed February 22, 2019*.
43. Armanian AM, Ghasemi Tehrani H, Ansari M, Ghaemi S: **Is "Delayed Umbilical Cord Clamping" Beneficial for Premature Newborns?** *Int J Pediatr* 2017, **5**(5):4909-4918.
44. Kazemi MV, Akbarianrad Z, Zahedpasha Y, Mehraein R, Mojaveri MH: **Effects of Delayed Cord Clamping on Intraventricular Hemorrhage in Preterm Infants**. *Iran J Pediatr* 2017 **27**(5):e6570.
45. Iranian Registry of Clinical Trials website: **Investigation and comparison of neonatal complications of two methods of umbilical cord milking and early cord clamping in neonates**. *http://enirctir/trial/29424 last accessed February 22, 2019*.

46. Sekhavat L, Tabatabaii A: **Immediate and delayed cord clamping in infants born between 26 and 34 weeks**. *Journal of Maternal-Fetal and Neonatal Medicine* 2008, **21**((Suppl 1)):181–182.
47. Iranian Registry of Clinical Trials website: **The delayed umbilical cord clamping effects on early outcome in Preterm neonates**. *http://enirctir/trial/17924 last accessed February 22, 2019*.
48. ISRCTN registry website: **Clamping the umbilical cord in premature deliveries (CUPID)**. *http://wwwisrctncom/ISRCTN92719670 last accessed August 27, 2018*.
49. Kugelman A, Borenstein-Levin L, Riskin A, Chistyakov I, Ohel G, Gonen R, Bader D: **Immediate versus delayed umbilical cord clamping in premature neonates born < 35 weeks: a prospective, randomized, controlled study**. *Am J Perinatol* 2007, **24**(5):307-315.
50. Pratesi S, Montano S, Ghirardello S, Mosca F, Boni L, Tofani L, Dani C: **Placental circulation intact trial (PCI-T)-resuscitation with the placental circulation intact versus cord milking for very preterm infants: a feasibility study**. *Frontiers in pediatrics* 2018, **6**:364.
51. Hosono S, Mugishima H, Fujita H, Hosono A, Minato M, Okada T, Takahashi S, Harada K: **Umbilical cord milking reduces the need for red cell transfusions and improves neonatal adaptation in infants born at less than 29 weeks' gestation: a randomised controlled trial**. *Arch Dis Child Fetal Neonatal Ed* 2008, **93**(1):F14-19.
52. UMIN clinical trials registry: **A multicenter randomized control study of the effect of umbilical cord milking**. *https://uploaduminacjp/cgi-open-bin/ctr_e/ctr_viewcgi?recptno=R000001193 last accessed 31 August, 2018*.
53. Song SY, Kim Y, Kang BH, Yoo HJ, Lee M: **Safety of umbilical cord milking in very preterm neonates: a randomized controlled study**. *Obstet Gynecol Sci* 2017, **60**(6):527-534.
54. Zisovska E, Lazarevska L, Spasova L, Zivkovik J: **The effect of delayed cord clamping on the need for blood transfusion: P17**. *Transfusion Alternatives in Transfusion Medicine* 2008, **10**.
55. Clinical Trials website: **Effect of Timing of Umbilical Cord Clamping on Anaemia at 8 and 12 Months and Later Neurodevelopment**. *https://clinicaltrialsgov/ct2/show/NCT02222805 last accessed August 27, 2018*.
56. ISRCTN registry website: **Effect of timing of cord clamping in newborns during first 10 minutes of birth**. *http://wwwisrctncom/ISRCTN10944304 last accessed August 27, 2018*.
57. Knol R, Brouwer E, Klumper F, Van den Akker T, DeKoninck P, Hutten J, Lopriore E, Van Kaam A, Polglase GR, Reiss I: **Effectiveness of Stabilization of Preterm Infants With Intact Umbilical Cord Using a Purpose-Built Resuscitation Table-Study Protocol for a Randomized Controlled Trial**. *Frontiers in pediatrics* 2019, **7**:134.
58. ClinicalTrials.gov registry: **Aeration, Breathing, Clamping Study 3 (ABC3)**. *https://clinicaltrialsgov/ct2/show/NCT03808051 last accessed February 22, 2019*.
59. Ultee CA, van der Deure J, Swart J, Lasham C, van Baar AL: **Delayed cord clamping in preterm infants delivered at 34 36 weeks' gestation: a randomised controlled trial**. *Arch Dis Child Fetal Neonatal Ed* 2008, **93**(1):F20-23.
60. Australian New Zealand Clinical Trials Registry: **Provision of breathing support during delayed cord clamping in preterm infants.** *https://wwwanzctrorgau/Trial/Registration/TrialReviewaspx?id=369228, last accessed November 06, 2019*.
61. Malik AU, Shahnawaz K, Riaz A: **Comparison between the Efficacy of Early and Delayed Umbilical Cord Clamping in Preterm Infants**. *Pak J Med Health Sci* 2013, **7**(4):992-995.

62. ClinicalTrials.gov registry: **Deferred Cord Clamping Compared to Umbilical Cord Milking in Preterm Infants**. *https://clinicaltrialsgov/ct2/show/NCT02996799 last accessed February 22, 2019*.
63. ClinicalTrials.gov registry: **The Hematologic Impact of Umbilical Cord Milking Versus Deferred Cord Clamping in Premature Neonates**. *https://clinicaltrialsgov/ct2/show/NCT03147846 last accessed February 22, 2019*.
64. Hofmeyr GJ, Bolton KD, Bowen DC, Govan JJ: **Periventricular/intraventricular haemorrhage and umbilical cord clamping. Findings and hypothesis.** *S Afr Med J* 1988, **73**(2):104-106.
65. Hofmeyr GJ, Gobetz L, Bex PJM, Van Der Griendt M, Nikodem C, Skapinker R, Delahunt T: **Periventricular/intraventricular hemorrhage following early and delayed umbilical cord clamping. A randomized controlled trial.** . *Online J Curr Clin Trials* 1993(110).
66. Tiemersma S, Heistein J, Ruijne R, Lopez G, van Lobenstein J, van Rheenen P: **Delayed cord clamping in South African neonates with expected low birthweight: a randomised controlled trial**. *Tropical Medicine & International Health* 2015, **20**(2):177-183.
67. ISRCTN registry website: **DElayed COrd CLAmping versus early cord clamping in preterm infants born between 24 and 34 weeks**. *http://wwwisrctncom/ISRCTN66018314 last accessed August 27, 2018*.
68. Clinical Trials website: **Umbilical Cord Milking vs Delayed Cord Clamping in Preterm Infants Born by Cesarean Section**. *https://clinicaltrialsgov/ct2/show/NCT02187510 last accessed August 30, 2018*.
69. Lago Leal V, Pamplona Bueno L, Cabanillas Vilaplana L, Nicolas Montero E, Martin Blanco M, Fernandez Romero C, El Bakkali S, Pradillo Aramendi T, Sobrino Lorenzano L, Castellano Esparza P *et al*: **Effect of Milking Maneuver in Preterm Infants: A Randomized Controlled Trial**. *Fetal diagnosis and therapy* 2019, **45**(1):57-61.
70. Clinical Trials website: **Timing of Umbilical Cord Occlusion in Premature Babies(<33 w). Delayed vs Early. (CODE-P)**. *https://clinicaltrialsgov/ct2/show/NCT02187874 last accessed August 27, 2018*.
71. Baenziger O, Stolkin F, Keel M, von Siebenthal K, Fauchere JC, Das Kundu S, Dietz V, Bucher HU, Wolf M: **The influence of the timing of cord clamping on postnatal cerebral oxygenation in preterm neonates: a randomized, controlled trial**. *Pediatrics* 2007, **119**(3):455-459.
72. ISRCTN registry website: **Can squeezing blood from the umbilical cord into the baby reduce death and illness in premature babies? http://www.isrctn.com/ISRCTN17228884 last accessed February 26, 2019**.
73. Thai Clinical Trials Registry website: **Effect of delayed cord clamping versus cord milking in infants born at < 34 weeks gestation: a randomized controlled trial**. *http://wwwclinicaltrialsinth/indexphp?tp=regtrials&menu=trialsearch&smenu=fulltext&task=search&task2=view1&id=1277 last accessed August 31, 2018*.
74. Thai Clinical Trials Registry website: **Delayed cord clamping reduced anemic outcome in preterm neonate, http://www.clinicaltrials.in.th/index.php?tp=regtrials&menu=trialsearch&smenu=fulltext&task=search&task2=view1&id=3833 last accessed Feb 25, 2019.**
75. Mungkornkaew S, Siwadune T: **The Difference of Hematocrit in Term and Preterm Vaginal Births in Different Timing of Delayed Cord Clamping**. *Thai Journal of Obstetrics and Gynaecology* 2015:223-230.
76. Thai Clinical Trials Registry website: **Effects of delayed versus early cord clamping in late preterm infants: a randomized controlled trial**. *http://wwwclinicaltrialsinth/indexphp?tp=regtrials&menu=trialsearch&smenu=fulltext&task=search&task2=view1&id=1279 last accessed August 29, 2018*.

iCOMP

77. Thai Clinical Trials Registry website: **Comparison of Three Types of Placental transfusion in Preterm Infants: A Randomized Controlled Trial**. *http://wwwclinicaltrialsinth/indexphp?tp=regtrials&menu=trialsearch&smenu=fulltext&task=search&task2=view1&id=4427 last accessed November 06, 2019*.
78. Thai Clinical Trials Registry website: **A Randomized Controlled Trial of Immediate versus Delayed Umbilical Cord Clamping in Preterm Infants of Multiple Births**. *http://wwwclinicaltrialsinth/indexphp?tp=regtrials&menu=trialsearch&smenu=fulltext&task=search&task2=view1&id=2189 last accessed August 29, 2018*.
79. Salae R, Tanprasertkul C, Somprasit C, Bhamarapravatana K, Suwannarurk K: **Efficacy of delayed versus immediate cord clamping in late preterm newborns following normal labor: a randomized control trial**. *J Med Assoc Thai* 2016, **99**(4):S159-S165.
80. Thai Clinical Trials Registry website: **The Effect of One-Time Umbilical Cord Milking and Early Cord Clamping in Preterm Infants: A Randomized Controlled Trial (One-Time Umbilical Cord Milking)**. *http://clinicaltrialsinth/indexphp?tp=regtrials&menu=trialsearch&smenu=fulltext&task=search&task2=view1&id=2347 last accessed August 31, 2018*.
81. Alan S, Arsan S, Okulu E, Akin IM, Kilic A, Taskin S, Cetinkaya E, Erdeve O, Atasay B: **Effects of Umbilical Cord Milking on the Need for Packed Red Blood Cell Transfusions and Early Neonatal Hemodynamic Adaptation in Preterm Infants Born ≤1500 g: A Prospective, Randomized, Controlled Trial**. *Journal of Pediatric Hematology/Oncology* 2014, **36**(8):e493-e498.
82. Gokmen Z, Ozkiraz S, Tarcan A, Kozanoglu I, Ozcimen Emel E, Ozbek N: **Effects of delayed umbilical cord clamping on peripheral blood hematopoietic stem cells in premature neonates**. *Journal of Perinatal Medicine* 2011, **39**:323–329.
83. Kilicdag H, Gulcan H, Hanta D, Torer B, Gokmen Z, Ozdemir SI, Antmen BA: **Is umbilical cord milking always an advantage?** *The Journal of Maternal-Fetal & Neonatal Medicine* 2016, **29**(4):615-618.
84. Silahli M, Duman E, Gokmen Z, Toprak E, Gokdemir M, Ecevit A: **The relationship between placental transfusion, and thymic size and neonatal morbidities in premature infants - A Randomized Control Trial**. *JPMA The Journal of the Pakistan Medical Association* 2018, **68**(11):1560-1565.
85. Aladangady N, McHugh S, Aitchison TC, Wardrop CA, Holland BM: **Infants' blood volume in a controlled trial of placental transfusion at preterm delivery**. *Pediatrics* 2006, **117**(1):93-98.
86. Duley L, Dorling J, Pushpa-Rajah A, Oddie SJ, Yoxall CW, Schoonakker B, Bradshaw L, Mitchell EJ, Fawke JA: **Randomised trial of cord clamping and initial stabilisation at very preterm birth**. *Archives of Disease in Childhood - Fetal and Neonatal Edition* 2018, **103**(1):F6-F14.
87. Kinmond S, Aitchison TC, Holland BM, Jones JG, Turner TL, Wardrop CA: **Umbilical cord clamping and preterm infants: a randomised trial**. *BMJ (Clinical research ed)* 1993, **306**(6871):172-175.
88. Medina IMF: **Late clamping of the umbilical cord in premature neonates: The real haemodynamic benefits**. *Enfermeria Clinica* 2014, **24**(5):305-308.
89. Rabe H, Jewison A, Alvarez RF, Crook D, Stilton D, Bradley R, Holden D: **Milking compared with delayed cord clamping to increase placental transfusion in preterm neonates: a randomized controlled trial**. *Obstetrics & Gynecology* 2011, **117**(2):205-211.
90. Backes CH, Huang H, Iams JD, Bauer JA, Giannone PJ: **Timing of umbilical cord clamping among infants born at 22 through 27 weeks' gestation**. *J Perinatol* 2016, **36**(1):35-40.

Seidler AL, *et al. BMJ Open* 2020; 10:e034595. doi: 10.1136/bmjopen-2019-034595

iCOMP

91. Clinical Trials website: **Effects of Delayed Cord Clamp and/or Indomethacin on Preterm Infant Brain Injury**. *https://clinicaltrialsgov/ct2/show/NCT02221219 last accessed August 27, 2018*.
92. ClinicalTrials.gov registry: **Relationship Between Delayed Cord Clamping at Birth and Neonatal Bilirubin Levels in Parturients With a Prior Child Requiring Therapy for Neonatal Jaundice, https://clinicaltrials.gov/ct2/show/NCT03741803 last accessed February 25th, 2019**.
93. Clinical Trials website: **Milking the Umbilical Cord Versus Immediate Clamping in Pre-term Infants < 33 Weeks**. *https://clinicaltrialsgov/ct2/show/NCT01819532 last accessed August 30, 2018*.
94. March MI, Hacker MR, Parson AW, Modest AM, de Veciana M: **The effects of umbilical cord milking in extremely preterm infants: A randomized controlled trial**. *J Perinatol* 2013, **33**(10):763-767.
95. ClinicalTrials.gov registry: **Delayed Umbilical Cord Clamping Versus Cord Milking in Preterm Neonate, https://clinicaltrials.gov/ct2/show/NCT01393834 last accessed 25th February, 2019.**
96. Clinical Trials website: **A Randomized Controlled Trial of Immediate Versus Delayed Cord Clamping in the Preterm Neonate**. *https://clinicaltrialsgov/ct2/show/NCT00579839 last accessed August 27, 2018*.
97. Ibrahim HM, Krouskop RW, Lewis DF, Dhanireddy R: **Placental transfusion: umbilical cord clamping and preterm infants**. *Journal of Perinatology* 2000, **20**(6):351.
98. Josephsen J, Vlastos E, Potter S, Al-Hosni M: **Milking the umbilical cord in extreme preterm infants**. *Am J Obstet Gynecol* 2014, **10**(1 Suppl):S403-404.
99. Katheria AC, Leone TA, Woelkers D, Garey DM, Rich W, Finer NN: **The effects of umbilical cord milking on hemodynamics and neonatal outcomes in premature neonates**. *J Pediatrics* 2014, **164**(5):1045-1050.
100. Katheria AC, Truong G, Cousins L, Oshiro B, Finer NN: **Umbilical cord milking versus delayed cord clamping in preterm infants**. *Pediatrics* 2015, **136**(1):61-69.
101. Katheria A, Poeltler D, Durham J, Steen J, Rich W, Arnell K, Maldonado M, Cousins L, Finer N: **Neonatal Resuscitation with an Intact Cord: A Randomized Clinical Trial**. *Journal of Pediatrics* 2016, **178**:75-80.e73.
102. ClinicalTrials.gov registry: **Premature Infants Receiving Milking or Delayed Cord Clamping: PREMOD2. Available at https://clinicaltrials.gov/ct2/show/NCT03019367. Accessed August 23, 2019.**
103. ClinicalTrials.gov registry: **Umbilical Cord Milking in Non-Vigorous Infants (MINVI). Available at https://clinicaltrials.gov/ct2/show/NCT03631940. Accessed August 23, 2019.**
104. Clinical Trials website: **VentFirst: A Multicenter RCT of Assisted Ventilation During Delayed Cord Clamping for Extremely Preterm Infants**. *https://clinicaltrialsgov/ct2/show/NCT02742454 last accessed August 27, 2018*.
105. Krueger MS, Eyal FG, Peevy KJ, Hamm CR, Whitehurst RM, Lewis DF: **Delayed cord clamping with and without cord stripping: A prospective randomized trial of preterm neonates**. *Am J Obstet Gynecol* 2015, **212**(3):394.e391-395.
106. Clinical Trials website: **Optimal Timing of Cord Clamping in Preterm Pregnancy Following Vaginal or Cesarean Delivery (CordClamp)**. *https://clinicaltrialsgov/ct2/show/NCT01766908 last accessed August 27, 2018*.
107. Mercer JS, McGrath MM, Hensman A, Silver H, Oh W: **Immediate and delayed cord clamping in infants born between 24 and 32 weeks: a pilot randomized controlled trial**. *Journal of perinatology* 2003, **23**(6):466.

Seidler AL, *et al. BMJ Open* 2020; 10:e034595. doi: 10.1136/bmjopen-2019-034595

108. Mercer JS, Vohr BR, McGrath MM, Padbury JF, Wallach M, Oh W: **Delayed cord clamping in very preterm infants reduces the incidence of intraventricular hemorrhage and late-onset sepsis: a randomized, controlled trial**. *Pediatrics* 2006, **117**(4):1235-1242.
109. Mercer JS, Erickson-Owens DA, Vohr BR, Tucker RJ, Parker AB, Oh W, Padbury JF: **Effects of placental transfusion on neonatal and 18 month outcomes in preterm infants: a randomized controlled trial**. *J Pediatrics* 2016, **168**:50-55.
110. Oh W, Fanaroff AA, Carlo WA, Donovan EF, McDonald SA, Poole WK, Eunice Kennedy Shriver National Institute of Child Health and Human Development Neonatal Research Network: **Effects of delayed cord clamping in very-low-birth-weight infants**. *J Perinatol* 2011, **31**:S68-S71.
111. Clinical Trials website: **Delayed Cord Clamping in Preterm Neonates (DCC)**. *https://clinicaltrialsgov/ct2/show/NCT02478684 last accessed August 29, 2018*.
112. Clinical Trials website: **Delayed Clamping and Milking the Umbilical Cord in Preterm Infants**. *https://clinicaltrialsgov/ct2/show/NCT02092103 last accessed August 31, 2018*.
113. Strauss RG, Mock DM, Johnson KJ, Cress GA, Burmeister LF, Zimmerman MB, Bell EF, Rijhsinghani A: **A randomized clinical trial comparing immediate versus delayed clamping of the umbilical cord in preterm infants: short-term clinical and laboratory endpoints**. *Transfusion* 2008, **48**(4):658-665.
114. ClinicalTrials.gov registry: **Delayed Cord Clamping in Very Low Birth Weight Infants (DCC). Available at https://clinicaltrials.gov/ct2/show/NCT02337088. Accessed August 23, 2019.**
115. Pongmee P, Nuntnarumit P: **Effects of umbilical cord milking on initial hematocrit and the need for blood transfusion in preterm infants. In: Pediatric Academic Societies Annual Meeting; 2010 May 1-4; Vancouver, Canada. 2010**.
