# Supplementary material for: Systematic review and network meta-analysis with individual participant data on cord management at preterm birth (iCOMP): study protocol
Source: BMJ Open. 2020 Mar 29;10(3):e034595. doi: 10.1136/bmjopen-2019-034595 (PMC7170588; doi:10.1136/bmjopen-2019-034595)
Supplement: Supplementary data [file bmjopen-2019-034595supp003.pdf]

iCOMP search strategy

1. We used search results from a prospective meta-analysis we had previously planned to conduct on cord clamping in preterm infants. Regular searches were conducted from 2010 to 2017.
2. We used search results up to November 2018 from a recently updated Cochrane review on cord clamping in preterm infants, on which some of us are authors (Rabe H, Gyte GML, Díaz-Rossello JL, Duley L. Effect of timing of umbilical cord clamping and other strategies to influence placental transfusion at preterm birth on maternal and infant outcomes. Cochrane Database of Systematic Reviews 2019, Issue 9. Art. No.: CD003248. DOI: 10.1002/14651858.CD003248.pub4.)
3. We conducted new independent searches for the period from November 2018 onwards.

Details of all searches are elucidated below.

1. Search methods – for previously planned PMA (up to September 2017)

We regularly searched the World Health Organisation (WHO) International Clinical Trials Registry Platform (ICTRP) from the period January 2010 to September 2017. In total, fourteen searches were conducted over this period, using a combination of the search terms shown below.

1.1 ICTRP

1. placental transfusion
2. cord clamp*
3. umbilical cord clamp*
4. cord milking
5. milking
6. umbilical cord
7. preterm
8. pre-term
9. prematur*

2. Search methods - Cochrane review update (8th November 2018)

The following sources were searched: Cochrane Pregnancy and Childbirth's Trials Register, ClinicalTrials.gov and WHO ICTRP. Further details of each are provided below.

2.1 Pregnancy and Childbirth's Trials Register

- Searched 8 November 2018 by the Cochrane Pregnancy and Childbirth Information Specialist.
- For detailed information about the registry and search strategies, please go to https://pregnancy.cochrane.org/pregnancy-and-childbirth-groups-trials-register.

2.2 ICTRP

cord AND clamp
cord and clamping
cord AND milking
cord AND stripping

2.3 ClinicalTrials.gov

Advanced search

Interventional studies | cord clamping
Interventional studies | cord milking
Interventional studies | cord stripping

3. Search methods (13th February 2019)

We searched Ovid MEDLINE, Embase, EBM Reviews - Cochrane Central Register of Controlled Trials, WHO ICTRP and ClinicalTrials.gov using the search strings below.

3.1 Ovid MEDLINE(R)

1. umbilical-cord.mp. or exp umbilical cord/
2. (Clamp$ OR Milk$).af.
3. (Placenta$ adj2 transfus$).af
4. 2 or 3
5. exp Infant, Premature/ or preterm*.mp.
6. prematur*.mp.
7. exp Infant, Low Birth Weight/ or exp Infant, Very Low Birth Weight
8. exp Infant, Extremely Low Birth Weight
9. 5 or 6 or 7 or 8
10. 1 and 4 and 9
11. limit 10 to (humans and clinical trial, all)
12. limit 11 to ed=20181001-20190213

3.2 Embase

1. umbilical-cord.mp. or exp umbilical cord/
2. (Clamp$ OR Milk$).af.
3. (Placenta$ adj2 transfus$).af
4. 2 or 3
5. exp Infant, Premature/ or preterm*.mp.
6. prematur*.mp.
7. exp Infant, Low Birth Weight/ or exp Infant, Very Low Birth Weight
8. exp Infant, Extremely Low Birth Weight
9. 5 or 6 or 7 or 8
10. 1 and 4 and 9
11. limit 10 to (human and randomized controlled trial)
12. limit 11 to yr="2018 -Current"

3.3 EBM Reviews - Cochrane Central Register of Controlled Trials

1. umbilical-cord.mp. or exp umbilical cord/
2. (Clamp$ OR Milk$).af.
3. (Placenta$ adj2 transfus$).af
4. 2 or 3
5. exp Infant, Premature/ or preterm*.mp.
6. prematur*.mp.
7. exp Infant, Low Birth Weight/ or exp Infant, Very Low Birth Weight
8. exp Infant, Extremely Low Birth Weight
9. 5 or 6 or 7 or 8
10. 1 and 4 and 9
11. limit 10 to yr="2018 -Current"

3.4 WHO ICTRP

**Search string**
Basic search
1. placental transfusion<br>(limit date of registration from 1/11/2018 onwards)
2. cord clamp<br>(limit date of registration from 1/11/2018 onwards)
3. cord clamping<br>(limit date of registration from 1/11/2018 onwards)
4. milking<br>(limit date of registration from 1/11/2018 onwards)
Advanced search
5. Title: umbilical cord<br>Condition: preterm OR premature<br>Recruitment Status: All<br>(limit date of registration from 1/11/2018 onwards)
6. Condition: preterm OR premature<br>Intervention: “umbilical cord”<br>Recruitment Status: All<br>(limit date of registration from 1/11/2018 onwards)

3.5 Clinicaltrials.gov

**Search string**
Basic search
1. Other terms: “placental transfusion”<br>First posted from 11/01/2018 to 02/13/2019 (MM/DD/YYYY)
2. Other terms: “cord clamp”<br>First posted from 11/01/2018 to 02/13/2019 (MM/DD/YYYY)
3. Other terms: “cord clamping”<br>First posted from 11/01/2018 to 02/13/2019 (MM/DD/YYYY)
4. Other terms: milking<br>First posted from 11/01/2018 to 02/13/2019 (MM/DD/YYYY)
5. Condition or disease: Preterm Birth<br>Other terms: “umbilical cord”<br>First posted from 11/01/2018 to 02/13/2019 (MM/DD/YYYY)
