# Supplementary material for: Systematic review and network meta-analysis with individual participant data on cord management at preterm birth (iCOMP): study protocol
Source: BMJ Open. 2020 Mar 29;10(3):e034595. doi: 10.1136/bmjopen-2019-034595 (PMC7170588; doi:10.1136/bmjopen-2019-034595)
Supplement: Author's manuscript [file bmjopen-2019-034595.draft_revisions.pdf]

BMJ Open is committed to open peer review. As part of this commitment we make the peer review history of every article we publish publicly available.

When an article is published we post the peer reviewers' comments and the authors' responses online. We also post the versions of the paper that were used during peer review. These are the versions that the peer review comments apply to.

The versions of the paper that follow are the versions that were submitted during the peer review process. They are not the versions of record or the final published versions. They should not be cited or distributed as the published version of this manuscript.

BMJ Open is an open access journal and the full, final, typeset and author-corrected version of record of the manuscript is available on our site with no access controls, subscription charges or pay-per-view fees (http://bmjopen.bmj.com).

If you have any questions on BMJ Open's open peer review process please email info.bmjopen@bmj.com

BMJ Open

Systematic review and network meta-analysis with individual participant data on Cord Management at Preterm Birth (iCOMP): study protocol

Journal:	*BMJ Open*
Manuscript ID	bmjopen-2019-034595
Article Type:	Protocol
Date Submitted by the Author:	26-Sep-2019
Complete List of Authors:	Seidler, Anna Lene; The University of Sydney, NHMRC Clinical Trials Centre<br>Duley, Lelia; University of Nottingham, Nottingham Clinical Trials Unit<br>Katheria, Anup; Sharp Healthcare<br>De Paco Matallana, Catalina; Clinic University Hospital Virgen de la Arrixaca<br>Dempsey, E; Cork University Maternity Hospital<br>Rabe, Heike; Brighton and Sussex University Hospitals, Academic Department of Paediatrics<br>Kattwinkel, John; University of Virginia<br>Mercer, Judith; University of Rhode Island, College of Nursing<br>Josephsen, Justin; St Louis University School of Medicine<br>Fairchild, Karen; University of Virginia<br>Andersson, Ola; Uppsala Universitet<br>Hosono, Shigeharu; Jichi Ika University Saitama Medical Center<br>Sundaram, Venkataseshan; Postgraduate Institute of Medical Education and Research, Newborn unit, Dept of Pediatrics<br>Datta, Vikram; Lady Hardinge Medical College<br>El-Naggar, Walid; Dalhousie University, Department of Pediatrics<br>Tarnow-Mordi, William; The University of Sydney, NHMRC Clinical Trials Centre<br>Debray, Thomas; University Medical Center Utrecht, Julius Center for Health Sciences and Primary Care<br>Hooper, Stuart B.; Monash University, The Ritchie Centre, Obstetrics & Gynaecology<br>Kluckow, Martin; University of Sydney,<br>Polglase, Graeme; Monash University, The Ritchie Centre, Obstetrics & Gynaecology<br>Davis, Peter; The Royal Women's Hospital, Newborn Research<br>Montgomery, Alan; University of Nottingham, Nottingham Clinical Trials Unit<br>Hunter, Kylie; NHMRC Clinical Trials Centre, University of Sydney<br>Barba, Angie; The University of Sydney, NHMRC Clinical Trials Centre<br>Simes, John; University of Sydney, NHMRC Clinical Trials Centre<br>Askie, Lisa; University of Sydney, NHRMC Clinical Trials Centre
Keywords:	preterm birth, umbilical cord clamping, placental transfusion, umbilical cord milking, individual participant data meta-analysis, network meta-analysis

SCHOLARONE™
Manuscripts

I, the Submitting Author has the right to grant and does grant on behalf of all authors of the Work (as defined in the below author licence), an exclusive licence and/or a non-exclusive licence for contributions from authors who are: i) UK Crown employees; ii) where BMJ has agreed a CC-BY licence shall apply, and/or iii) in accordance with the terms applicable for US Federal Government officers or employees acting as part of their official duties; on a worldwide, perpetual, irrevocable, royalty-free basis to BMJ Publishing Group Ltd ("BMJ") its licensees and where the relevant Journal is co-owned by BMJ to the co-owners of the Journal, to publish the Work in this journal and any other BMJ products and to exploit all rights, as set out in our licence.

The Submitting Author accepts and understands that any supply made under these terms is made by BMJ to the Submitting Author unless you are acting as an employee on behalf of your employer or a postgraduate student of an affiliated institution which is paying any applicable article publishing charge ("APC") for Open Access articles. Where the Submitting Author wishes to make the Work available on an Open Access basis (and intends to pay the relevant APC), the terms of reuse of such Open Access shall be governed by a Creative Commons licence – details of these licences and which Creative Commons licence will apply to this Work are set out in our licence referred to above.

Other than as permitted in any relevant BMJ Author's Self Archiving Policies, I confirm this Work has not been accepted for publication elsewhere, is not being considered for publication elsewhere and does not duplicate material already published. I confirm all authors consent to publication of this Work and authorise the granting of this licence.

For peer review only

**Title**	**Systematic review and network meta-analysis with individual participant data on Cord Management at Preterm Birth (iCOMP): study protocol**
**Author names**	Anna Lene Seidler[1], Lelia Duley[2], Anup Katheria[3], Catalina De Paco Matallana[4], Eugene Dempsey[5], Heike Rabe[6], John Kattwinkel[7], Judith Mercer[8], Justin Josephsen[9], Karen Fairchild[7], Ola Andersson[10], Shigeharu Hosono[11], Venkataseshan Sundaram[12], Vikram Datta[13], Walid El-Naggar[14], William Tarnow-Mordi[1], Thomas P.A. Debray[15], Stuart Hooper[16], Martin Kluckow[17], Graeme Polglase[16], Peter Davis[18], Alan Montgomery[2], Kylie E Hunter[1], Angie Barba[1], John Simes[1] & Lisa Askie[1] on behalf of the iCOMP collaboration
**Author affiliations**	[1] National Health and Medical Research Council Clinical Trials Centre, University of Sydney, Sydney, Australia<br>[2] Nottingham Clinical Trials Unit, University of Nottingham, Nottingham, UK<br>[3] Sharp Healthcare, San Diego, California, USA<br>[4] Clinic University Hospital Virgen de la Arrixaca, Murcia, Spain<br>[5] Cork University Maternity Hospital, Cork, Ireland<br>[6] Brighton and Sussex Medical School, University of Sussex, Brighton, UK<br>[7] University of Virginia, Charlottesville, Virginia, USA<br>[8] The University of Rhode Island, Kingston, Rhode Island, USA<br>[9] St Louis University School of Medicine, St. Louis, Missouri, USA<br>[10] Uppsala University, Sweden<br>[11] Jichi Medical University Saitama Medical Center, Saitama, Japan<br>[12] Post Graduate Institute of Medical Education & Research, Chandigarh, India<br>[13] Lady Hardinge Medical College, New Delhi, India<br>[14] Department of Pediatrics, Dalhousie University, Halifax, Canada<br>[15] Julius Center for Health Sciences and Primary Care, University Medical Center Utrecht, Utrecht, the Netherlands<br>[16] The Ritchie Centre, Obstetrics & Gynaecology, Monash University, Melbourne, Australia<br>[17] Faculty of Medicine and Health, University of Sydney, Sydney, Australia<br>[18] The Royal Women's Hospital, Melbourne, Australia
**Corresponding author**	Correspondence to:<br>Anna Lene Seidler<br>NHMRC Clinical Trials Centre, University of Sydney<br>Locked Bag 77, Camperdown NSW 1450, Australia<br>lene.seidler@ctc.usyd.edu.au<br>+61 2 9562 5082
**Disclaimers**	None.
**Source(s) of support**	Developing the protocol and establishing the collaborative group was supported by the UK National Institute of Health Research (grant RPPG060910107). Funding to undertake data collection and data analysis for the iCOMP Collaboration has been provided by the Australian National Health and Medical Research Council (grant APP1163585).
**Word count**	4,734
**Number of figures and tables**	Number of figures: 2<br>Number to tables: 1
**Conflict of interest declaration**	Lelia Duley, Anup Katheria, Catalina De Paco Matallana, Eugene Dempsey, Heike Rabe, John Kattwinkel, Judith Mercer, Justin Josephsen, Karen Fairchild, Ola Andersson, Shigeharu Hosono, Venkataseshan Sundaram, Vikram Datta, Walid El-Naggar, and William Tarnow-Mordi are Chief Investigators for eligible trials.

For peer review only - http://bmjopen.bmj.com/site/about/guidelines.xhtml

ABSTRACT

Introduction: Timing of cord clamping and other cord management strategies may improve outcomes at preterm birth. However, it is unclear whether benefits apply to all preterm subgroups such as those who usually receive immediate neonatal care. Previous and current trials compare various policies, including immediate cord clamping, time- or physiology-based deferred cord clamping, and cord milking. Individual participant data (IPD) enables exploration of different strategies within subgroups. Network meta-analysis (NMA) enables comparison and ranking of all available interventions using a combination of direct and indirect comparisons.

Objectives: 1) To evaluate the effectiveness of cord management strategies for preterm infants on neonatal mortality and morbidity overall and for different participant characteristics using IPD meta-analysis; and 2) to evaluate and rank the effect of different cord management strategies for preterm births on mortality and other key outcomes using NMA.

Methods and analysis: We will conduct a systematic search of Medline, Embase, clinical trial registries, and other sources for all planned, ongoing and completed randomised controlled trials comparing alternative cord management strategies at preterm birth (before 37 weeks' gestation). IPD will be sought for all trials. First, deferred clamping and cord milking will be compared with immediate clamping in pairwise IPD meta-analyses. The primary outcome will be death prior to hospital discharge. Effect differences will be explored for pre-specified subgroups of participants. Second, all identified cord management strategies will be compared and ranked in an IPD NMA for the primary outcome and the key secondary outcomes intraventricular haemorrhage (any grade) and infant blood transfusions (any). Treatment effect differences by participant characteristics will be identified. Inconsistency and heterogeneity will be explored.

Ethics and dissemination: Approved by University of Sydney Human Research Ethics Committee (2018/886). Results will be relevant to clinicians, guideline-developers and policy-makers, and will be disseminated via publications, presentations, and media releases.

Registration: Australian New Zealand Clinical Trials Registry: ACTRN12619001305112

KEYWORDS

Preterm birth, umbilical cord clamping, umbilical cord milking, placental transfusion, individual participant data meta-analysis, network meta-analysis, prospective meta-analysis

For peer review only

For peer review only - http://bmjopen.bmj.com/site/about/guidelines.xhtml

STRENGTH AND LIMITATIONS OF THIS STUDY

- This will be the most comprehensive review to date of interventions for umbilical cord management in preterm infants and the findings will be highly relevant to clinicians and guideline developers
- The use of individual participant data will allow assessment of the best treatment option for key subgroups of participants
- Network meta-analysis will enable the comparison and ranking of all available treatment options using direct and indirect evidence
- For some of the trials it will not be possible to obtain individual participant data, so published aggregate results will be used instead
- Risk of bias in the primary trials will be assessed using Cochrane criteria, and certainty of evidence for the meta-analyses will be appraised using the GRADE approach for the pairwise comparisons, and the CINeMA approach for the network meta-analysis

For peer review only

For peer review only - http://bmjopen.bmj.com/site/about/guidelines.xhtml

INTRODUCTION

Currently over 15 million babies are born preterm annually and this number is rising.(1-3) Of these, 1.1 million die, and the morbidity and healthcare costs amongst survivors and their families are high, with preterm survivors having an increased risk of cognitive, developmental and behavioural difficulties, and chronic ill health.(4-9) Hence, even modest improvements in outcomes of preterm birth would substantially benefit the children, their families, and also health services. In uncompromised babies, deferring cord clamping has been shown to be beneficial and is now used in routine practice.(10) However, it is unclear whether these benefits apply to preterm babies who usually receive immediate neonatal care, and whether any benefits outweigh potential harms. In addition, there are multiple competing cord management strategies, such as clamping the cord at different times or milking the cord, and considerations of the infant's respiratory status, and it is currently unknown which strategy yields the best balance of benefits and harms.

Current approaches to cord clamping

One potential mechanism of deferring umbilical cord clamping is a net transfer of blood from the placenta to the baby known as "placental transfusion". If the cord is not clamped at birth immediately, blood flow between the placenta and the baby may continue for up to five minutes in term infants.(11-13) For preterm births, blood flow may continue for longer,(14) since a greater proportion of feto-placental circulating blood volume is still in the placenta.(15) This has led to time-based approaches to deferring cord clamping that have been shown to increase peak haematocrit and reduce the need for blood transfusions.(16) Yet, recent findings suggest that placental transfusion does not always occur - blood flow may continue without any net transfer, and sometimes net transfer may be to the placenta.(17) Initial neonatal care and stabilisation traditionally takes place on a resuscitation platform at the side of the room or in an adjacent room. Deferred cord clamping is thus often associated with a delay in neonatal care and this has led to concerns including delayed resuscitation and hypothermia (18) particularly for very preterm infants and infants assessed as requiring resuscitation. An alternate emerging strategy is to provide immediate neonatal care with the cord intact beside the woman using a mobile resuscitation trolley or on the mother's leg. (19-24)

Another potential mechanism of deferred clamping is allowing time for the infant to establish spontaneous breathing whilst still placentally supported. Immediate cord clamping before the infant has established breathing may be harmful since it can lead to large fluctuations in blood pressure, a period of hypoxia, and restricted cardiac function.(25) Animal and pilot human studies suggest that breathing and lung aeration before cord clamping can improve cardiovascular stability and oxygenation, and reduce intraventricular haemorrhage and infant mortality.(26-29) They also suggest that initial respiratory support before clamping the cord can improve cerebral oxygenation and blood pressure, and reduce cerebrovascular impairment compared with immediate cord clamping.(30, 31) This evidence has led to the rise of "physiological cord clamping" which defers clamping until after the onset of breathing. Yet, onset of breathing is not always easy to determine without the assistance of video or extra equipment, whilst timing to cord clamping can be easily measured. In an earlier study,(32) time of onset of breathing in preterm infants receiving gentle stimulation was related to time after birth – within a minute over 90% of preterm infants had begun spontaneous breathing.

For peer review only - http://bmjopen.bmj.com/site/about/guidelines.xhtml

Cord milking or stripping (pinching the umbilical cord close to the mother and moving the fingers towards the infant) may be a way to increase preterm blood volume without deferring clamping.(33) Yet, a preterm lamb model demonstrated that during cord milking there was a transient increase to carotid blood flow and pressure.(34) A recent trial comparing deferred cord clamping with cord milking was stopped early in the subgroup of extremely preterm infants (23-27 weeks), as the incidence of severe intraventricular haemorrhage was higher in the cord milking group.(35) Hence, the effect of cord milking in different populations needs further elucidation.

Current guidelines for cord management at birth and previous reviews of aggregate data

Current uncertainties in optimal cord management strategies are reflected in varying guidelines. The World Health Organization (WHO) recommends late cord clamping (36) unless resuscitation is required, the National Institute for Health and Care Excellence (NICE) recommends waiting for 30 seconds to 3 minutes if mother and baby are stable, (37) and the International Liaison Committee on Resuscitation Council (ILCOR) recommends a delay in cord clamping of at least 1 minute. If the baby is assessed as requiring resuscitation (which is the case in many preterm infants), (38) WHO recommends immediate clamping, (39) NICE recommends considering cord milking before clamping, and ILCOR concludes that there is insufficient evidence to make any recommendations. (38)

A 2012 Cochrane review of timing of cord clamping for preterm births (40) included 15 trials, with 738 infants, of which one trial (with 40 infants) compared cord milking with immediate cord clamping.(41) There was heterogeneity in the timing of cord clamping and gestational age at recruitment, and data were insufficient for reliable conclusions about any of the primary outcomes of the review. A systematic review and meta-analysis published in 2018 (including 18 trials with 2834 infants) compared the effect of deferred (≥30 seconds) versus early (<30 seconds) clamping in preterm infants, and found a reduction in the primary outcome of hospital mortality by 32% (Risk Ratio = 0.68, 95% Confidence Interval = 0.52-0.90). (16) There was heterogeneity in the definition of 'early cord clamping' ranging from less than 5 to 25 seconds, and "late cord clamping", ranging from 30 to 180 seconds. Recruitment age varied from 22 weeks to 36 weeks gestational age. Most analyses of infant and maternal morbidity were substantially underpowered. (16) The review concluded that while there is high quality evidence that deferred cord clamping improves outcomes, individual participant data analyses are urgently needed to further understand the benefits and potential harms of different cord management strategies, and to understand whether differential treatment options are advantageous for key subgroups of infants. (16)

This ongoing uncertainty about the optimal cord management strategy, and differential cord management strategies for key subgroups of infants (e.g. for those for which resuscitation and/or stabilisation is deemed necessary, or extremely preterm infants) has led to 113 planned, ongoing or published trials (in more than 15,000 preterm babies) that are comparing a range of cord management strategies. *Individual participant data (IPD) meta-analysis* is the gold standard for combining such trial data. IPD provides larger statistical power for estimation of treatment effects of rarer secondary endpoints and enables reliable subgroup analyses to examine hypotheses about differences in treatment effect, exploring interactions between treatment- and participant-level characteristics. (42) A *network meta-analysis (NMA)* facilitates data synthesis when there are a range of interventions available and permits indirect comparisons across all interventions by inferring the relative effectiveness of two competing treatments through a common comparator.(43, 44) NMA produces relative effect

For peer review only - http://bmjopen.bmj.com/site/about/guidelines.xhtml

estimates for each intervention compared with every other intervention in the network. These effect sizes can be used to obtain rankings of the effectiveness of the interventions.(45) Using IPD in a NMA (as opposed to aggregate data) can improve precision, increase information, and reduce bias. (46)

Objectives

The aims of this study are:

1) to evaluate the effectiveness of cord management strategies for preterm infants on neonatal mortality and morbidity, and to evaluate patient-level modifiers of treatment effect.
2) to evaluate, compare and rank the effectiveness of different cord management strategies for preterm infants on mortality and the key secondary outcomes intraventricular haemorrhage (any grade) and infant blood transfusions (any).

METHODS AND ANALYSIS

We will conduct a systematic review of randomised trials with individual participant data using pairwise and network meta-analysis, and a nested prospective meta-analysis. The lead investigator for all potentially eligible studies will be contacted and invited to collaborate and join the **i**ndividual participant data **Co**rd **M**anagement at **P**reterm birth (iCOMP) Collaboration. Eligible trials identified up to February 2019 are listed in Supplementary File 1. The Collaboration will undertake this project according to the methods recommended by the Cochrane Individual Participant Data, Multiple Interventions, and Prospective Meta-Analysis Methods Groups.(42, 47, 48) This protocol is registered on the Australian New Zealand Clinical Trials Registry (ANZCTR) and is undergoing editorial review for registration on the International Prospective Register of Systematic Reviews (PROSPERO). Reporting guidelines for NMA protocols by Chaimani et al (49) and PRISMA extension for IPDs and protocols (50, 51) have been followed for reporting (checklist provided in Supplementary File 2).

Eligibility criteria

Types of studies

Studies will be included if they are randomised trials. Cluster-randomised and quasi-random studies will be excluded. Studies must compare at least two of the interventions of interest (defined below).

Trial participants

Participants will be women giving birth preterm (before 37 completed weeks' gestation) and/or their babies. Individually randomised studies will be eligible for inclusion if the unit of randomisation was either the woman, or the baby. Women and babies will be included regardless of whether mode of delivery was vaginal or caesarean, and whether the birth was singleton or multiple. Babies will be included regardless of whether or not they received immediate resuscitation at birth.

Types of interventions and comparators in pairwise meta-analysis

For the pairwise meta-analysis we will include all trials that compare an intervention to enhance umbilical blood flow or allow more time for physiological transition to the comparator immediate cord clamping. This includes interventions assessing cord management strategies for timing of cord clamping,

For peer review only - http://bmjopen.bmj.com/site/about/guidelines.xhtml

and other strategies such as cord milking. Trials will be included regardless of whether initial neonatal care is provided with the umbilical cord intact, or not. Different strategies (i.e. deferred cord clamping and cord milking) will be analysed in separate subgroups to assess comparability between the groups by assessing subgroup effects and heterogeneity. They will then be collapsed into one "cord management intervention" group if they are deemed comparable based on the previous subgroup assessments. If they are deemed non-comparable they will be analysed and interpreted separately.

Types of interventions and comparators in network meta-analysis

For the network meta-analysis we will include, as interventions of interest, strategies for timing of cord clamping, and other cord management strategies to influence umbilical flow and placental transfusion.

Thus, interventions of interest include:

- Immediate cord clamping without milking (≤15 seconds or trialist defined)
- Short deferral of cord clamping (>15 to <45 seconds) without milking
- Medium deferral of cord clamping (≥45 to <90 seconds) without milking
- Long deferral of cord clamping (≥ 90 seconds) without milking
- Cord milking or stripping before immediate cord clamping (intact cord milking)
- Cord milking or stripping before deferred cord clamping (intact cord milking)
- Cord milking or stripping after immediate cord clamping (cut cord milking)
- Cord milking or stripping after deferred cord clamping (cut cord milking)
- Physiological clamping (clamping after aeration of lungs)

If we identify other interventions not listed above we will include them if they are addressing cord management or related strategies to influence umbilical flow and placental transfusion. Again, trials will be included regardless of whether initial neonatal care is provided with the umbilical cord intact, or not. Studies evaluating collection and storage of residual placental blood that is then used for transfusion after birth will be excluded. All possible comparisons between eligible interventions are displayed in Figure 1. For interpretation purposes, immediate cord clamping will act as the basis comparison/ parameter.

Nodes that specify different timings of cord clamping were defined according to what timing is classified as immediate clamping, short deferral, medium deferral or long deferral according to the literature to date (as shown in Supplementary File 1), and after discussion with clinicians. Different timings are commonly compared in head-to-head comparisons, hence, their classification as different intervention nodes. Similarly, nodes that specify cord milking were classified after a review of current milking techniques described in the literature and after discussion with clinicians. If insufficient data are available, categories will be collapsed where possible. For instance, milking before and after immediate cord clamping could be collapsed into one single immediate cord milking category, or medium and long delay could be collapsed into a medium to long delay category. We consider the interventions of interest to be jointly randomisable (i.e. each participant could, in principle, be randomised to any one of the interventions of interest).

For peer review only - http://bmjopen.bmj.com/site/about/guidelines.xhtml

Types of outcome measures

Trials must report at least one of the clinical outcomes included in this review, as specified in the "measures" section below, to be included.

Eligibility for nested prospective meta-analysis

Studies are only included in the nested prospective meta-analysis if the investigator/s were blind to outcome data by intervention group at the time the main components of the protocol (i.e. objectives, aims and hypotheses, eligibility criteria, subgroup and sensitivity analyses and main outcomes) were initially agreed in January 2015.

Information sources and search strategy

The search strategy to identify potentially eligible studies will include a search of the Cochrane Collaboration Pregnancy and Childbirth Review Group's Trial Register. This register contains trials identified from: monthly searches of the Cochrane Central Register of Controlled Trials (CENTRAL) and CINAHL (EBSCO); weekly searches of Medline (Ovid) and Embase (Ovid); hand searches of specialty journals and major conferences proceedings; and current awareness alerts from further journals and BioMed Central. Further details can be found elsewhere.(52) We will identify ongoing trials that may be eligible by searching for published protocols in Medline and Embase, searching online registries of clinical trials, and personal contacts (for example, by asking collaborators to notify any unregistered studies they are aware of). The Chief Investigators of eligible trials will be invited to join the iCOMP Collaboration. They will also be asked if they know of any further planned, ongoing or completed studies. The search strategy is outlined in more detail in Supplementary File 3.

Selection of studies for inclusion in the review

Two members of the iCOMP Secretariat (see project management section below) will independently assess all the potentially eligible studies identified for inclusion. Disagreements will be resolved by discussion or, if required, by consulting a third member of the iCOMP Secretariat. Studies that are not willing or able to provide IPD will be synthesised where possible using aggregate data.

Data collection, management, and confidentiality

Data receipt

De-identified, individual participant level data will be provided by each participating trial. These data will be backed-up and stored in a centralised secure database.

Data processing

Data checking: For each trial, range and internal consistency of all variables will be checked. Intervention details and missing data will be checked against any protocols, published reports, and data collection sheets. Integrity of the randomisation process will be assessed by examining the chronological randomisation sequence and the balance of participant characteristics across intervention groups. Any inconsistencies or missing data will be discussed with the trialists and resolved by consensus. Each included study will be analysed separately and the results sent to the trial investigators for verification

For peer review only - http://bmjopen.bmj.com/site/about/guidelines.xhtml

prior to inclusion in the iCOMP database. All trial-specific outcomes generated from the IPD will be cross-checked against published information via a series of crosstabs.

Data re-coding: Outcome data may have been collected in different formats across trials. Therefore, the de-identified data from each of the trials will be extracted and re-formatted into a commonly coded dataset.

Data transformation and collating: Once the data from each of the trials are finalised, they will be combined into a single dataset, but a trial identifier code for each participant will be retained. New variables will be generated from the combined dataset as required to address the hypotheses to be tested.

Risk of bias assessment and certainty of evidence appraisal

Eligible studies will be assessed for risk of bias using the criteria described in the Cochrane Handbook(53): random sequence generation; allocation concealment; blinding of participants and personnel; blinding of outcome assessment; incomplete outcome data; selective reporting; and other bias. Certainty of evidence will be appraised using the GRADE approach (54) for the pairwise comparisons, and the rating approach suggested by Salanti and colleagues that is implemented in the CINeMA application for the network meta-analysis.(55)

Outcomes measures for pairwise meta-analysis

All outcome measures for the pairwise meta-analysis are listed in Table 1. The primary outcome will be death of the baby prior to hospital discharge. As outcomes for babies born very preterm (before 32 weeks' gestation) are different to those born moderately preterm (32 to 37 weeks), separate analyses will be conducted for these two groups of infants for the secondary outcomes. Where possible, definitions will be standardised, otherwise outcomes will be used as defined in individual trials. Secondary outcomes will include measures of neonatal and maternal morbidity, and health service use.

Covariates and subgroups for pairwise meta-analysis

Subgroup analyses will be conducted for the primary outcome of death (prior to hospital discharge) and two key secondary outcomes (IVH any grade and any infant blood transfusion) if sufficient data are available. All included covariates and subgroups are listed in Table 1. The comparative effects of alternative cord management strategies may vary depending on key infant risk factors, and/or on the level and type of neonatal care available at the hospital of birth. Thus, there will be subgroup analyses based on participant level characteristics and based on hospital level characteristics. If data are insufficient for the pre-specified subgroup analyses, categories will be collapsed.

For peer review only - http://bmjopen.bmj.com/site/about/guidelines.xhtml

Table 1. Measures for individual participant data pairwise meta-analysis

**Outcomes**	
For all infants	
Primary outcome	• Death prior to hospital discharge
For infants born before 32 weeks' gestation	
Key secondary outcomes	• Death (at any time during duration of follow-up)<br>• Severe intraventricular haemorrhage on cranial ultrasound (grade 3-4)<br>• All grades of intraventricular haemorrhage on cranial ultrasound<br>• Necrotizing enterocolitis ≥ grade 2 (or trialist definition)<br>• Late onset sepsis (where possible defined as clinical sepsis > 72 hour after birth)<br>• Patent ductus arteriosus requiring treatment (medical and/or surgical)<br>• Chronic lung disease (at 36 weeks' postmenstrual age or trialist defined)
Other secondary outcomes	• Death (within 7 days)<br>• Other forms of white matter brain injury (e.g. periventricular leukomalacia, porencephaly)<br>• Respiratory support (mechanical ventilation, CPAP, low nasal flow oxygen)<br>• Duration of respiratory support<br>• Supplemental oxygen at 36 weeks<br>• Retinopathy of prematurity requiring treatment (medical and/or surgical)<br>• Drug treatment for hypotension (yes/no)<br>• Blood transfusion (yes/no)<br>• Blood transfusion (number, volume)<br>• Hypothermia on admission to neonatal unit<br>• Haemoglobin<br>• Haematocrit<br>• Polycythaemia<br>• Jaundice requiring treatment<br>• Birthweight<br>• Length of stay in Neonatal Intensive Care Unit (NICU)/ Special Care Unit (SCU)<br>• Length of stay in hospital<br>• Apgar scores at 1 and 5 minutes<br>• Long term developmental disability (assessed using the Bayley III, and/or other tools):<br>○ cerebral palsy<br>○ neurodevelopmental disability<br>○ score on cognitive scale<br>○ score on language scale<br>○ score on social/emotional scale<br>○ score on motor scale<br>○ score on behaviour scale<br>○ deafness<br>○ blindness
For infants born at or after 32 weeks' gestation	
Key secondary outcomes	• Death at any time (during duration of follow-up)<br>• Admission to NICU<br>• Blood transfusion (any, number, volume)
Other secondary outcomes	• Death (within 7 days)<br>• Haemoglobin<br>• Haematocrit<br>• Length of stay in NICU/SCU<br>• Length of stay in hospital<br>• Duration of respiratory support (mechanical ventilation, CPAP, low flow nasal oxygen)<br>• Chronic lung disease<br>• Late onset sepsis (> 72 hour after birth)<br>• Patent ductus arteriosus requiring treatment (medical and/or surgical)<br>• Drug treatment for hypotension

For peer review only - http://bmjopen.bmj.com/site/about/guidelines.xhtml

	• Hypothermia on admission to neonatal unit or postnatal ward<br>• Apgar score at 1 and 5 minutes<br>• Long term developmental disability (assessed using the Bayley III, and/or other tools):<br>○ cerebral palsy<br>○ neurodevelopmental disability<br>○ score on cognitive scale<br>○ score on language scale<br>○ score on social/emotional scale<br>○ score on motor scale<br>○ score on behaviour scale<br>○ deafness<br>○ blindness
For all women	
Secondary outcomes	• Maternal death<br>• Postpartum haemorrhage<br>• Postnatal sepsis requiring treatment<br>• Manual removal of placenta<br>• Retained placenta<br>• Not breast feeding when baby discharged from hospital<br>• Postnatal depression<br>• Blood transfusion
**Covariates/ Subgroups**	
Participant-level characteristics	
	• Gestation at birth<br>• Type of pregnancy: singleton; multiple<br>• Maternal age<br>• Mode of birth: caesarean before onset of labour; caesarean after onset of labour; vaginal<br>• Onset of labour: spontaneous onset or spontaneous prelabour ruptured membranes; not spontaneous onset or spontaneous prelabour ruptured membranes; not known whether spontaneous onset of labour or spontaneous prelabour ruptured membranes<br>• Type of breathing onset: spontaneous breathing onset; supported lung aeration (ventilation); unknown<br>• Time of breathing onset relative to cord clamping: before cord clamping/milking; after cord clamping/milking; unknown<br>• Sex (male, female, uncertain/other)<br>• Ethnicity (trialist defined)<br>• Small for gestational age (trialist defined): yes/no<br>• Maternal antenatal/intrapartum sepsis requiring treatment (trialist defined): yes/no<br>• Assessed as needing resuscitation and/or stabilisation (yes/no)<br>• Type of uterotonic drug (if any)
Hospital / trial-level characteristics	
	• Highest level of neonatal unit available at site: neonatal intensive care unit, neonatal unit (some capacity to provide ventilation), special care baby unit (no ventilation available), no neonatal unit or special care baby unit<br>• Planned timing of uterotonic drug: before cord clamping; after/at cord clamping; timing mixed or not known<br>• Planned position of the baby relative to the placenta whilst cord intact: level with placenta (between level of woman's bed and her abdomen/anterior thigh); more than 20 cm below level of placenta; position mixed or not known<br>• Need for immediate resuscitation at birth: infants requiring immediate resuscitation at birth excluded; infants requiring immediate resuscitation at birth included; unclear whether infants requiring immediate resuscitation at birth included or excluded<br>• Type of consent waiver of consent: deferred consent; informed consent or assent; type of consent unclear<br>• Study year

For peer review only - http://bmjopen.bmj.com/site/about/guidelines.xhtml

Data analysis for pairwise meta-analysis

The full Statistical Analysis Plan will be agreed on by the iCOMP Collaboration before any analyses are undertaken. Analyses will include all randomised participants for which data are available, and the primary analyses will be based on intention-to-treat. Analyses will be conducted using the open-source software R.(56)

For each outcome, a one-stage approach to analysis will be employed to include individual participant data from all eligible trials in a multilevel random or mixed effects regression model. Aggregate data will be included were individual participant data are unavailable.(57) Relative heterogeneity of treatment effects across trials will be estimated using I^2, with further inclusion in secondary models of participant level and trial level covariates to explain the sources of heterogeneity. Prediction intervals will be estimated to ascertain absolute heterogeneity. Forest plots will be presented by trial for the primary outcome, and for any secondary outcomes where there is evidence of heterogeneity across trials.

We will use a generalised linear modelling framework, with the choice of outcome distribution and link function dependent on outcome type. For example, binomial with log link will be used to estimate risk ratios for binary outcomes, and Gaussian with identity link for mean differences, with log-transformation of the data if appropriate. We will follow a similar approach for secondary outcomes. For estimation of subgroup effects, we will present forest plots of pooled treatment effects according to pre-specified subgroup variables, and estimate effects by including appropriate interaction terms between a subgroup variable and treatment arm in the regression models. The results of all comparative analyses will be presented using appropriate estimates of treatment effect along with 95% confidence intervals and two-sided p-values.

In advance of conducting the analyses, we will decide whether there are sufficient reliable data to allow meaningful analysis of any individual outcome or subgroup. If not, the analysis will not be conducted, and this will be reported subsequently.

Outcome measures for network meta-analysis

The primary outcome for the network meta-analysis will be death of the baby during the initial hospital stay. If data availability permits, IVH (any grade) and blood transfusion (any) will be analysed as two key secondary outcomes.

Covariates and subgroups for network meta-analysis

Gestational week at birth and highest level of available care will be considered as effect modifiers to improve consistency of the NMA model. There will be subgroup analyses assessing treatment effect by week of gestational age, and by comparing babies assessed as in need of immediate resuscitation versus not in need of immediate resuscitation.

Assessment of the transitivity assumption for network meta-analysis

Transitivity in the network will be assured by only including interventions that are regarded as jointly randomisable and by limiting our sample to preterm infants. Gestational age at birth, hospital setting

For peer review only - http://bmjopen.bmj.com/site/about/guidelines.xhtml

(highest level of available neonatal care), as well as study year may act as effect modifiers and could influence the transitivity of the network. We will therefore investigate whether these variables are distributed evenly across comparisons. If we find any of those variables to be unevenly distributed, they will be included in the network as covariates to investigate their influence on the network and on possible inconsistency.

Data analysis for network meta-analysis

As for the pairwise meta-analysis, all analyses will be specified a-priori in a full Statistical Analysis Plan, all randomised participants for which data are available will be included, and the primary analyses will be intention-to-treat.

We will calculate a two-step random-effects contrast-based network meta-regression to compare and rank all available interventions for the primary outcome death (during initial hospital stay) and, if data permits, for the two key secondary outcomes - IVH (any grade) and blood transfusion (any). Summary risk ratios with confidence and prediction intervals will be presented for each pairwise comparison in a league table. We will estimate the ranking probability of each intervention being at each rank, and we will use surface under the cumulative ranking curve (SUCRA) and mean ranks to obtain a treatment hierarchy. A frequentist approach to analysis will be used. Should models not converge, a Bayesian approach will be used instead, setting a prior of no effect and a large variance. Correlations induced by multi-arm studies will be accounted for using multivariate distributions.

As a second step, interactions between key covariates and effect estimates will be tested, assuming a common interaction across comparisons. If there are statistically significant interactions between covariates and treatment effects, we will provide probability rankings of intervention effects by subgroup for these covariates. A single heterogeneity parameter will be assumed for each network.

Assessment of inconsistency for network meta-analysis

Global consistency will be assessed using the Q statistics for inconsistency and the design-by-treatment interaction model. Local consistency will be assessed using the loop-specific approach and the node-splitting approach to explore sources of inconsistency. Since tests of inconsistency are known to have low power,(58) results will be interpreted with caution, and potential known sources for inconsistency will be explored even if there is no statistical evidence of inconsistency. Any detected inconsistency will be explored by including covariates into the model, and by excluding potential outlier studies in sensitivity analyses. A judgement of excessive heterogeneity or inconsistency would prevent the interpretation and reporting of the network meta-analysis.

Assessment of compliance with the allocated intervention

Compliance with the interventions will be described for each trial. For studies of early versus deferred cord clamping this will be based on i) the time to cord clamping in each allocated group, and ii) the difference in time between early and deferred clamping. For studies comparing cord milking with no milking, this will be based on i) time to cord clamping in the allocated groups, and ii) reported compliance with cord milking in both groups.

For peer review only - http://bmjopen.bmj.com/site/about/guidelines.xhtml

Assessment of selection bias

We will perform a nested prospective meta-analysis as a sensitivity analysis, to detect potential differences between prospectively and retrospectively included studies that may point to selection or publication bias. We expect to also be able to include some unreported outcomes sourced from the individual participant data provided by the included studies, alleviating selective outcome reporting bias. Additionally, comparison-adjusted and contour-enhanced funnel plots(59) will be utilised to examine whether there are differences in results between more and less precise studies.

Adjustments for multiple testing

There is only one primary outcome, and few key secondary outcomes for this study. For other secondary outcomes, no formal adjustments for multiple testing are planned but instead, we will be following the approach outlined by Schulz and Grimes(60): as secondary outcomes examined in this study are interrelated, we will interpret the pattern of results, examining consistency of results across related outcomes, instead of focusing on any single, statistically significant result. All secondary outcomes will be reported. Subgroup analyses will be performed by testing for interactions and findings will be reported as exploratory.(61)

Planned sensitivity analyses

To assess whether results are robust to trial characteristics and methods of analysis, the following sensitivity analyses will be conducted for the primary outcome, if data are sufficient:

- Excluding studies with high risk of bias for sequence generation and/or concealment of allocation and/or loss to follow up for pairwise and network meta-analysis;
- For trials comparing early cord clamping with deferred clamping, analysis of outcomes weighted by degree of separation in mean actual timing of cord clamping between intervention and control groups for pairwise meta-analysis;
- Analysis of outcomes weighted by degree of separation in haemoglobin (at 24 hours) achieved between intervention and control groups for pairwise meta-analysis (as a surrogate for net placental transfusion);
- For trials with deferred cord clamping, an additional dose-response analysis assessing intended time of cord clamping deferral as a continuous variable will be performed;
- Exploratory analysis based on actual, rather than intended, timing of cord clamping for individual participants for pairwise and network meta-analysis;
- The impact of missing data on the effects of the included interventions for the primary outcome may be explored (if appropriate).

Project management

The iCOMP Collaboration will invite membership from representatives of each of the included trials contributing individual participant data, have a Secretariat, and invite methodological and clinical experts who will form an Advisory Group. The Secretariat will be responsible for data collection, management and analysis, and for communication within the Collaboration.

Ethical issues

For peer review only - http://bmjopen.bmj.com/site/about/guidelines.xhtml

For each included trial, ethics approval has been previously granted by their respective Human Research Ethics Committees (or equivalent), and informed consent has been obtained from all participants. The Chief Investigators of the included trials remain the custodians of their own trial's data. Individual participant data from the included trials will be de-identified before sharing with the iCOMP Collaboration. Ethics approval for this project has been granted by the University of Sydney Human Research Ethics Committee (number: 2018/886).

Publication policy

The key methods for this meta-analysis protocol were agreed by the iCOMP Collaborators in January 2015, before unblinding of any outcome data from the studies included in the nested prospective meta-analysis. This manuscript was discussed at the iCOMP Collaborators' meeting held at the Pediatric Academic Societies meeting in San Diego in April 2015. At this meeting it was agreed the protocol should be expanded to include a retrospective systematic review and individual participant data and network meta-analysis with a nested prospective meta-analysis. The protocol was then revised, based on further discussion, and circulated to members of the iCOMP Collaboration for further comment and agreement prior to manuscript submission.

Participating trialists in the prospective meta-analysis, when reporting results from their own trials, will endeavour to include a statement that their trial is part of this prospective meta-analysis in any published manuscripts or conference abstracts. Any reports of the results of this meta-analysis will be published either in the name of the collaborative group, or by representatives of the collaborative group on behalf of the iCOMP Collaboration, as agreed by members of the collaborative group. Draft reports will be circulated to the collaborative group for comment and approval before submission for publication.

DISCUSSION

There is an urgency to conduct this systematic review and pairwise individual participant data and network meta-analysis so we can make sense of the numerous trials currently being undertaken, inform clinical practice, and identify the most promising interventions for further evaluation.

This meta-analysis offers an opportunity to reliably test important hypotheses that cannot be resolved by any of the individual trials, either alone or in simple combination. Coordinating international efforts in this way will help achieve consensus on the most important substantive clinical outcomes to assess in any future trials as needed. Unequivocal synthesised results, together with the identification of key determinants (e.g. effect modifiers), will be critical for translating evidence from this meta-analysis directly into practice. Figure 2 shows the network of comparisons available from the trials identified to date. We plan to complete study identification and individual participant data collection by end-2019, then conduct the analyses and disseminate results by mid-2021.

This study is only possible because trialists around the world have agreed to collaborate to share the individual participant data from their cord management trials. This collaborative approach will enable us to move beyond the traditional "one-size-fits-all" and towards precision medicine, to find the optimal intervention from a range of treatment options for each individual woman and her baby, based on their individual characteristics and risk factors.

For peer review only - http://bmjopen.bmj.com/site/about/guidelines.xhtml

Patient and Public Involvement

We will invite patients and the public to comment on this research project, to increase its accessibility from their perspective.

Acknowledgements

Thanks to Sarah Somerset, Min Yang, Charlotte Lloyd, and Virginia Portillo for previous support for the Secretariat and input into earlier protocol drafts.

Competing interests

None known.

Non-financial competing interests

Lelia Duley, Anup Katheria, Catalina De Paco Matallana, Eugene Dempsey, Heike Rabe, John Kattwinkel, Judith Mercer, Justin Josephsen, Karen Fairchild, Ola Andersson, Shigeharu Hosono, Venkataseshan Sundaram, Vikram Datta, Walid El-Naggar, and William Tarnow-Mordi are Chief Investigators for potentially eligible trials.

Funding

Developing the protocol and establishing the collaborative group was supported by the UK National Institute of Health Research with a grant entitled "The Preterm Birth Programme" (number RPPG060910107). This grant presents independent research commissioned by the National Institute for Health Research (NIHR) under its Programme Grants for Applied Research funding scheme. The views expressed are those of the authors and not necessarily those of the NHS, the NIHR or the Department of Health. Funding for individual trials remains the responsibility of the trialists themselves. Funding to undertake data collection and data analysis for the iCOMP Collaboration has been provided by the Australian National Health and Medical Research Council via a Project Grant (APP1163585).

Author contributions

LD and LA conceived the idea. ALS, LA, and LD drafted the protocol with input from all authors. All authors have agreed the final manuscript. ALS is the guarantor.

List of abbreviations

iCOMP – **i**ndividual participant data on **Co**rd **M**anagement at **P**reterm birth

CENTRAL – Cochrane Central Register of Controlled Trials

CINeMA – Confidence in Network meta-analysis

CPAP – continuous positive airway pressure

IPD – individual participant data

NICE – National Institute for Health and Care Excellence

For peer review only - http://bmjopen.bmj.com/site/about/guidelines.xhtml

NICU – neonatal intensive care unit

NIHR – National Institute for Health Research

NMA – network meta-analysis

PRISMA – Preferred Reporting Items for Systematic Review and Meta-Analysis

UK – United Kingdom

WHO – World Health Organization

ILCOR – International Liaison Committee on Resuscitation

PROSPERO – International prospective register of systematic reviews

For peer review only

For peer review only - http://bmjopen.bmj.com/site/about/guidelines.xhtml

iCOMP collaboration members:

Secretariat: Anna Lene Seidler[1], Lelia Duley[2], Alan Montgomery[2], Kylie E Hunter[1], Angie Barba[1], Ava Grace Tan-Koay[1], Lisa Askie[1]

Trialists: Amir Kugelman[3], Anu George[4], Anu Sachdeva[5], Anup Katheria[6], Arjan Te Pas[7], Ashish Kc[8], Bimlesh Kumar[9], Carl Backes[10], Catalina De Paco Matallana[11], Chamnan Tanprasertkul[12], Chayatat Ruangkit[13], Eugene Dempsey[14], G Ram Mohan[15], Guillermo Carroli[16], Heidi Al-Wassia[17], Hytham Atia[18], Heike Rabe[19], Islam Nour[20], Jiangqin Liu[21], John Bauer[22], John Kattwinkel[23], Judith Mercer[24], Justin Josephsen[25], Karen Fairchild[23], Kellie Murphy[26], Kristy Robledo[1], Lakhbir Dhaliwal[15], Laura Perretta[27], Lin Ling[28], Manoj Varanattu[29], Maria Goya[30], Musa Silahli[31], Neelam Kler[32], Neil Finer[33], Ola Andersson[8], Omar Kamlin[34], Peter Giannone[22], Pharuhad Pongmee[35], Prisana Panichkul[36], Sandeep Kadam[37], Sangkae Chamnanvanakij[36], Shigeharu Hosono[38], Shiraz Badurdeen[34], Thomas Ranjit[39], Venkataseshan Sundaram[15], Victor Lago Leal[40], Vikram Datta[41], Waldemar Carlo[42], Walid El-Naggar[43], William Tarnow-Mordi[1], William Oh[44]

Advisers: Graeme Polglase[45], John Simes[1], Martin Kluckow[46], Peter Davis[34], Stuart Hooper[45], Thomas P.A. Debray[47].

[1] National Health and Medical Research Council Clinical Trials Centre, University of Sydney, Sydney, Australia
[2] Nottingham Clinical Trials Unit, University of Nottingham, Nottingham, UK
[3] Bnai Zion Medical Center, Haifa, Israel
[4] Malankara Orthodox Syrian Church Medical College, Kerala, India
[5] All India Institute of Medical Science, New Delhi, India
[6] Sharp Healthcare, San Diego, California, USA
[7] Leiden University, Leiden, Netherlands
[8] Uppsala University, Uppsala, Sweden
[9] Lala Lajpat Rai Memorial Medical College, Uttar Pradash, India
[10] Ohio State University Wexner Medical Center, Columbus, Ohio, USA
[11] Clinic University Hospital Virgen de la Arrixaca, Murcia, Spain
[12] Thammasat University, Pathumthani, Thailand
[13] Mahidol University, Samut Prakan, Thailand
[14] Cork University Maternity Hospital, Cork, Ireland
[15] Post Graduate Institute of Medical Education & Research, Chandigarh, India
[16] Rosarino Center for Perinatal Studies, Rosario, Argentina
[17] King Abdulaziz University, Jeddah, Saudi Arabia
[18] Zagazig University, Zagazig, Egypt
[19] Brighton and Sussex Medical School, University of Sussex, Brighton, UK
[20] Mansoura University Children's Hospital, Mansoura, Egypt
[21] Tongji University School of Medicine, Shanghai, China
[22] University of Kentucky, Lexington, Kentucky, USA
[23] University of Virginia, Charlottesville, Virginia, USA
[24] The University of Rhode Island, Kingston, Rhode Island, USA
[25] St Louis University School of Medicine, St. Louis, Missouri, USA

For peer review only - http://bmjopen.bmj.com/site/about/guidelines.xhtml

[26] Mount Sinai Hospital, Toronto, Canada
[27] Weill Cornell Medical College, New York City, New York, USA
[28] Suining Central Hospital, Sichuan, China
[29] Jubilee Mission Medical College & Research Centre, Kerala, India
[30] Vall d'Hebron University Hospital, Barcelona, Spain
[31] Baskent University, Ankara, Turkey
[32] Sir Ganga Ram Hospital, New Delhi, India
[33] University of California, San Diego, California, USA
[34] The Royal Women's Hospital, Melbourne, Australia
[35] Ramathibodi Hospital, Mahidol University, Bangkok, Thailand
[36] Phramongkutklao Hospital, Ratchathewi, Bangkok, Thailand
[37] KEM Hospital, Pune, Maharashtra, India
[38] Jichi Medical University Saitama Medical Center, Saitama, Japan
[39] St John's Medical College & Hospital, Bangalore, Kamataka, India
[40] University Hospital of Getafe & European University of Madrid, Madrid, Spain
[41] Lady Hardinge Medical College, New Delhi, India
[42] University of Alabama at Birmingham, Birmingham, Alabama, USA
[43] Department of Pediatrics, Dalhousie University, Halifax, Canada
[44] Women and Infants' Hospital, Providence, Rhode Island, USA
[45] The Ritchie Centre, Obstetrics & Gynaecology, Monash University, Melbourne, Australia
[46] Faculty of Medicine and Health, University of Sydney, Sydney, Australia
[47] Julius Center for Health Sciences and Primary Care, University Medical Center Utrecht, Utrecht, the Netherlands

For peer review only

For peer review only - http://bmjopen.bmj.com/site/about/guidelines.xhtml

References

1. Blencowe H, Cousens S, Oestergaard MZ, Chou D, Moller AB, Narwal R, et al. National, regional, and worldwide estimates of preterm birth rates in the year 2010 with time trends since 1990 for selected countries: a systematic analysis and implications. Lancet. 2012;379(9832):2162-72.
2. March of Dimes P, Save the Children, WHO. Born too soon: the global action report on preterm birth. Geneva: World Health Organisation; 2012.
3. Chang HH, Larson J, Blencowe H, Spong CY, Howson CP, Cairns-Smith S, et al. Preventing preterm births: analysis of trends and potential reductions with interventions in 39 countries with very high human development index. Lancet. 2013;381(9862):223-34.
4. Petrou S, Mehta Z, Hockley C, Cook-Mozaffari P, Henderson J, Goldacre M. The impact of preterm birth on hospital inpatient admissions and costs during the first 5 years of life. Pediatrics. 2003;112(6 Pt 1):1290-7.
5. Mangham LJ, Petrou S, Doyle LW, Draper ES, Marlow N. The cost of preterm birth throughout childhood in England and Wales. Pediatrics. 2009;123(2):e312-27.
6. Zeitlin J, Draper ES, Kollee L, Milligan D, Boerch K, Agostino R, et al. Differences in rates and short-term outcome of live births before 32 weeks of gestation in Europe in 2003: results from the MOSAIC cohort. Pediatrics. 2008;121(4):e936-44.
7. Bhutta AT, Cleves MA, Casey PH, Cradock MM, Anand KJS. Cognitive and Behavioral Outcomes of School-Aged Children Who Were Born Preterm: A Meta-analysis. JAMA. 2002;288(6):728-37.
8. Aarnoudse-Moens CS, Weisglas-Kuperus N, van Goudoever JB, Oosterlaan J. Meta-analysis of neurobehavioral outcomes in very preterm and/or very low birth weight children. Pediatrics. 2009;124(2):717-28.
9. Anderson P, Doyle LW. Neurobehavioral outcomes of school-age children born extremely low birth weight or very preterm in the 1990s. JAMA. 2003;289(24):3264-72.
10. International Liaison Committee on Resuscitation (ILCOR). Neonatal Resuscitation- Part 7. 2015 International Consensus on Cardiopulmonary Resuscitation and Emergency Cardiovascular Care Science with Treatment Recommendations. In: Perlman JM, Wyllie J & Kattwinkel et al. Circulation. 2015;132(suppl 1):S204-S41.
11. Farrar D, Airey R, Law GR, Tuffnell D, Cattle B, Duley L. Measuring placental transfusion for term births: weighing babies with cord intact. BJOG. 2011;118(1):70-5.
12. Boere I, Roest A, E W, ten Harkel A, Haak M, Morley C, et al. Umbilical blood flow patterns directly after birth before delayed cord clamping. Arch Dis Child Fetal Neonatal Ed. 2015;100:F121–F125. doi:10.1136/archdischild-2014-307144.
13. Yao A, Moinian M, Lind J. Distribution of blood between infant and placenta after birth. The Lancet. 1969;294(7626):871-3.
14. Saigal S, O'Neill A, Surainder Y, Chua LB, Usher R. Placental transfusion and hyperbilirubinemia in the premature. Pediatrics. 1972;49(3):406-19.
15. Dawes GS. Foetal and neonatal physiology; a comparative study of the changes at birth [by] Geoffrey S. Dawes.   Chapter 13: Chicago, Year Book Medical Publishers; 1968. 247 p. illus. 27 cm. p.
16. Fogarty M, Osborn DA, Askie L, Seidler AL, Hunter K, Lui K, et al. Delayed vs early umbilical cord clamping for preterm infants: a systematic review and meta-analysis. American Journal of Obstetrics and Gynecology. 2018;218(1):1-18.
17. Vijayaselvi R, Abraham A, Kumar M, Kuruvilla A, Mathews J, Duley L. Measuring umbilical flow and placental transfusion for preterm births: weighing babies at 33-36 weeks gestation with cord intact. 1st congress of joint European Neonatal Societies. 2015.
18. Committee Opinion No. 684: Delayed Umbilical Cord Clamping After Birth. Obstetrics & Gynecology. 2017;129(1):e5-e10.
19. Schoonakker B, . Dorling, J,. Oddie, S,. Batra, D,. Grace, N,. Duley, L, editor Bedside resuscitation of preterm infants with cord intact is achievable using standard resuscitaire. Available at

For peer review only - http://bmjopen.bmj.com/site/about/guidelines.xhtml

p430 of
https://www.eiseverywhere.com/file_uploads/9206db9fe962868d47f709b38365ec5e_9349_abstract_book_-_25sett13-it-it.pdf. European Society for Pediatric Research; 2013; Oporto, Portugal.
20. Thomas M, Yoxall C, Weeks A, Duley L. Providing newborn resuscitation at the mother's bedside: assessing the safety, usability and acceptability of a mobile trolley. BMC Pediatrics. 2014;14:135.
21. Duley L, Dorling J, Pushpa-Rajah A, Oddie SJ, Yoxall CW, Schoonakker B, et al. Randomised trial of cord clamping and initial stabilisation at very preterm birth. Archives of Disease in Childhood - Fetal and Neonatal Edition. 2018;103(1):F6-F14.
22. Sawyer A, Ayers S, Bertullies S, Thomas M, Weeks AD, Yoxall CW, et al. Providing immediate neonatal care and resuscitation at birth beside the mother: parents' views, a qualitative study. BMJ Open. 2015;5(9).
23. Yoxall CW, Ayers S, Sawyer A, Bertullies S, Thomas M, D Weeks A, et al. Providing immediate neonatal care and resuscitation at birth beside the mother: clinicians' views, a qualitative study. BMJ Open. 2015;5(9).
24. Schoonakker B, . Dorling, J,. Oddie, S,. Batra, D,. Grace, N,. Duley, L. Bedside resuscitation of preterm infants with cord intact is achievable using standard resuscitaire. Available at p430 of https://www.eiseverywhere.com/file_uploads/9206db9fe962868d47f709b38365ec5e_9349_abstract_book_-_25sett13-it-it.pdf. European Society for Pediatric Research. 2013.
25. Kluckow M, Hooper SB. Using physiology to guide time to cord clamping. Seminars in Fetal and Neonatal Medicine. 2015;20(4):225-31.
26. Bhatt S, Alison BJ, Wallace EM, Crossley KJ, Gill AW, Kluckow M, et al. Delaying cord clamping until ventilation onset improves cardiovascular function at birth in preterm lambs. The Journal of physiology. 2013;591(8):2113-26.
27. Ersdal HL, Linde J, Mduma E, Auestad B, J P. Neonatal outcome following cord clamping after onset of spontaneous respiration. Pediatrics. 2014;134:265-72.
28. Nevill E, MP M. Effect of delayed cord clamping (DCC) on breathing and transition at birth in very preterm infants. Early Hum Dev 2015;91:407-11.
29. Polglase GR, Dawson JA, Kluckow M, Gill AW, Davis PG, te Pas AB, et al. Ventilation Onset Prior to Umbilical Cord Clamping (Physiological-Based Cord Clamping) Improves Systemic and Cerebral Oxygenation in Preterm Lambs. PLOS ONE. 2015;10(2):e0117504.
30. Polglase GR, Blank DA, Barton SK, Miller SL, Stojanovska V, Kluckow M, et al. Physiologically based cord clamping stabilises cardiac output and reduces cerebrovascular injury in asphyxiated near-term lambs. Arch Dis Child Fetal Neonatal Ed. 2017:https://doi.org/10.1136/archdischild-2017-313657 [Epub ahead of print].
31. Katheria AC, Brown MK, Faksh A, Hassen KO, Rich W, Lazarus D, et al. Delayed Cord Clamping in Newborns Born at Term at Risk for Resuscitation: A Feasibility Randomized Clinical Trial. The Journal of Pediatrics. 2017;187:313-7.e1.
32. Katheria A, Poeltler D, Durham J, Steen J, Rich W, Arnell K, et al. Neonatal Resuscitation with an Intact Cord: A Randomized Clinical Trial. Journal of Pediatrics. 2016;178:75-80.e3.
33. Hosono S, Mugishima H, Fujita H, Hosono A, Minato M, Okada T, et al. Umbilical cord milking reduces the need for red cell transfusions and improves neonatal adaptation in infants born at less than 29 weeks' gestation: a randomised controlled trial. Arch Dis Child Fetal Neonatal Ed. 2008;93(1):F14-9.
34. Blank DA, Polglase GR, Kluckow M, Gill AW, Crossley KJ, Moxham A, et al. Haemodynamic effects of umbilical cord milking in premature sheep during the neonatal transition. Archives of Disease in Childhood - Fetal and Neonatal Edition. 2017.
35. Katheria AC, Reister F, Hummler H, Essers J, Mendler M, Truong G, et al. LB 1: Premature Infants Receiving Cord Milking or Delayed Cord Clamping: A Randomized Controlled Non-inferiority Trial. American Journal of Obstetrics & Gynecology. 2019;220(1):S682.

For peer review only - http://bmjopen.bmj.com/site/about/guidelines.xhtml

36. WHO. WHO Recommendations for the Prevention and Treatment of Postpartum Haemorrhage. Geneva: World Health Organization. 2012.
37. National Institute for Health and Care Excellence. Preterm labour and birth. London; 2015.
38. European Resuscitation Council Guidelines for Resuscitation 2015 Section 7. Resuscitation and support of transition of babies at birth.
39. World Health Organisation. Guideline: delayed umbilical cord clamping for improved maternal and infant health and nutrition outcomes. Geneva, Switzerland; 2014.
40. Rabe H, Diaz-Rossello JL, Duley L, Dowswell T. Effect of timing of umbilical cord clamping and other strategies to influence placental transfusion at preterm birth on maternal and infant outcomes. Cochrane Database Syst Rev. 2012;8:CD003248.
41. Hosono S, Mugishima H, Fujita H, Hosono A, Minato M, Okada T, et al. Umbilical cord milking reduces the need for red cell transfusions and improves neonatal adaptation in infants born at less than 29 weeks' gestation: a randomised controlled trial. Arch Dis Child Fetal Neonatal Ed. 2008;93(1):F14-9.
42. Stewart LA, Tierney JF, Clarke M, on behalf of the Cochrane Individual Patient Data Meta-analysis Methods Group. Chapter 18: Reviews of individual patient data. In: Higgins J, Green S, editors. Cochrane Handbook for Systematic Reviews of Interventions: Version 5.1.0 [updated March 2011]. The Cochrane Collaboration, 2011. Available: www.cochrane-handbook.org.
43. Caldwell DM, Ades AE, Higgins JP. Simultaneous comparison of multiple treatments: combining direct and indirect evidence. Bmj. 2005;331(7521):897-900.
44. Lumley T. Network meta-analysis for indirect treatment comparisons. Statistics in medicine. 2002;21(16):2313-24.
45. Sutton A, Ades AE, Cooper N, Abrams K. Use of indirect and mixed treatment comparisons for technology assessment. PharmacoEconomics. 2008;26(9):753-67.
46. Debray TP, Schuit E, Efthimiou O, Reitsma JB, Ioannidis JP, Salanti G, et al. An overview of methods for network meta-analysis using individual participant data: when do benefits arise? Statistical methods in medical research. 2018;27(5):1351-64.
47. Ghersi D, Berlin J, Askie L. Chapter 19: Prospective meta-analysis. In: Higgins J, Green S, editors. Cochrane Handbook for Systematic Reviews of Interventions: Version 5.1.0 [updated March 2011]. The Cochrane Collaboration, 2011. Available: www.cochrane-handbook.org.
48. Higgins J, Deeks J, Altman D. Ch. 16.6: Indirect comparisons and multiple-treatments meta-analysis. Cochrane Handbook for Systematic Reviews of Interventions: Cochrane Collaboration; 2011.
49. Chaimani A, Caldwell DM, Li T, Higgins JP, Salanti G. Additional considerations are required when preparing a protocol for a systematic review with multiple interventions. Journal of clinical epidemiology. 2017;83:65-74.
50. Moher D, Shamseer L, Clarke M, Ghersi D, Liberati A, Petticrew M, et al. Preferred reporting items for systematic review and meta-analysis protocols (PRISMA-P) 2015 statement. Systematic reviews. 2015;4(1):1.
51. Stewart LA, Clarke M, Rovers M, Riley RD, Simmonds M, Stewart G, et al. Preferred Reporting Items for a Systematic Review and Meta-analysis of Individual Participant Data: The PRISMA-IPD StatementPRISMA Extension for Individual Patient DataPRISMA Extension for Individual Patient Data. JAMA. 2015;313(16):1657-65.
52. CPaCG. Detailed search methods used to maintain and update the Specialised Register [Available from: https://pregnancy.cochrane.org/sites/pregnancy.cochrane.org/files/public/uploads/Cochrane%20Pregnancy%20and%20Childbirth%20Group%20search%20methods%20for%20Intervention%20reviews_0.pdf last accessed July 31, 2018.
53. Higgins J, Green S. Cochrane handbook for systematic reviews of interventions. Oxford: Wiley-Blackwell; 2008.

For peer review only - http://bmjopen.bmj.com/site/about/guidelines.xhtml

54. Guyatt G, Oxman AD, Akl EA, Kunz R, Vist G, Brozek J, et al. GRADE guidelines: 1. Introduction—GRADE evidence profiles and summary of findings tables. Journal of clinical epidemiology. 2011;64(4):383-94.
55. Salanti G, Higgins JP, Ades A, Ioannidis JP. Evaluation of networks of randomized trials. Statistical Methods in Medical Research. 2008;17(3):279-301.
56. R Core Team. R: A language and environment for statistical computing. 2013.
57. Riley RD, Steyerberg EW. Meta-analysis of a binary outcome using individual participant data and aggregate data. Research Synthesis Methods. 2010;1(1):2-19.
58. Veroniki AA, Vasiliadis HS, Higgins JP, Salanti G. Evaluation of inconsistency in networks of interventions. International journal of epidemiology. 2013;42(1):332-45.
59. Peters JL, Sutton AJ, Jones DR, Abrams KR, Rushton L. Contour-enhanced meta-analysis funnel plots help distinguish publication bias from other causes of asymmetry. Journal of clinical epidemiology. 2008;61(10):991-6.
60. Schulz KF, Grimes DA. Multiplicity in randomised trials I: endpoints and treatments. Lancet (London, England). 2005;365(9470):1591-5.
61. Schulz KF, Grimes DA. Multiplicity in randomised trials II: subgroup and interim analyses. Lancet (London, England). 2005;365(9471):1657-61.

For peer review only

For peer review only - http://bmjopen.bmj.com/site/about/guidelines.xhtml

Figure headings

Figure 1. Network of possible comparisons between cord management interventions

Figure 2. Illustration of network of currently available trials comparing different timing of cord clamping.

For peer review only

For peer review only - http://bmjopen.bmj.com/site/about/guidelines.xhtml

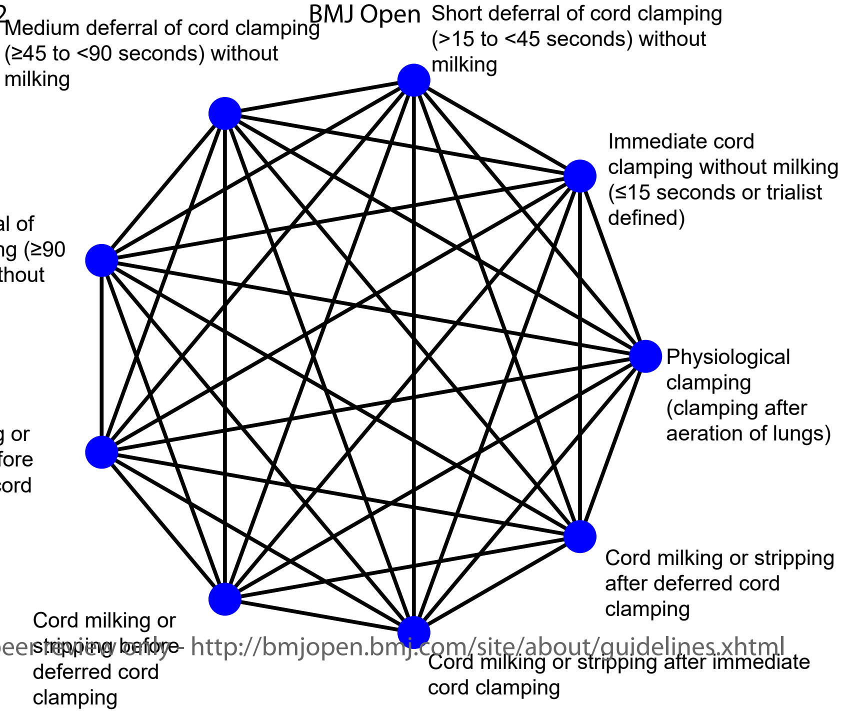

For peer review only - http://bmjopen.bmj.com/site/about/guidelines.xhtml

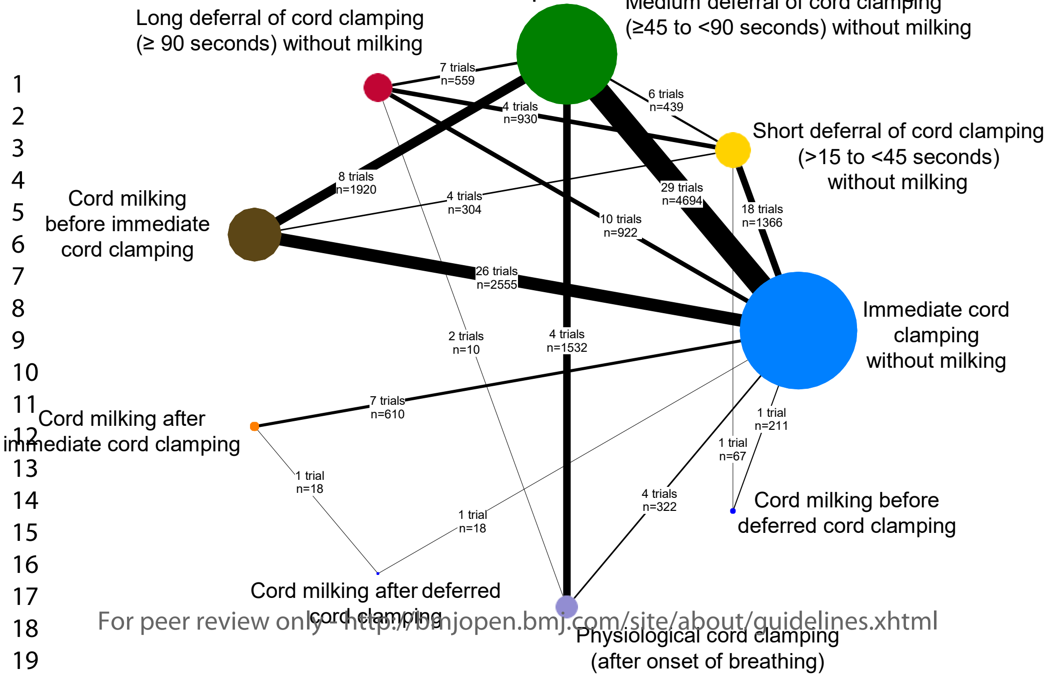

For peer review only - http://bmjopen.bmj.com/site/about/guidelines.xhtml

iCOMP Trial Master List September 2019

This table shows all trials that we have identified to date as being eligible for inclusion in iCOMP. All trials that have already agreed to provide their data are marked in blue.

Supplementary File 1: Eligible randomised trials to date for the pairwise & network meta-analysis with individual participant data on Cord Management at Preterm Birth (iCOMP)

Trial Country (PI)	Publication year	Start year/ completion year	Sample size	Participants	Intervention	Comparator	Primary outcome/s
Argentina [1] (Carroli)	n/a	2016/2020	700	Singletons, 24-30^6 weeks' GA	DCC: at 90 sec	DCC: ~30 sec	Sepsis (proven and very probable)
Australia [2] (Badurdeen)	n/a	2018/2020	120 (not all preterm)	≥32 weeks' GA*, require resuscitation at delivery	DCC: resuscitation prior to cord clamping (for PPV, clamping delayed until at least 60 sec after colour change of pedicap/neostat; for CPAP, clamping occurs at least 2 min after delivery)	ICC (followed by resuscitation)	Average heart rate between 60-120 sec after birth
Australia [3] (McDonnell)	1997	1994/1994	46	26 to 33 weeks' GA	DCC: 30 sec	ICC	Venous haematocrit
Australia [4] (Kamlin)	n/a	2014/2015	27 (not all preterm)	32-42 weeks' GA*	Arm 1: DCC at 90-180 sec<br>Arm 2: DCC 10 sec after crying and breathing established	DCC: <60 sec	Heart rate 90 sec after birth
Australia [5] (Tarnow-Mordi 2009)	n/a (Pilot for Tarnow-Mordi 2017)	2009/2010	100	<32 weeks' GA	Arm 1: Cord milking during resuscitation - cord cut long (3cm from placenta/ introitus)<br>Arm 2: DCC at 30-60 sec. If baby in extremis, immediate clamping.	ICC: within 10 sec	Haemoglobin 6 hours after birth

For peer review only - http://bmjopen.bmj.com/site/about/guidelines.xhtml

Trial Country (PI)	Publication year	Start year/ completion year	Sample size	Participants	Intervention	Comparator	Primary outcome/s
					Arm 3: DCC at 30-60 sec + milking		
Australia [6] (Tarnow-Mordi 2017)	2017	2010/2017	1634	<30 weeks' GA	DCC: ≥60 sec	ICC within 10 sec	Composite: Death or major morbidity (severe brain injury, severe ROP, NEC, or late onset sepsis) at 36 weeks' PMA
Austria [7] (Urlesberger)	n/a	2018/2021	80 (not all preterm)	>=28 weeks' GA*, caesarean	DCC: <30 sec, cord milking after long clamping at 30cm, 1x 10cm/sec	Standard care (cord cutting, no milking)	Changes in cerebral blood volume within 15 min after birth
Bangladesh [8] (Yasmeen)	2014	2012/2013	40	<37 weeks' GA	DCC: 3 minutes	DCC: 1 minute	Haemoglobin, iron and ferritin
Canada [9] (Chu/Murphy)	n/a	2007/2010	296	Singletons, 24-32 weeks' GA	DCC: at 30-45 sec	ICC	Composite: IVH or late onset sepsis
Canada [10] (El-Naggar)	2018	2011/2014	73	24-30^6 weeks' GA	UCM: x3, at or below the level of the placenta, ~20 cm milked, before clamping	ICC	Systemic blood flow (Superior vena cava flow at 4-6 hours after birth)
Canada [11] (Saigal)**	1972	n/a	125 (preterm)	Premature infants 28-36 weeks GA and weighing 1020g-3250g OR full-term infants 28-42 weeks GA and weighing 2685g-4350g*	Arm 1: DCC at 1min<br>Arm 2: DCC at 5min	ICC	RBC volume, blood volume, haematocrit, plasma volume
China [12] (Dai)	2014	n/a	52 (preterm)	Singletons, term and preterm infants*	DCC: Wait until cord pulsation ceased	ICC: 5-10 sec	RBC count (72-96 hrs after birth), Anaemia (2 wks), clinically significant pathological polycythaemia, white blood cell count (72-96 hrs after birth), fetal bilirubin from birth to day 5, jaundice within 24 hrs of birth, Apgar

For peer review only - http://bmjopen.bmj.com/site/about/guidelines.xhtml

iCOMP

Trial Country (PI)	Publication year	Start year/ completion year	Sample size	Participants	Intervention	Comparator	Primary outcome/s
							(1 min, 5 min), respiratory distress, rectal temperature 5 min after birth, neonate well-being at 1 month
China [13] (Dong)	2016	2015/2015	90	Singletons, <32 weeks' GA	DCC: 45 sec	ICC: <10 sec	Severe IVH – grades 3 and 4
China [14] (Hao)	n/a	2018/2019	48	30-31^6 weeks' GA	UCM	DCC	Cerebral haemodynamics 15 min after birth
China [15] (Hu)	2015 (master's thesis)	n/a	120	28-35 weeks' GA, vaginal birth	Arm 1: DCC at 30 sec<br>Arm 2: DCC at 60 sec<br>Arm 3: DCC at 120 sec	ICC < 10 sec	Haematocrit and haemoglobin levels at 24 hrs and 1 wk after birth
China [16] (Hua)	2010?	2009/2011	176 (49 of those preterm)	Any GA*	Normal birth<br>Arm 1: DCC – wait until cord ceases pulsing<br>Arm 2: DCC – at 90 sec<br>Asphyxia<br>Arm 1: DCC – wait until cord ceases pulsing, resuscitate on bed site with cord intact	Normal birth<br>ICC <10 sec<br>Asphyxia<br>ICC <10 sec, resuscitate after on irradiation table	Haemoglobin 1 month after birth
China [17] (Li)	2018	2017/2017	102	delivered vaginally between 28^0-36^6 weeks', and premature prolonged rupture of membranes	UCM: x4 at a speed of 10cm/sec, then clamped	ICC	Incidence of certain or probable infection in neonates
China [18] (Liu)	n/a	2019/2019	948 (not all preterm)	34^0-38^6 weeks' GA*, caesarean section	DCC: 60 sec	ICC: within 10 sec	Rate of respiratory distress within 24 hours after birth
China [19] (Shi)	2017	n/a	60 preterm (and 460 term)	Single foetus deliveries*	DCC	ICC: 5-10 sec	Hemoglobin (newborn cord blood & after 24 hrs), neonatal complications, bleeding volume, third labour

For peer review only - http://bmjopen.bmj.com/site/about/guidelines.xhtml

Trial Country (PI)	Publication year	Start year/ completion year	Sample size	Participants	Intervention	Comparator	Primary outcome/s
							time, incidence of placental adhesion and peeling
China [20] (Xie)	n/a	2017/2019	300	Singletons, <34 weeks' GA	UCM: x2-3, 25cm/2 sec, below placenta level, before clamping	ICC	Haemoglobin, Haematocrit, and ferritin level at 48 hours
Egypt [21] (Allam)	n/a	2018/2019	210	Singletons, 30-34 weeks' GA	DCC: until cord stops pulsing or 1-2 min	ICC: <5 sec	Fetal haemoglobin, bilirubin, death
Egypt [22] (Nour 2017a)	n/a	2017/2019	90	<34 weeks' GA	UCM: x3 at 10cm/sec, below placenta level, cord held 20-25cm from baby	ICC	Peripheral venous CD34 at admission
Egypt [23] (Nour 2017b)	n/a	2017/2018	90	<34 weeks' GA	Arm 1: ICC, with placental insufficiency<br>Arm 2: DCC at 60sec, with placental insufficiency	Normal placenta with DCC at 60 sec	Peripheral venous CD34 at admission
Germany [24] (Nelle)	1998	n/a	19	PT <1500g*, born by caesarean section	DCC: 30 sec, 30 cm below placenta	ICC	Mean Blood Pressure, left ventricular output, mean cerebral blood flow velocity in the arteria carotis interna, haemoglobin, haematocrit , systemic and cerebral haemoglobin transport, systemic vascular resistance
Germany [25] (Rabe 2000)	2000	2006/2008	40	<33 weeks' GA	DCC: 45 sec	DCC: 20 sec	Feasibility, effects on post-partal adaption and anaemia of prematurity
India [26] (Aghai 2018)	n/a	2018/2020	1400 (not all preterm)	Depressed neonates, 35-42 weeks' GA*	UCM: x4, 30cm over 2 sec	ICC: immediately after birth	Number of infants with moderate to severe HIE or death
India [27] (Anusha)	n/a	2017/2019	148	birth weight <1500g*	DCC: 30 sec	ICC: within 10 sec	Haemodynamic stability, haematological status, serum ferritin, and requirement of

For peer review only - http://bmjopen.bmj.com/site/about/guidelines.xhtml

iCOMP

Trial Country (PI)	Publication year	Start year/ completion year	Sample size	Participants	Intervention	Comparator	Primary outcome/s
							blood transfusion between birth and 6 months of age
India [28] (Bhriguvanshi)	n/a	2017/2018	236	> 28 weeks' GA, requiring resuscitation*	UCM: x3 towards baby at 10cm/sec, then clamped	ICC: within 30 sec	Haemoglobin and haematocrit at birth and 6 weeks of age
India [29] (Chopra)	2018	2013/2015	142	growth retarded babies (IUGR) ≥ 35 weeks' GA*	DCC: 60 sec	ICC: 10 sec	Haemoglobin and ferritin levels
India [30] (Das/Sundaram)	2018	2012/2013	461	30-33^6 weeks' GA	DCC: 60 sec below placenta. If baby depressed, immediate clamping keeping cord long, milked x2-3 during resuscitation	ICC: within 10 sec	Composite of mortality or abnormal neurological examination at 40 weeks PMA
India [31] (Datta)	2017	2011/2013	120	Singletons, 34-36^6 weeks' GA	DCC: at >30-<60 sec	ICC: <20 sec	Neurobehavioural Assessment of Preterm Infant at 37 weeks' post-conceptional age
India [32] (Dhaliwal)	2014	n/a	300	34-37 weeks' GA	DCC: 60 sec	ICC: <10 sec	Risk of neonatal mortality & abnormal neurological examination at 40 weeks' GA
India [33] (Dipak)	2017	2012/2013	78	27-31^6 weeks' GA	Arm 1: DCC: 60 sec Arm 2: DCC: 60 sec with intramuscular ergometrine	ICC: <10 sec	Hematocrit 4 h after birth
India [34] (George/Isac)	n/a	2017/2018	180 (not all preterm)	Mothers at 34-40^6 weeks' GA*	UCM: milking whole length at 10cm/sec x3, then clamped	ICC	Infant haemoglobin and haematocrit at 72hrs and 6 weeks
India [35] (Gupta)	n/a	2018/2020	110	<34 weeks' GA	DCC: 30 sec	ICC	Ferritin and PCV at 8 weeks
India [36] (Kumar)	2015	2013/2014	200	32-36^6 weeks' GA, vaginal or caesarean	UCM: x3, 10cm/sec	ICC	Haemoglobin and ferritin at 1.5 months

For peer review only - http://bmjopen.bmj.com/site/about/guidelines.xhtml

iCOMP

**Trial Country (PI)**	**Publication year**	**Start year/ completion year**	**Sample size**	**Participants**	**Intervention**	**Comparator**	**Primary outcome/s**
India [37] (Ram Mohan)	2018	2015/2016	60	<37 weeks' GA, requiring resuscitation	UCM: 20-25 cm umbilical cord x3 at 10cm/sec within 30 sec of birth	No milking	Haemoglobin and serum ferritin at 6 weeks
India [38] (Rana/Agarwal)	2018	2013/2013	100	<34 weeks' GA	DCC: 120 sec	ICC: ≤30 sec	serum total bilirubin and haematocrit levels at 48 hrs and 7 days
India [39] (Ranjit)	2015	2010/2010	100	30-36[6] weeks' GA	DCC: >2min	ICC	Haematocrit and serum ferritin at 6 weeks
India [40] (Kumar Mangla/ Thukral)	n/a	2016/2017	144 (not all preterm)	Late preterm and term neonates*	Deferred UCM: cord clamped at 60 sec	UCM: Cord milking in 10 sec	Venous haematocrit at 48 hours of life
India [41] (Upadhyay 2010)	2013	2010/2011	170 (not all preterm)	>35 weeks' GA*	UCM: x3 at 10cm/sec, then clamped at ~25 cm of length within 30 sec of birth	ICC: <30 sec	Haemoglobin and serum ferritin at 6 weeks
India [42] (Varanattu)	n/a	2018/2019	250	<32 weeks' GA	UCM: x3 over 20 sec at 20cm/2sec with 2 second pause between	ICC: clamped immediately	Haemoglobin levels at birth and IVH (incidence and severity) at 7 days
Iran [43] (Armanian)	2017	2014/2015	63	≤34 weeks' GA	DCC: at 30-45 sec	ICC: at 5-10 sec	Time of cord clamping
Iran [44] (Mojaveri)	2017	2014/2015	70	<32 weeks' GA, caesarean, birth weight < 1500g, not requiring advanced resuscitation	DCC: at 30-45 sec	ICC: <10 sec	IVH (days 3 to 7), survival infant (up to 28 days)
Iran [45] (Mirzaeian)	n/a	2017/2018	160	28-34 weeks' GA	UCM: milked x3 in 10 sec	ICC	Amount of transfused blood, bilirubin levels
Iran [46] (Sekhavat)	2008	n/a	52	26-34 weeks' GA	DCC: 30-60 sec	ICC: 10-15 sec	Blood pressure, haematocrit, blood glucose

For peer review only - http://bmjopen.bmj.com/site/about/guidelines.xhtml

iCOMP

Trial Country (PI)	Publication year	Start year/ completion year	Sample size	Participants	Intervention	Comparator	Primary outcome/s
Iran [47] (Shahgheibi)	n/a	2017/2018	90	Women with preterm labour	DCC: 180 sec	DCC: 30 sec	blood parameters, weaning from ventilator, NICU discharge time
Ireland [48] (Dempsey)	n/a	2015/2016	45	<32 weeks' GA	Arm 1: DCC at 60 sec on mobile resuscitation trolley at/below placenta level<br>Arm 2: UCM – Cord stripped 3 times at 20cm/2 sec at/below placenta level	ICC: <20 sec	Neonatal: Brain activity (6 & 12 hours post-partum, EEG and NIRS)<br>Maternal: hemoglobin at 24-36 hours post-partum
Israel [49] (Kugelman)	2007	2004/2005	65	24-35[6] weeks' GA	DCC: 30-45 sec, below placenta level	ICC: <10 sec	Haematocrit, blood pressure
Japan [50] (Hosono 2008)	2008	2001/2002	40	24-28 weeks' GA, singletons	UCM: 20 cm of the cord, x2-3, before clamping, 20cm/2sec	ICC	Probability of not needing transfusion, number of RBC transfusions
Japan [51] (Hosono 2016)	2016	2008/2016	203	24-27[6] weeks' GA	UCM: cord cut 30 cm from infant, cord milked x1	ICC: <30 sec	1) Probability needing transfusion and death<br>2) Amount of blood transfusion first 4 weeks
Korea [52] (Song)	2017	2012/2015	66	24-32[6] weeks' GA	UCM: x4 at 20cm/2sec, with 2 sec pause between	ICC: immediately after delivery	Short term safety: Apgar score, prevalence of hypothermia, early intubation, initial blood gas analyses, bilirubin levels, duration of phototherapy, use of cross-transfusion, respiratory distress.
Macedonia [53] (Zisovska)	2008	n/a	57	Premature newborns	DCC: 1 min	ICC	Hematological parameters, number of RBC transfusions
Nepal [54] (Andersson)	2017	2014/2017	540 (not all preterm)	34-41 weeks' GA*	DCC: at ≤180 sec	ICC: ≤30 sec	Haemoglobin at 8±1 months

For peer review only - http://bmjopen.bmj.com/site/about/guidelines.xhtml

Trial Country (PI)	Publication year	Start year/ completion year	Sample size	Participants	Intervention	Comparator	Primary outcome/s
Nepal [55] (Ashish KC)	n/a	2016/2016	1510 (not all preterm)	Singletons, normal vaginal delivery, ≥33 weeks GA*	DCC: at 180 sec	DCC: <60 sec	Neonatal heart rate continuously until 10 min after birth and at 1,3&5 min
Netherlands [56] (Te Pas)	n/a	2019/2020	660	<30 weeks' GA	Physiology-based cord clamping (PBCC): Resuscitation with cord intact, clamp when infant is stable (heart rate >100 bpm, oxygen>80%, supplemental oxygen <40%)	ICC/DCC: immediately or delayed 30-60 sec, depending on clinical condition of infant	Intact survival at NICU discharge (without cerebral injury (IVH ≥ grade 2 and/or PVL ≥ grade 2 and/or periventricular venous infarction) and/or NEC (Bell stage ≥ 2)
Netherlands [57] (Ultee)	2008	n/a	37	34 to 36^6 weeks' GA	DCC: 3 min	ICC: <30 sec	blood glucose levels at 1,2, and 3hrs of age, haemoglobin and haematocrit at 1hr and 10 weeks, ferritin at 10 weeks
Pakistan [58] (Malik)	2013	2009-2009	80	30-37 weeks' GA	DCC: 120 sec	DCC: 30 sec	Haematocrit
Saudi Arabia [59] (Al-Wassia)	n/a	2017/2019	180	<32 weeks' GA	UCM: milked 20cm segment over 2-3 sec x3	DCC: 60 sec	IVH at 28 days
Saudi Arabia [60] (Gomaa)	n/a	2016/2018	200	24 to 34^6 weeks' GA	DCC: 45-60 sec, baby at level or below placenta	UCM: milked x4-5 from maternal end of cord to baby abdomen, 2 sec pause between milking	Haematological parameters - haematocrit
South Africa [61] (Hofmeyr 1988)	1988	n/a	38	Singleton, <35 weeks' GA	Arm 1: DCC at 60sec<br>Arm 2: DCC at 60sec + ergometrine	ICC	PVH/IVH at 6-72hrs after birth, Apgar score at 5min, birthweight, systolic blood pressure at 5min, cord blood gas and death.
South Africa [62] (Hofmeyr 1993)	1993	n/a	86	<2000 g birthweight*	DCC: 1-2 min	ICC	death of the baby, PVH/IVH at 6-72hrs after birth, Apgar

For peer review only - http://bmjopen.bmj.com/site/about/guidelines.xhtml

Trial Country (PI)	Publication year	Start year/ completion year	Sample size	Participants	Intervention	Comparator	Primary outcome/s
							score at 5min, cord-pH, bilirubin
South Africa [63] (Tiemersma)	2015	2012/2012	104 (not all preterm)	Birth weight <2500g ± 500g*	DCC: 2-3 minutes	ICC: within 30 sec	Haemoglobin from cord blood and at 2 months
Spain [64] (De Paco Matallana)	n/a	2011/2014	100	24- 34 weeks' GA	DCC: 45-60 sec	ICC: <10 sec	Neonatal haemoglobin, haematocrit and bilirubin levels (within 7 days after birth)
Spain [65] (Domingo Puiggrós)	n/a	2014/2016	40	<34 weeks' GA, caesarean	UCM: x3 at 20 cm/2sec	DCC: 30 sec	Haemoglobin at 1 and 24 hrs
Spain [66] (Leal)	2019	2013/2016	138	24^0-36^6 weeks' GA	UCM: nearly 20cm cord milked towards umbilicus x4 before clamping	ICC: <20 sec	Requirement of RBC transfusions or phototherapy
Spain [67] (Socias)	n/a	2014/2017	150	26-32^6 weeks' GA	DCC: 30-60 sec	ICC: <30sec	RBC transfusions (number & volume), IVH, postpartum haemorrhage
Switzerland [68] (Baenziger)	2007	1996/1997	39	24-32 weeks' GA	DCC: 60-90 sec, below placenta, syntocinon	ICC: <20 sec	Cerebral oxygenation at 4, 24 and 72 hrs of age
Taiwan [69] (Shen)	n/a	2015/2019	100	<30 weeks' GA	UCM: milked once after ICC, 20cm section at speed of 10cm/sec and clamped at 2-3cm.	ICC and no milking	Neonate's haemoglobin, haematocrit, and mean arterial pressure at admission
Thailand [70] (Chamnanvanakij)	2017	2015/2016	46	25-34 weeks' GA	UCM: x3-4, 30 cm, before clamping	DCC: at 60 sec	Haematocrit level 2 hrs after birth
Thailand [71] (Jomjak)	n/a	2018/2018	110	Singleton, 24-36^6 weeks' GA	DCC	ICC	Haematocrit at 2 and 48 hrs
Thailand [72] (Mungkornkaew)	2015	2014/2014	200 (not all preterm)	Singleton, 34-42 weeks' GA*, vaginal delivery	DCC: 2 minutes	DCC: 1 minute	Fetal haematocrit

For peer review only - http://bmjopen.bmj.com/site/about/guidelines.xhtml

Trial Country (PI)	Publication year	Start year/ completion year	Sample size	Participants	Intervention	Comparator	Primary outcome/s
Thailand [73] (Panichkul)	n/a	2015/2016	70	34-36 weeks' GA	DCC: at 60 sec	ICC: at 10 sec	Haematocrit 2 hours after birth
Thailand [74] (Ruangkit)	2019	2016/2017	47	Multiples, 28-36 weeks' GA	DCC: at 30-60 sec	ICC: <10 sec	Haematocrit level at birth
Thailand [75] (Salae)	2016	2014/2015	86	34-36^{6} weeks' GA	DCC: at 2 minutes	ICC: within 30 sec	Haematocrit at 48 hours
Thailand [76] (Tanthawat)	n/a	2016/2016	40	<32 weeks' GA	UCM: Cut cord at 30cm, cord milking x1 at 10cm/sec, clamp and cut cord at 1-2cm from umbilical stump	ICC: <10 sec	Haemoglobin and haematocrit level at admission
Turkey [77] (Alan)	2014	2011/2013	44	≤32 weeks' GA ≤1500 g	UCM: at 25-30 cm x3 at 5cm/s before clamping	ICC: <10 sec	Number and volume of packed RBC transfusions received by infant during first 35 days of life
Turkey [78] (Gokmen)	2011	2008/2009	42	24-31^{6} weeks' GA	DCC: 30-45 sec	ICC: 5-10 sec	peripheral blood hematopoietic progenitor cells before any blood product administered to infants
Turkey [79] (Kilicdag)	2015	2012/2013	54	≤32 weeks' GA	UCM: x4 before clamping (20cm/2sec)	ICC	absolute neutrophil counts
Turkey [80] (Silahli)	2018	2015/2016	75	≤32 weeks' GA	UCM: at 20 cm x3, before clamping	ICC: <10 sec	Thymic size
UK [81] (Aladangady)	2006	n/a	46	24-32^{6} weeks' GA	DCC: 30-90 sec, below placenta, oxytocic agent, with ventilation/ resuscitation if necessary	ICC	Infants' blood volume
UK [82] (Duley)	2018	2013/2015	261	<32 weeks' GA	DCC: after at least 2 min	ICC: <20 sec	Death before hospital discharge, intraventricular haemorrhage

For peer review only - http://bmjopen.bmj.com/site/about/guidelines.xhtml

iCOMP

Trial Country (PI)	Publication year	Start year/ completion year	Sample size	Participants	Intervention	Comparator	Primary outcome/s
UK (Holland)	Not published	1998/2001	?	<33 weeks' gestation	DCC: 40-90 s	ICC (?)	Median arterial/alveolar PO2 ratio over the first 24 hrs of life
UK [83] (Kinmond)	1993	n/a	36	>27 & <32 weeks' GA, vaginal delivery	DCC: 30 sec, 20 cm below placenta	ICC: 10 sec median	Initial packed cell volume, peak serum bilirubin concentrations, red cell transfusions, respiratory impairment
England [84] (Medina)	2013	n/a	51	24-31 weeks' GA	DCC	ICC	Haemodynamic parameters, included vena cava blood flow, ventricular outflow, and flow velocity.
UK [85] (Rabe)	2011	2006/2008	58	Singleton, <34 weeks' GA	UCM: x4	DCC: at 30 sec	neonatal blood haematocrit and haemoglobin at 1 hr after birth
USA [86] (Backes)	2016	2009/2013	40	Singletons, 22^{5}-27^{6} weeks' GA	DCC: 30-45 sec, below placenta	ICC: <10 sec	Safety, feasibility, haematological and circulatory outcomes
USA [87] (Bauer)	n/a	2014/2019	300	>24 and <30 weeks' GA	Arm 1: DCC at 45 sec and indomethacin within 6 hrs<br>Arm 2: DCC at 45 sec and placebo within 6 hrs	Arm 3: ICC and indomethacin<br>Arm 4: ICC and placebo	Fraction of survivors with no severe IVH (grades 3 or 4) or PVL within first 60 days of life
USA [88] (Berens)	n/a	2018/2019	100 (not all preterm)	≥35 weeks GA*, at least 1 previous child that received phototherapy for hyperbilirubinemia	DCC: 60 sec	ICC: <15 sec	Neonatal bilirubin level 24 hours after birth
USA [89] (Bienstock)	n/a	2011/2013	22	24^{0}-32^{6} weeks' GA	UCM: x4 over 10 min	ICC	Haemoglobin within 24 hours of birth and through NICU stay

For peer review only - http://bmjopen.bmj.com/site/about/guidelines.xhtml

iCOMP

Trial Country (PI)	Publication year	Start year/ completion year	Sample size	Participants	Intervention	Comparator	Primary outcome/s
USA [90] (March/deVeciana)	2013	2009/2011	113	Singletons, 24-28^{6} weeks' GA	UCM: 10cm, immediately after delivery, ~20cm actively milked x 3	ICC	RBC transfusion within 28 days of life
USA [91] (Driggers)	n/a	2011/2013	2	Infants delivered at 24^{0} to 28^{6} weeks' GA	Arm 1: DCC at 30 sec Arm 2: UCM x4 in 10 sec	Arm 3: ICC	Adverse neonatal event: composite of BPD, NEC, grade 3 or 4 IVH or PVL, or death prior to discharge
USA [92] (Elimian)	2014	2008/2011	200	Singletons, 24-34 weeks' GA	DCC: at 30-35 sec (3-4 passes of milking toward the neonate was allowed)	ICC: <5 sec	Need for blood transfusion
USA [93] (Ibrahim)	2000	n/a	32	Birthweight 501g-1250g, 24 to <29 weeks' GA	DCC: 20 sec	ICC	Number of blood transfusions
USA [94] (Josephsen)	n/a	2012/2016	80	24-27^{6} weeks' GA	UCM: below level of placenta and ~20 cm cord milked x3 over 10-20 sec before clamping	ICC	Haemoglobin and haematocrit concentrations (within 4 hrs birth) Incidence and number blood transfusions until discharge
USA [95] (Katheria 2011)	2014 & 2017	2011/2013	60	<32 weeks' GA	UCM: x3, below placenta, about 20cm of cord over 2 sec	ICC	Superior vena cava flow at 6 hours
USA [96] (Katheria 2013)	2015 & 2017	2013/2018	197	23-31^{6} weeks' GA	UCM: x4 at 20 cm/2 sec	DCC: at 45-60 sec	Superior vena cava flow at <12 hrs
USA [97] (Katheria 2016)	2016	2014/2015	150	<32 weeks' GA	CPAP + DCC at 60s	DCC: 60s	Peak haematocrit in first 24 hours of life
USA [98] (Katheria 2017)	n/a	2017/2022	1200	23-32^{6} weeks' GA	UCM: x4 at 20cm/2 sec	DCC: at least 60 sec	Incidence of IVH or death at discharge, up to 6 months corrected gestational age

For peer review only - http://bmjopen.bmj.com/site/about/guidelines.xhtml

Trial Country (PI)	Publication year	Start year/ completion year	Sample size	Participants	Intervention	Comparator	Primary outcome/s
USA [99] (Katheria 2019)	n/a	2019/2020	1000 (not all preterm)	Non-vigorous newborns born at 35-42 weeks' GA*	UCM: x4, entire umbilical length over 2 sec.	ICC: within 30 sec	Admission to NICU in the first 48 hrs of life
USA [100] (Kattwinkel)	n/a	2016/2021	940	23^0-28^6 weeks' GA	DCC: Assisted ventilation (face mask, CPAP or PPV) prior to DCC at 120 sec	DCC: 30-60 sec, assisted ventilation only after cord clamping	IVH (7-10 days)
USA [101] (Krueger)	2015	2012/2013	67	Singletons, 22-31^6 weeks' GA	DCC & UCM: cord milking x4 with 4-5 sec between each, then DCC at 30 sec	DCC: 30 sec, without cord milking	Initial fetal haematocrit
USA [102] (Martin)	n/a	2012/2014	72	Singletons, 23-37 weeks' GA	Arm 1: DCC at 60 sec<br>Arm 2: DCC at 40 sec	DCC: 20 sec	IVH number and severity (15 months)
USA [103] (Mercer 2003)	2003	1998/2001	32	Singletons, 24-31^6 weeks' GA	DCC: 30-45 sec	ICC: 5-10 sec	Initial mean blood pressure on arrival in NICU
USA [104] (Mercer 2006)	2006	2003/2004	72	Singletons, <32 weeks' GA	DCC: 30-45 sec	ICC: 5-10 sec	BPD, suspected NEC
USA [105] (Mercer 2008)	2011 & 2016 & 2018	2008/2014	211	Singletons, 24-31^6 weeks' GA	DCC & UCM: milking x1 then DCC at 30-45 sec. If clamping cannot be deferred, cord milked x2-3 quickly	ICC: <10 sec	IVH, late onset sepsis
USA [106] (Oh)	2011	2000/2001	33	Singletons, 24-27^6 weeks' GA	DCC: at 30-45 sec	ICC: <10 sec	venous haematocrit at 4 hours of age
USA [107] (Perlman)	n/a	2015/2019	150	28-34^6 weeks' GA	DCC: at 60 sec	DCC: at 30 sec	Haematocrit 1 hour after birth
USA [108] (Smith)	n/a	2014/2018	282	23^0-34^6 weeks' GA	UCM: x4, before clamping	DCC: at 30 sec	Haemoglobin & haematocrit in NICU from admission to discharge
USA [109] (Strauss)	2008	n/a	158[97]	≤36 weeks' GA	DCC: 60 sec	ICC	RBC volume/mass, per biotin labelling

For peer review only - http://bmjopen.bmj.com/site/about/guidelines.xhtml

Trial Country (PI)	Publication year	Start year/ completion year	Sample size	Participants	Intervention	Comparator	Primary outcome/s
USA [110] (Yared/Young)	n/a	2015/2016	39	Very low birth weight (500 to 1500 grams)*	DCC: at 60 sec	DCC: at 30 sec	IVH (during NICU admission up to 6 months)
Thailand [111] (Pongmee)	2010 (abstract)		43	<35 weeks' GA	UCM: x2 along 30 cm after cutting	ICC	Initial haematocrit, need for blood transfusion

* only those born <37 weeks gestation eligible

** PI advised individual participant data not available due to time elapsed since trial

PI = Principal Investigator
NICU = neonatal intensive care unit
DCC = deferred cord clamping
CPAP = continuous positive airway pressure
PVL = periventricular leukomalacia
IVH = intraventricular haemorrhage
NEC = necrotising enterocolitis
cm = centimetres
GA = gestational age
UCM = umbilical cord milking
PO2 = partial pressure of oxygen
ROP = retinopathy of prematurity
PVH = periventricular haemorrhage
IUGR = intrauterine growth retardation
sec = seconds
PMA = postmenstrual age
PBCC = physiological based cord clamping
PPV = positive pressure ventilation
BPD = bronchopulmonary dysplasia
HIE = hypoxic ischemic encephalopathy
PCV = polycythaemia
min = minutes
ICC = immediate cord clamping
RBC = red blood cell
NIRS = near-infrared spectroscopy
EEG = electroencephalogram

References

1. ISRCTN registry website: **Early compared to delayed umbilical cord clamping in very small prematurely born babies: A study to know which one is better for infant health. http://www.isrctn.com/ISRCTN12219110. Accessed August 27, 2018**.
2. ANZCTR registry website: **Resuscitating newborn infants with the umbilical cord intact- The Baby-Directed Umbilical Cord Cutting (Baby-DUCC) Trial. http://www.anzctr.org.au/ACTRN12618000621213.aspx. Accessed 20th February, 2019.**
3. McDonnell M, Henderson-Smart D: **Delayed umbilical cord clamping in preterm infants: a feasibility study**. *Journal of paediatrics and child health* 1997, **33**(4):308-310.
4. ANZCTR website: **CORD CLAMPING STUDY: Early versus delayed cord clamping and its effects on infant heart rate and oxygen saturation in the first minutes after birth. https://www.anzctr.org.au/Trial/Registration/TrialReview.aspx?id=365659. Access 20th February, 2019**.
5. Australian New Zealand Clinical Trials Registry: **Australian Placental Transfusion Pilot Study: investigating standard cord clamping procedures versus three methods of autologous placental blood transfusion in pre term infants. https://www.anzctr.org.au/Trial/Registration/TrialReview.aspx?ACTRN=12609000248268. Accessed February 20, 2019**.
6. Tarnow-Mordi W, Morris J, Kirby A, Robledo K, Askie L, Brown R, Evans N, Finlayson S, Fogarty M, Gebski V *et al*: **Delayed versus Immediate Cord Clamping in Preterm Infants**. *New England Journal of Medicine* 2017, **377**(25):2445-2455.

For peer review only - http://bmjopen.bmj.com/site/about/guidelines.xhtml

iCOMP

7. ClinicalTrials.gov registry: **C-UCM and Cerebral Oxygenation and Perfusion. Available at https://clinicaltrials.gov/ct2/show/NCT03748914. Accessed February 20, 2019.**
8. Yasmeen S, Shahidullah M, Mannan M, Dey A, Chowdhury R, Haque A, Hassan MZ: **Iron Status in Early versus Delayed Cord Clamping Groups of Preterm Neonates Delivered in a Tertiary Level Hospital**. *Journal of Armed Forces Medical College, Bangladesh* 2015, **10**(2):62-65.
9. Chu K, Whittle W, Windrim R, Shah P, Murphy K: **The DUC trial: A pilot randomized controlled trial of immediate vs. delayed umbilical cord clamping in preterm infants born between 24 and 32 weeks gestation**. *American Journal of Obstetrics and Gynecology* 2011, **204**(1 Suppl):S201.
10. El-Naggar W, Simpson D, Hussain A, Armson A, Dodds L, Warren A, Whyte R, McMillan D: **Cord milking versus immediate clamping in preterm infants: a randomised controlled trial**. *Archives of Disease in Childhood - Fetal and Neonatal Edition* 2019, **104**(2):F145-F150.
11. Saigal S, O'Neill A, Surainder Y, Chua LB, Usher R: **Placental transfusion and hyperbilirubinemia in the premature**. *Pediatrics* 1972, **49**(3):406-419.
12. Dai S, Jin X, Qi HJ: **Effect of different cord clamping times on the prognosis of newborns (不同断脐时间对新生儿预后的影响)**. *Modern Pract Med* 2014, **26**:69-71.
13. Dong XY, Sun XF, Li MM, Yu ZB, Han SP: **Influence of delayed cord clamping on preterm infants with a gestational age of <32 weeks [in Chinese]**. *Zhongguo Dang Dai Er Ke Za Zhi* 2016, **18**:635-638.
14. Chinese Clinical Trial Registry website: **Effect of delayed cord clamping versus unbilical cod milking on cebral blood flow in preterm infant: a randomized, double-blind controlled trial. http://www.chictr.org.cn/showprojen.aspx?proj=30981. Accessed February 20, 2019.**
15. Hu X, Xu X: **The Effects of Different Cord Clamping Time in Preterm Infants by Vaginal Delivery [Master's Degree Thesis]** Zhejiang University; 2015.
16. Clinical Trials website: **Immediate VS Delayed Cord Clamping on Newborns**. *https://clinicaltrialsgov/ct2/show/NCT01029496 last accessed August 30, 2018.*
17. Li J, Yu B, Wang W, Luo D, Dai Q-l, Gan X-q: **Does intact umbilical cord milking increase infection rates in preterm infants with premature prolonged rupture of membranes?** *The Journal of Maternal-Fetal & Neonatal Medicine* 2018:1-7.
18. Chinese Clinical Trial Registry website: **Delayed cord clamping prevents respiratory distress of infants delivered by selective cesarean section, a randomized controlled trial. http://www.chictr.org.cn/showprojen.aspx?proj=30199. Accessed February 20, 2019**.
19. Shi W, Peng J, Chen N: **Effect of delayed cord clamping on outcome of delivery**. *Henan Journal of Preventive Medicine* 2017, **28**:85-87.
20. Clinical Trials website: **The Study on Umbilical Cord Milking to Prevent and Decrease the Severity of Anemia in Preterms**. *https://clinicaltrialsgov/ct2/show/NCT03023917 last accessed August 31, 2018.*
21. Australian New Zealand Clinical Trials Registry: **Delayed fetal cord clamping in preterm labour, Risk and benefits. https://www.anzctr.org.au/Trial/Registration/TrialReview.aspx?id=374731. Accessed February 21, 2019.**
22. ClinicalTrials.gov registry: **Impact of Umbilical Cord Milking in Preterm Neonates With Placental Insufficiency**. *https://clinicaltrialsgov/ct2/show/NCT03731611 Accessed February 21, 2019.*
23. ClinicalTrials.gov registry: **Effect of Delayed Cord Clamping in Preterm Neonates With Placental Insufficiency, available at https://clinicaltrials.gov/ct2/show/NCT03731546. Last accessed Feb 21, 2019.**
24. Nelle M, Fisher S, Conze S, Beedgen B, Linderkamp O: **Effects of late cord clamping on circulation in prematures (VLBWI)**. *Pediatric Research* 1998, **44**(3):454.

For peer review only

iCOMP

25. Rabe H, Wacker A, Hülskamp G, Hörnig-Franz I, Schulze-Everding A, Harms E, Cirkel U, Louwen F, Witteler R, Schneider HP: **A randomised controlled trial of delayed cord clamping in very low birth weight preterm infants**. *Eur J Pediatr* 2000, **159**(10):775-777.
26. ClinicalTrials.gov registry: **Umbilical Cord Milking in Neonates Who Are Depressed at Birth (MIDAB). Available at https://clinicaltrials.gov/ct2/show/NCT03657394. Last accessed February 21, 2019.**
27. Clinical Trials Registry India website: **Comparison of advantages and disadvantages of early and delayed umbilical cord clamping in low birth weight newborns. http://ctri.nic.in/Clinicaltrials/pdf_generate.php?trialid=16653&EncHid=&modid=&compid=%27,%2716653det%27 last accessed February 21, 2019.**
28. Clinical Trials Registry India website: **The effects of umbilical cord squeezing in newborn babies requiring some form of intervention to help and support breathing at birth. http://www.ctri.nic.in/Clinicaltrials/pdf_generate.php?trialid=18641&EncHid=&modid=&compid=%27,%2718641det%27, last accessed February 21, 2019.**
29. Chopra A, Thakur A, Garg P, Kler N, Gujral K: **Early versus delayed cord clamping in small for gestational age infants and iron stores at 3 months of age-a randomized controlled trial**. *BMC pediatrics* 2018, **18**(1):234.
30. Das B, Sundaram V, Tarnow-Mordi W, Ghadge A, Dhaliwal LK, Kumar P: **Placental transfusion in preterm neonates of 30–33 weeks' gestation: a randomized controlled trial**. *Journal of Perinatology* 2018, **38**:496–504.
31. Datta V, Kumar A, Yadav R: **A randomized controlled trial to evaluate the role of brief delay in cord clamping in preterm neonates (34-36 weeks) on short-term neurobehavioural outcome**. *Journal of Tropical Pediatrics* 2017, **63**(6):418–424.
32. Dhaliwal LK, Anjumunnisa S, Kumar P, Saha PK, Venkataseshan S: **Effects of placental transfusion in preterm birth**. *J Matern Fetal Neonatal Med* 2014, **27**(S1):369-370.
33. Dipak NK, Nanavat RN, Kabra NK, Srinivasan A, Ananthan A: **Effect of Delayed Cord Clamping on Hematocrit, and Thermal and Hemodynamic Stability in Preterm Neonates: A Randomized Controlled Trial.** *Indian Pediatr* 2017, **54**(2):112-115.
34. Clinical Trials Registry India website: **Effect of umbilical cord milking of late preterm and term infants on maternal and neonatal outcomes in a tertiary care hospital in South India: A randomized control trial. http://www.ctri.nic.in/Clinicaltrials/pmaindet2.php?trialid=19631 last accessed February 21, 2019**.
35. Clinical Trials Registry India website: **Early versus Delayed Cord clamping in IUGR Preterms a Randomised controlled study. http://ctri.nic.in/Clinicaltrials/showallp.php?mid1=25064&EncHid=&userName=015204 last accessed February 21, 2019.**
36. Kumar B, Upadhyay A, Gothwal S, Jaiswal V, Joshi P, Dubey K: **Umbilical Cord Milking and Hematological Parameters in Moderate to Late Preterm Neonates: A Randomized Controlled Trial**. *Indian Pediatr* 2015, **52**(9):753-757.
37. Clinical Trials Registry India website: **Umbilical cord milking in preterm neonates requiring resuscitation: A randomized controlled trial** *http://wwwctrinicin/Clinicaltrials/pmaindet2php?trialid=10609 last accessed August 31, 2018.*
38. Rana A, Agarwal K, Ramji S, Gandhi G, Sahu L: **Safety of delayed umbilical cord clamping in preterm neonates of less than 34 weeks of gestation: a randomized controlled trial**. *Obstetrics & Gynecology Science* 2018, **61**(6):655-661.
39. Ranjit T, Nesargi S, Rao PNS, Sahoo JP, Ashok C, Chandrakala BS, Bhat S: **Effect of Early versus Delayed Cord Clamping on Hematological Status of Preterm Infants at 6 wk of Age**. *The Indian Journal of Pediatrics* 2015, **82**(1):29-34.

For peer review only - http://bmjopen.bmj.com/site/about/guidelines.xhtml

40. Clinical Trials Registry India website: **Comparison of Umbilical cord milking with Delayed cord clamping in late preterm and term neonates: Randomized control trial. www.ctri.nic.in/Clinicaltrials/pmaindet2.php?trialid=14204. Accessed August 31, 2018**.

41. CLinical Trials Registry India website: **Effect of cord milking on hemodynemic status and morbidities in neonates more than 35 weeks of gestation**. *http://wwwctrinicin/Clinicaltrials/pmaindet2php?trialid=3182 last accessed August 31, 2018*.

42. ClinicalTrials.gov registry: **Effect of Intact Umbilical Cord Milking on Neonatal and First Year Neurodevelopmental Outcomes in Very Preterm Infants. (Cord Milking)**. *https://clinicaltrialsgov/ct2/show/NCT03200301 last accessed February 22, 2019*.

43. Armanian AM, Ghasemi Tehrani H, Ansari M, Ghaemi S: **Is "Delayed Umbilical Cord Clamping" Beneficial for Premature Newborns?** *Int J Pediatr* 2017, **5**(5):4909-4918.

44. Kazemi MV, Akbarianrad Z, Zahedpasha Y, Mehraein R, Mojaveri MH: **Effects of Delayed Cord Clamping on Intraventricular Hemorrhage in Preterm Infants**. *Iran J Pediatr* 2017 **27**(5):e6570.

45. Iranian Registry of Clinical Trials website: **Investigation and comparison of neonatal complications of two methods of umbilical cord milking and early cord clamping in neonates**. *http://enirctir/trial/29424 last accessed February 22, 2019*.

46. Sekhavat L, Tabatabaii A: **Immediate and delayed cord clamping in infants born between 26 and 34 weeks**. *Journal of Maternal-Fetal and Neonatal Medicine* 2008, **21**((Suppl 1)):181–182.

47. Iranian Registry of Clinical Trials website: **The delayed umbilical cord clamping effects on early outcome in Preterm neonates**. *http://enirctir/trial/17924 last accessed February 22, 2019*.

48. ISRCTN registry website: **Clamping the umbilical cord in premature deliveries (CUPID)**. *http://wwwisrctncom/ISRCTN92719670 last accessed August 27, 2018*.

49. Kugelman A, Borenstein-Levin L, Riskin A, Chistyakov I, Ohel G, Gonen R, Bader D: **Immediate versus delayed umbilical cord clamping in premature neonates born < 35 weeks: a prospective, randomized, controlled study**. *Am J Perinatol* 2007, **24**(5):307-315.

50. Hosono S, Mugishima H, Fujita H, Hosono A, Minato M, Okada T, Takahashi S, Harada K: **Umbilical cord milking reduces the need for red cell transfusions and improves neonatal adaptation in infants born at less than 29 weeks' gestation: a randomised controlled trial**. *Arch Dis Child Fetal Neonatal Ed* 2008, **93**(1):F14-19.

51. UMIN clinical trials registry: **A multicenter randomized control study of the effect of umbilical cord milking**. *https://uploaduminacjp/cgi-open-bin/ctr_e/ctr_viewcgi?recptno=R000001193 last accessed 31 August, 2018*.

52. Song SY, Kim Y, Kang BH, Yoo HJ, Lee M: **Safety of umbilical cord milking in very preterm neonates: a randomized controlled study**. *Obstet Gynecol Sci* 2017, **60**(6):527-534.

53. Zisovska E, Lazarevska L, Spasova L, Zivkovik J: **The effect of delayed cord clamping on the need for blood transfusion: P17**. *Transfusion Alternatives in Transfusion Medicine* 2008, **10**.

54. Clinical Trials website: **Effect of Timing of Umbilical Cord Clamping on Anaemia at 8 and 12 Months and Later Neurodevelopment**. *https://clinicaltrialsgov/ct2/show/NCT02222805 last accessed August 27, 2018*.

55. ISRCTN registry website: **Effect of timing of cord clamping in newborns during first 10 minutes of birth**. *http://wwwisrctncom/ISRCTN10944304 last accessed August 27, 2018*.

For peer review only

For peer review only - http://bmjopen.bmj.com/site/about/guidelines.xhtml

56. ClinicalTrials.gov registry: **Aeration, Breathing, Clamping Study 3 (ABC3)**. https://clinicaltrialsgov/ct2/show/NCT03808051 *last accessed February 22, 2019*.

57. Ultee CA, van der Deure J, Swart J, Lasham C, van Baar AL: **Delayed cord clamping in preterm infants delivered at 34 36 weeks' gestation: a randomised controlled trial**. *Arch Dis Child Fetal Neonatal Ed* 2008, **93**(1):F20-23.

58. Malik AU, Shahnawaz K, Riaz A: **Comparison between the Efficacy of Early and Delayed Umbilical Cord Clamping in Preterm Infants**. *Pak J Med Health Sci* 2013, **7**(4):992-995.

59. ClinicalTrials.gov registry: **Deferred Cord Clamping Compared to Umbilical Cord Milking in Preterm Infants**. https://clinicaltrialsgov/ct2/show/NCT02996799 *last accessed February 22, 2019*.

60. ClinicalTrials.gov registry: **The Hematologic Impact of Umbilical Cord Milking Versus Deferred Cord Clamping in Premature Neonates**. https://clinicaltrialsgov/ct2/show/NCT03147846 *last accessed February 22, 2019*.

61. Hofmeyr GJ, Bolton KD, Bowen DC, Govan JJ: **Periventricular/intraventricular haemorrhage and umbilical cord clamping. Findings and hypothesis.** *S Afr Med J* 1988, **73**(2):104-106.

62. Hofmeyr GJ, Gobetz L, Bex PJM, Van Der Griendt M, Nikodem C, Skapinker R, Delahunt T: **Periventricular/intraventricular hemorrhage following early and delayed umbilical cord clamping. A randomized controlled trial.** . *Online J Curr Clin Trials* 1993(110).

63. Tiemersma S, Heistein J, Ruijne R, Lopez G, van Lobenstein J, van Rheenen P: **Delayed cord clamping in South African neonates with expected low birthweight: a randomised controlled trial**. *Tropical Medicine & International Health* 2015, **20**(2):177-183.

64. ISRCTN registry website: **DElayed COrd CLAmping versus early cord clamping in preterm infants born between 24 and 34 weeks**. http://wwwisrctncom/ISRCTN66018314 *last accessed August 27, 2018*.

65. Clinical Trials website: **Umbilical Cord Milking vs Delayed Cord Clamping in Preterm Infants Born by Cesarean Section**. https://clinicaltrialsgov/ct2/show/NCT02187510 *last accessed August 30, 2018*.

66. Lago Leal V, Pamplona Bueno L, Cabanillas Vilaplana L, Nicolas Montero E, Martin Blanco M, Fernandez Romero C, El Bakkali S, Pradillo Aramendi T, Sobrino Lorenzano L, Castellano Esparza P *et al*: **Effect of Milking Maneuver in Preterm Infants: A Randomized Controlled Trial**. *Fetal diagnosis and therapy* 2019, **45**(1):57-61.

67. Clinical Trials website: **Timing of Umbilical Cord Occlusion in Premature Babies(<33 w). Delayed vs Early. (CODE-P)**. https://clinicaltrialsgov/ct2/show/NCT02187874 *last accessed August 27, 2018*.

68. Baenziger O, Stolkin F, Keel M, von Siebenthal K, Fauchere JC, Das Kundu S, Dietz V, Bucher HU, Wolf M: **The influence of the timing of cord clamping on postnatal cerebral oxygenation in preterm neonates: a randomized, controlled trial**. *Pediatrics* 2007, **119**(3):455-459.

69. ISRCTN registry website: **Can squeezing blood from the umbilical cord into the baby reduce death and illness in premature babies? http://www.isrctn.com/ISRCTN17228884 last accessed February 26, 2019**.

70. Thai Clinical Trials Registry website: **Effect of delayed cord clamping versus cord milking in infants born at < 34 weeks gestation: a randomized controlled trial**. http://wwwclinicaltrialsinth/indexphp?tp=regtrials&menu=trialsearch&smenu=fulltext&task=search&task2=view1&id=1277 *last accessed August 31, 2018*.

For peer review only - http://bmjopen.bmj.com/site/about/guidelines.xhtml

iCOMP

71. Thai Clinical Trials Registry website: **Delayed cord clamping reduced anemic outcome in preterm neonate, http://www.clinicaltrials.in.th/index.php?tp=regtrials&menu=trialsearch&smenu=fulltext&task=search&task2=view1&id=3833 last accessed Feb 25, 2019.**

72. Mungkornkaew S, Siwadune T: **The Difference of Hematocrit in Term and Preterm Vaginal Births in Different Timing of Delayed Cord Clamping**. *Thai Journal of Obstetrics and Gynaecology* 2015:223-230.

73. Thai Clinical Trials Registry website: **Effects of delayed versus early cord clamping in late preterm infants: a randomized controlled trial**. *http://wwwclinicaltrialsinth/indexphp?tp=regtrials&menu=trialsearch&smenu=fulltext&task=search&task2=view1&id=1279 last accessed August 29, 2018*.

74. Thai Clinical Trials Registry website: **A Randomized Controlled Trial of Immediate versus Delayed Umbilical Cord Clamping in Preterm Infants of Multiple Births**. *http://wwwclinicaltrialsinth/indexphp?tp=regtrials&menu=trialsearch&smenu=fulltext&task=search&task2=view1&id=2189 last accessed August 29, 2018*.

75. Salae R, Tanprasertkul C, Somprasit C, Bhamarapravatana K, Suwannarurk K: **Efficacy of delayed versus immediate cord clamping in late preterm newborns following normal labor: a randomized control trial**. *J Med Assoc Thai* 2016, **99**(4):S159-S165.

76. Thai Clinical Trials Registry website: **The Effect of One-Time Umbilical Cord Milking and Early Cord Clamping in Preterm Infants: A Randomized Controlled Trial (One-Time Umbilical Cord Milking)**. *http://clinicaltrialsinth/indexphp?tp=regtrials&menu=trialsearch&smenu=fulltext&task=search&task2=view1&id=2347 last accessed August 31, 2018*.

77. Alan S, Arsan S, Okulu E, Akin IM, Kilic A, Taskin S, Cetinkaya E, Erdeve O, Atasay B: **Effects of Umbilical Cord Milking on the Need for Packed Red Blood Cell Transfusions and Early Neonatal Hemodynamic Adaptation in Preterm Infants Born ≤1500 g: A Prospective, Randomized, Controlled Trial**. *Journal of Pediatric Hematology/Oncology* 2014, **36**(8):e493-e498.

78. Gokmen Z, Ozkiraz S, Tarcan A, Kozanoglu I, Ozcimen Emel E, Ozbek N: **Effects of delayed umbilical cord clamping on peripheral blood hematopoietic stem cells in premature neonates**. *Journal of Perinatal Medicine* 2011, **39**:323–329.

79. Kilicdag H, Gulcan H, Hanta D, Torer B, Gokmen Z, Ozdemir SI, Antmen BA: **Is umbilical cord milking always an advantage?** *The Journal of Maternal-Fetal & Neonatal Medicine* 2016, **29**(4):615-618.

80. Silahli M, Duman E, Gokmen Z, Toprak E, Gokdemir M, Ecevit A: **The relationship between placental transfusion, and thymic size and neonatal morbidities in premature infants - A Randomized Control Trial**. *JPMA The Journal of the Pakistan Medical Association* 2018, **68**(11):1560-1565.

81. Aladangady N, McHugh S, Aitchison TC, Wardrop CA, Holland BM: **Infants' blood volume in a controlled trial of placental transfusion at preterm delivery**. *Pediatrics* 2006, **117**(1):93-98.

82. Duley L, Dorling J, Pushpa-Rajah A, Oddie SJ, Yoxall CW, Schoonakker B, Bradshaw L, Mitchell EJ, Fawke JA: **Randomised trial of cord clamping and initial stabilisation at very preterm birth**. *Archives of Disease in Childhood - Fetal and Neonatal Edition* 2018, **103**(1):F6-F14.

83. Kinmond S, Aitchison TC, Holland BM, Jones JG, Turner TL, Wardrop CA: **Umbilical cord clamping and preterm infants: a randomised trial**. *BMJ (Clinical research ed)* 1993, **306**(6871):172-175.

84. Medina IMF: **Late clamping of the umbilical cord in premature neonates: The real haemodynamic benefits**. *Enfermeria Clinica* 2014, **24**(5):305-308.

For peer review only

For peer review only - http://bmjopen.bmj.com/site/about/guidelines.xhtml

85. Rabe H, Jewison A, Alvarez RF, Crook D, Stilton D, Bradley R, Holden D: **Milking compared with delayed cord clamping to increase placental transfusion in preterm neonates: a randomized controlled trial**. *Obstetrics & Gynecology* 2011, **117**(2):205-211.
86. Backes CH, Huang H, Iams JD, Bauer JA, Giannone PJ: **Timing of umbilical cord clamping among infants born at 22 through 27 weeks' gestation**. *J Perinatol* 2016, **36**(1):35-40.
87. Clinical Trials website: **Effects of Delayed Cord Clamp and/or Indomethacin on Preterm Infant Brain Injury**. *https://clinicaltrialsgov/ct2/show/NCT02221219 last accessed August 27, 2018*.
88. ClinicalTrials.gov registry: **Relationship Between Delayed Cord Clamping at Birth and Neonatal Bilirubin Levels in Parturients With a Prior Child Requiring Therapy for Neonatal Jaundice, https://clinicaltrials.gov/ct2/show/NCT03741803 last accessed February 25th, 2019**.
89. Clinical Trials website: **Milking the Umbilical Cord Versus Immediate Clamping in Pre-term Infants < 33 Weeks**. *https://clinicaltrialsgov/ct2/show/NCT01819532 last accessed August 30, 2018*.
90. March MI, Hacker MR, Parson AW, Modest AM, de Veciana M: **The effects of umbilical cord milking in extremely preterm infants: A randomized controlled trial**. *J Perinatol* 2013, **33**(10):763-767.
91. ClinicalTrials.gov registry: **Delayed Umbilical Cord Clamping Versus Cord Milking in Preterm Neonate, https://clinicaltrials.gov/ct2/show/NCT01393834 last accessed 25th February, 2019.**
92. Clinical Trials website: **A Randomized Controlled Trial of Immediate Versus Delayed Cord Clamping in the Preterm Neonate**. *https://clinicaltrialsgov/ct2/show/NCT00579839 last accessed August 27, 2018*.
93. Ibrahim HM, Krouskop RW, Lewis DF, Dhanireddy R: **Placental transfusion: umbilical cord clamping and preterm infants**. *Journal of Perinatology* 2000, **20**(6):351.
94. Josephsen J, Vlastos E, Potter S, Al-Hosni M: **Milking the umbilical cord in extreme preterm infants**. *Am J Obstet Gynecol* 2014, **10**(1 Suppl):S403-404.
95. Katheria AC, Leone TA, Woelkers D, Garey DM, Rich W, Finer NN: **The effects of umbilical cord milking on hemodynamics and neonatal outcomes in premature neonates**. *J Pediatrics* 2014, **164**(5):1045-1050.
96. Katheria AC, Truong G, Cousins L, Oshiro B, Finer NN: **Umbilical cord milking versus delayed cord clamping in preterm infants**. *Pediatrics* 2015, **136**(1):61-69.
97. Katheria A, Poeltler D, Durham J, Steen J, Rich W, Arnell K, Maldonado M, Cousins L, Finer N: **Neonatal Resuscitation with an Intact Cord: A Randomized Clinical Trial**. *Journal of Pediatrics* 2016, **178**:75-80.e73.
98. ClinicalTrials.gov registry: **Premature Infants Receiving Milking or Delayed Cord Clamping: PREMOD2. Available at https://clinicaltrials.gov/ct2/show/NCT03019367. Accessed August 23, 2019.**
99. ClinicalTrials.gov registry: **Umbilical Cord Milking in Non-Vigorous Infants (MINVI). Available at https://clinicaltrials.gov/ct2/show/NCT03631940. Accessed August 23, 2019.**
100. Clinical Trials website: **VentFirst: A Multicenter RCT of Assisted Ventilation During Delayed Cord Clamping for Extremely Preterm Infants**. *https://clinicaltrialsgov/ct2/show/NCT02742454 last accessed August 27, 2018*.
101. Krueger MS, Eyal FG, Peevy KJ, Hamm CR, Whitehurst RM, Lewis DF: **Delayed cord clamping with and without cord stripping: A prospective randomized trial of preterm neonates**. *Am J Obstet Gynecol* 2015, **212**(3):394.e391-395.

For peer review only - http://bmjopen.bmj.com/site/about/guidelines.xhtml

iCOMP

102. Clinical Trials website: **Optimal Timing of Cord Clamping in Preterm Pregnancy Following Vaginal or Cesarean Delivery (CordClamp)**. *https://clinicaltrialsgov/ct2/show/NCT01766908 last accessed August 27, 2018*.
103. Mercer JS, McGrath MM, Hensman A, Silver H, Oh W: **Immediate and delayed cord clamping in infants born between 24 and 32 weeks: a pilot randomized controlled trial**. *Journal of perinatology* 2003, **23**(6):466.
104. Mercer JS, Vohr BR, McGrath MM, Padbury JF, Wallach M, Oh W: **Delayed cord clamping in very preterm infants reduces the incidence of intraventricular hemorrhage and late-onset sepsis: a randomized, controlled trial**. *Pediatrics* 2006, **117**(4):1235-1242.
105. Mercer JS, Erickson-Owens DA, Vohr BR, Tucker RJ, Parker AB, Oh W, Padbury JF: **Effects of placental transfusion on neonatal and 18 month outcomes in preterm infants: a randomized controlled trial**. *J Pediatrics* 2016, **168**:50-55.
106. Oh W, Fanaroff AA, Carlo WA, Donovan EF, McDonald SA, Poole WK, Eunice Kennedy Shriver National Institute of Child Health and Human Development Neonatal Research Network: **Effects of delayed cord clamping in very-low-birth-weight infants**. *J Perinatol* 2011, **31**:S68-S71.
107. Clinical Trials website: **Delayed Cord Clamping in Preterm Neonates (DCC)**. *https://clinicaltrialsgov/ct2/show/NCT02478684 last accessed August 29, 2018*.
108. Clinical Trials website: **Delayed Clamping and Milking the Umbilical Cord in Preterm Infants**. *https://clinicaltrialsgov/ct2/show/NCT02092103 last accessed August 31, 2018*.
109. Strauss RG, Mock DM, Johnson KJ, Cress GA, Burmeister LF, Zimmerman MB, Bell EF, Rijhsinghani A: **A randomized clinical trial comparing immediate versus delayed clamping of the umbilical cord in preterm infants: short-term clinical and laboratory endpoints**. *Transfusion* 2008, **48**(4):658-665.
110. ClinicalTrials.gov registry: **Delayed Cord Clamping in Very Low Birth Weight Infants (DCC). Available at https://clinicaltrials.gov/ct2/show/NCT02337088. Accessed August 23, 2019.**
111. Pongmee P, Nuntnarumit P: **Effects of umbilical cord milking on initial hematocrit and the need for blood transfusion in preterm infants. In: Pediatric Academic Societies Annual Meeting; 2010 May 1-4; Vancouver, Canada. 2010**.

For peer review only

For peer review only - http://bmjopen.bmj.com/site/about/guidelines.xhtml

Supplementary File 2: PRISMA-P checklist

**Section and topic**	**Item no.**	**Checklist item**	**Page number**
Title	1a	Identify the report as a protocol of a systematic review	p.1
	1b	If protocol is for an update of a previous review, identify as such	Not applicable
Registration	2	If registered, provide the name of the registry and registration number.	p.2
Authors	3a	Provide name, institutional affiliation, email address of all protocol authors, provide physical mailing address of corresponding author.	p.1
	3b	Describe contributions of protocol authors and identity the guarantor of the review.	p.16
Amendments	4	If the protocol represents an amendment of a previously completed or published protocol, identify as such and list changes; otherwise, state plan for documenting important protocol amendments.	p.11, p.12
Support	5a	Indicate sources of financial or other support for the review.	p.16
	5b	Provide name for review funder and/or sponsor.	p.16
	5c	Describe roles of funder(s), sponsor(s), and/or institution(s), if any, in developing the protocol.	p.16
Rationale	6	Describe the rational for the review in the context of what is already known.	p.4-5
Objectives	7	Provide an explicit statement of the question(s) the review will address with reference to participants, interventions, comparators and outcomes (PICO)	p.6
Eligibility criteria	8	Specify the study characteristics (such as PICO, study design, setting time frame) and report characteristics (such as years considered, language, publication status) to be used as criteria for eligibility for the review.	p.6-8
Information sources	9	Describe all intended information sources (such as electronic databases, contact with study authors, trial registers or other grey literature sources) with planned dates of coverage.	p.8-9
Search strategy	10	Present draft of search strategy to be used for at least one electronic database, including planned limits, such that it could be repeated.	Supplementary File 3

For peer review only

Study records	11a	Describe the mechanism(s) that will be used to manage records and data throughout the review.	p.8 ff.
	11b	State the process that will be used for selecting studies (such as two independent reviewers) through each phase of the review (that is, screening, eligibility and inclusion in meta-analysis).	p.8
	11c	Describe planned method of extracting data from reports (such as piloting forms, done independently, in duplicate), any processes for obtaining and confirming data from investigators.	p.8 ff.
Data items	12	List and define all variables for which data will be sought (such as PICO items, funding sources), and pre-planned data assumptions and simplifications.	p.9 ff.
Outcomes and prioritization	13	List and define all outcomes for which data will be sought, including prioritization of main and additional outcomes, with rationale.	p.9 ff.
Risk of bias in individual studies	14	Describe anticipated methods for assessing risk of bias of individual studies, including whether this will be done at the outcome or study level, or both; state how this information will be used in data synthesis.	p.9
Data synthesis	15a	Describe criteria under which study data will be quantitatively synthesised.	p.12, p.13
	15b	If data are appropriate for quantitative synthesis, describe planned summary measures, methods of handling data and methods of combining data from studies, including any planned exploration of consistency (such as I^2, Kendall's τ).	p.12, p.13
	15c	Describe any proposed additional analyses (such as sensitivity or subgroup analyses, meta-regression).	p.14
	15d	If quantitative synthesis is not appropriate, describe the type of summary planned.	n.a.
Meta-bias(es)	16	Specify any planned assessment of meta-bias(es) (such as publication bias across studies, selective reporting within studies).	p.14
Confidence in cumulative evidence	17	Describe how the strength of the body of evidence will be assessed (such as GRADE).	p.9

For peer review only

iCOMP search strategy

1. We used search results from a prospective meta-analysis we had previously planned to conduct on cord clamping in preterm infants. Regular searches were conducted from 2010 to 2017.
2. We used search results up to November 2018 from a recently updated Cochrane review on cord clamping in preterm infants, on which some of us are authors (Rabe H, Gyte GML, Díaz-Rossello JL, Duley L. Effect of timing of umbilical cord clamping and other strategies to influence placental transfusion at preterm birth on maternal and infant outcomes. Cochrane Database of Systematic Reviews 2019, Issue 9. Art. No.: CD003248. DOI: 10.1002/14651858.CD003248.pub4.)
3. We conducted new independent searches for the period from November 2018 onwards.

Details of all searches are elucidated below.

1. Search methods – for previously planned PMA (up to September 2017)

We regularly searched the World Health Organisation (WHO) International Clinical Trials Registry Platform (ICTRP) from the period January 2010 to September 2017. In total, fourteen searches were conducted over this period, using a combination of the search terms shown below.

1.1 ICTRP

1. placental transfusion
2. cord clamp*
3. umbilical cord clamp*
4. cord milking
5. milking
6. umbilical cord
7. preterm
8. pre-term
9. prematur*

2. Search methods - Cochrane review update (up to November 2018)

The following sources were searched: Cochrane Pregnancy and Childbirth's Trials Register, ClinicalTrials.gov and WHO ICTRP. Further details of each are provided below.

2.1 Pregnancy and Childbirth's Trials Register

- Searched 8 November 2018 by the Cochrane Pregnancy and Childbirth Information Specialist.
- For detailed information about the registry and search strategies, please go to https://pregnancy.cochrane.org/pregnancy-and-childbirth-groups-trials-register.

2.2 ICTRP

- Searched 8 November 2018

cord AND clamp
cord and clamping
cord AND milking
cord AND stripping

2.3 ClinicalTrials.gov

- Searched 8 November 2018

For peer review only

Advanced search

Interventional studies | cord clamping
Interventional studies | cord milking
Interventional studies | cord stripping

3. Search methods (from November 2018)

We searched Ovid MEDLINE, Embase, EBM Reviews - Cochrane Central Register of Controlled Trials, WHO ICTRP and ClinicalTrials.gov using the search strings below.

3.1 Ovid MEDLINE(R)

1. umbilical-cord.mp. or exp umbilical cord/
2. (Clamp$ OR Milk$).af.
3. (Placenta$ adj2 transfus$).af
4. 2 or 3
5. exp Infant, Premature/ or preterm*.mp.
6. prematur*.mp.
7. exp Infant, Low Birth Weight/ or exp Infant, Very Low Birth Weight
8. exp Infant, Extremely Low Birth Weight
9. 5 or 6 or 7 or 8
10. 1 and 4 and 9
11. limit 10 to (humans and clinical trial, all)
12. limit 11 to ed=20181001-20190213

3.2 Embase

1. umbilical-cord.mp. or exp umbilical cord/
2. (Clamp$ OR Milk$).af.
3. (Placenta$ adj2 transfus$).af
4. 2 or 3
5. exp Infant, Premature/ or preterm*.mp.
6. prematur*.mp.
7. exp Infant, Low Birth Weight/ or exp Infant, Very Low Birth Weight
8. exp Infant, Extremely Low Birth Weight
9. 5 or 6 or 7 or 8
10. 1 and 4 and 9
11. limit 10 to (human and randomized controlled trial)
12. limit 11 to yr="2018 -Current"

3.3 EBM Reviews - Cochrane Central Register of Controlled Trials

1. umbilical-cord.mp. or exp umbilical cord/
2. (Clamp$ OR Milk$).af.
3. (Placenta$ adj2 transfus$).af
4. 2 or 3
5. exp Infant, Premature/ or preterm*.mp.
6. prematur*.mp.
7. exp Infant, Low Birth Weight/ or exp Infant, Very Low Birth Weight
8. exp Infant, Extremely Low Birth Weight
9. 5 or 6 or 7 or 8
10. 1 and 4 and 9
11. limit 10 to yr="2018 -Current"

For peer review only

3.4 WHO ICTRP

Search string
Basic search
1. placental transfusion<br>(limit date of registration from 1/11/2018 onwards)
2. cord clamp<br>(limit date of registration from 1/11/2018 onwards)
3. cord clamping<br>(limit date of registration from 1/11/2018 onwards)
4. milking<br>(limit date of registration from 1/11/2018 onwards)
Advanced search
5. Title: umbilical cord<br>Condition: preterm OR premature<br>Recruitment Status: All<br>(limit date of registration from 1/11/2018 onwards)
6. Condition: preterm OR premature<br>Intervention: "umbilical cord"<br>Recruitment Status: All<br>(limit date of registration from 1/11/2018 onwards)

3.5 Clinicaltrials.gov

Search string
Basic search
1. Other terms: "placental transfusion"<br>First posted from 11/01/2018 to 02/13/2019 (MM/DD/YYYY)
2. Other terms: "cord clamp"<br>First posted from 11/01/2018 to 02/13/2019 (MM/DD/YYYY)
3. Other terms: "cord clamping"<br>First posted from 11/01/2018 to 02/13/2019 (MM/DD/YYYY)
4. Other terms: milking<br>First posted from 11/01/2018 to 02/13/2019 (MM/DD/YYYY)
5. Condition or disease: Preterm Birth<br>Other terms: "umbilical cord"<br>First posted from 11/01/2018 to 02/13/2019 (MM/DD/YYYY)

For peer review only

BMJ Open

Systematic review and network meta-analysis with individual participant data on Cord Management at Preterm Birth (iCOMP): study protocol

Journal:	*BMJ Open*
Manuscript ID	bmjopen-2019-034595.R1
Article Type:	Protocol
Date Submitted by the Author:	22-Jan-2020
Complete List of Authors:	Seidler, Anna Lene; The University of Sydney, NHMRC Clinical Trials Centre<br>Duley, Lelia; University of Nottingham, Nottingham Clinical Trials Unit<br>Katheria, Anup; Sharp Healthcare<br>De Paco Matallana, Catalina; Clinic University Hospital Virgen de la Arrixaca<br>Dempsey, E; Cork University Maternity Hospital<br>Rabe, Heike; Brighton and Sussex University Hospitals, Academic Department of Paediatrics<br>Kattwinkel, John; University of Virginia<br>Mercer, Judith; University of Rhode Island, College of Nursing<br>Josephsen, Justin; St Louis University School of Medicine<br>Fairchild, Karen; University of Virginia<br>Andersson, Ola; Uppsala Universitet<br>Hosono, Shigeharu; Jichi Ika University Saitama Medical Center<br>Sundaram, Venkataseshan; Postgraduate Institute of Medical Education and Research, Newborn unit, Dept of Pediatrics<br>Datta, Vikram; Lady Hardinge Medical College<br>El-Naggar, Walid; Dalhousie University, Department of Pediatrics<br>Tarnow-Mordi, William; The University of Sydney, NHMRC Clinical Trials Centre<br>Debray, Thomas; University Medical Center Utrecht, Julius Center for Health Sciences and Primary Care<br>Hooper, Stuart B.; Monash University, The Ritchie Centre, Obstetrics & Gynaecology<br>Kluckow, Martin; University of Sydney,<br>Polglase, Graeme; Monash University, The Ritchie Centre, Obstetrics & Gynaecology<br>Davis, Peter; The Royal Women's Hospital, Newborn Research<br>Montgomery, Alan; University of Nottingham, Nottingham Clinical Trials Unit<br>Hunter, Kylie; NHMRC Clinical Trials Centre, University of Sydney<br>Barba, Angie; The University of Sydney, NHMRC Clinical Trials Centre<br>Simes, John; University of Sydney, NHMRC Clinical Trials Centre<br>Askie, Lisa; University of Sydney, NHRMC Clinical Trials Centre
**Primary Subject Heading**:	Paediatrics

Secondary Subject Heading:	Obstetrics and gynaecology, Evidence based practice, Research methods
Keywords:	preterm birth, umbilical cord clamping, placental transfusion, umbilical cord milking, individual participant data meta-analysis, network meta-analysis

SCHOLARONE™ Manuscripts

I, the Submitting Author has the right to grant and does grant on behalf of all authors of the Work (as defined in the below author licence), an exclusive licence and/or a non-exclusive licence for contributions from authors who are: i) UK Crown employees; ii) where BMJ has agreed a CC-BY licence shall apply, and/or iii) in accordance with the terms applicable for US Federal Government officers or employees acting as part of their official duties; on a worldwide, perpetual, irrevocable, royalty-free basis to BMJ Publishing Group Ltd ("BMJ") its licensees and where the relevant Journal is co-owned by BMJ to the co-owners of the Journal, to publish the Work in this journal and any other BMJ products and to exploit all rights, as set out in our licence.

The Submitting Author accepts and understands that any supply made under these terms is made by BMJ to the Submitting Author unless you are acting as an employee on behalf of your employer or a postgraduate student of an affiliated institution which is paying any applicable article publishing charge ("APC") for Open Access articles. Where the Submitting Author wishes to make the Work available on an Open Access basis (and intends to pay the relevant APC), the terms of reuse of such Open Access shall be governed by a Creative Commons licence – details of these licences and which Creative Commons licence will apply to this Work are set out in our licence referred to above.

Other than as permitted in any relevant BMJ Author's Self Archiving Policies, I confirm this Work has not been accepted for publication elsewhere, is not being considered for publication elsewhere and does not duplicate material already published. I confirm all authors consent to publication of this Work and authorise the granting of this licence.

For peer review only

**Title**	**Systematic review and network meta-analysis with individual participant data on Cord Management at Preterm Birth (iCOMP): study protocol**
**Author names**	Anna Lene Seidler[1], Lelia Duley[2], Anup Katheria[3], Catalina De Paco Matallana[4], Eugene Dempsey[5], Heike Rabe[6], John Kattwinkel[7], Judith Mercer[8], Justin Josephsen[9], Karen Fairchild[7], Ola Andersson[10], Shigeharu Hosono[11], Venkataseshan Sundaram[12], Vikram Datta[13], Walid El-Naggar[14], William Tarnow-Mordi[1], Thomas P.A. Debray[15], Stuart Hooper[16], Martin Kluckow[17], Graeme Polglase[16], Peter Davis[18], Alan Montgomery[2], Kylie E Hunter[1], Angie Barba[1], John Simes[1] & Lisa Askie[1] on behalf of the iCOMP collaboration
**Author affiliations**	[1] National Health and Medical Research Council Clinical Trials Centre, University of Sydney, Sydney, Australia<br>[2] Nottingham Clinical Trials Unit, University of Nottingham, Nottingham, UK<br>[3] Sharp Healthcare, San Diego, California, USA<br>[4] Clinic University Hospital Virgen de la Arrixaca, Murcia, Spain<br>[5] Cork University Maternity Hospital, Cork, Ireland<br>[6] Brighton and Sussex Medical School, University of Sussex, Brighton, UK<br>[7] University of Virginia, Charlottesville, Virginia, USA<br>[8] The University of Rhode Island, Kingston, Rhode Island, USA<br>[9] St Louis University School of Medicine, St. Louis, Missouri, USA<br>[10] Uppsala University, Sweden<br>[11] Jichi Medical University Saitama Medical Center, Saitama, Japan<br>[12] Post Graduate Institute of Medical Education & Research, Chandigarh, India<br>[13] Lady Hardinge Medical College, New Delhi, India<br>[14] Department of Pediatrics, Dalhousie University, Halifax, Canada<br>[15] Julius Center for Health Sciences and Primary Care, University Medical Center Utrecht, Utrecht, the Netherlands<br>[16] The Ritchie Centre, Obstetrics & Gynaecology, Monash University, Melbourne, Australia<br>[17] Faculty of Medicine and Health, University of Sydney, Sydney, Australia<br>[18] The Royal Women's Hospital, Melbourne, Australia
**Corresponding author**	Correspondence to:<br>Anna Lene Seidler<br>NHMRC Clinical Trials Centre, University of Sydney<br>Locked Bag 77, Camperdown NSW 1450, Australia<br>lene.seidler@ctc.usyd.edu.au<br>+61 2 9562 5082
**Disclaimers**	None.
**Source(s) of support**	Developing the protocol and establishing the collaborative group was supported by the UK National Institute of Health Research (grant RPPG060910107). Funding to undertake data collection and data analysis for the iCOMP Collaboration has been provided by the Australian National Health and Medical Research Council (grant APP1163585).
**Word count**	4,995
**Number of figures and tables**	Number of figures: 2<br>Number to tables: 1
**Conflict of interest declaration**	Lelia Duley, Anup Katheria, Catalina De Paco Matallana, Eugene Dempsey, Heike Rabe, John Kattwinkel, Judith Mercer, Justin Josephsen, Karen Fairchild, Ola Andersson, Shigeharu Hosono, Venkataseshan Sundaram, Vikram Datta, Walid El-Naggar, and William Tarnow-Mordi are Chief Investigators for eligible trials.

For peer review only - http://bmjopen.bmj.com/site/about/guidelines.xhtml

ABSTRACT

Introduction: Timing of cord clamping and other cord management strategies may improve outcomes at preterm birth. However, it is unclear whether benefits apply to all preterm subgroups. Previous and current trials compare various policies, including time- or physiology-based deferred cord clamping, and cord milking. Individual participant data (IPD) enables exploration of different strategies within subgroups. Network meta-analysis (NMA) enables comparison and ranking of all available interventions using a combination of direct and indirect comparisons.

Objectives: 1) To evaluate the effectiveness of cord management strategies for preterm infants on neonatal mortality and morbidity overall and for different participant characteristics using IPD meta-analysis; and 2) to evaluate and rank the effect of different cord management strategies for preterm births on mortality and other key outcomes using NMA.

Methods and analysis: Systematic searches of Medline, Embase, clinical trial registries, and other sources for all ongoing and completed randomised controlled trials comparing cord management strategies at preterm birth (before 37 weeks' gestation) have been completed up to 13 February 2019, but will be updated regularly to include additional trials. IPD will be sought for all trials; aggregate summary data will be included where IPD are unavailable. First, deferred clamping and cord milking will be compared with immediate clamping in pairwise IPD meta-analyses. The primary outcome will be death prior to hospital discharge. Effect differences will be explored for pre-specified participant subgroups. Second, all identified cord management strategies will be compared and ranked in an IPD NMA for the primary outcome and the key secondary outcomes. Treatment effect differences by participant characteristics will be identified. Inconsistency and heterogeneity will be explored.

Ethics and dissemination: Approved by University of Sydney Human Research Ethics Committee (2018/886). Results will be relevant to clinicians, guideline-developers and policy-makers, and will be disseminated via publications, presentations, and media releases.

Registration: Australian New Zealand Clinical Trials Registry: ACTRN12619001305112; PROSPERO: CRD42019136640

KEYWORDS

Preterm birth, umbilical cord clamping, umbilical cord milking, placental transfusion, individual participant data meta-analysis, network meta-analysis, prospective meta-analysis

For peer review only

For peer review only - http://bmjopen.bmj.com/site/about/guidelines.xhtml

STRENGTHS AND LIMITATIONS OF THIS STUDY

- This will be the most comprehensive review to date of interventions for umbilical cord management in preterm infants and the findings will be highly relevant to clinicians and guideline developers
- The use of individual participant data will allow assessment of the best treatment option for key subgroups of participants
- Network meta-analysis will enable the comparison and ranking of all available treatment options using direct and indirect evidence
- For some of the trials it will not be possible to obtain individual participant data, so published aggregate results will be used instead
- Risk of bias in the primary trials will be assessed using Cochrane criteria, and certainty of evidence for the meta-analyses will be appraised using the GRADE approach for the pairwise comparisons, and the CINeMA approach for the network meta-analysis

For peer review only

For peer review only - http://bmjopen.bmj.com/site/about/guidelines.xhtml

INTRODUCTION

Currently over 15 million babies are born preterm annually and this number is rising.(1-3) Of these, 1.1 million die, and the morbidity and healthcare costs amongst survivors and their families are high, with preterm survivors having an increased risk of cognitive, developmental and behavioural difficulties, and chronic ill health.(4-9) Hence, even modest improvements in outcomes of preterm birth would substantially benefit the children, their families, and also health services. In uncompromised babies, deferring cord clamping has been shown to be beneficial and is now used in routine practice.(10) However, it is unclear whether these benefits apply to preterm babies who usually receive immediate neonatal care, and whether any benefits outweigh potential harms. In addition, there are multiple competing cord management strategies, such as clamping the cord at different times or milking the cord, and considerations of the infant's respiratory status, and it is currently unknown which strategy yields the best balance of benefits and harms.

Current approaches to cord clamping

One potential mechanism of deferring umbilical cord clamping is a net transfer of blood from the placenta to the baby known as "placental transfusion". If the cord is not clamped at birth immediately, blood flow between the placenta and the baby may continue for up to five minutes in term infants.(11-13) For preterm births, blood flow may continue for longer,(14) since a greater proportion of feto-placental circulating blood volume is still in the placenta.(15) This has led to time-based approaches to deferring cord clamping that have been shown to increase peak haematocrit and reduce the need for blood transfusions.(16) Yet, recent findings suggest that placental transfusion does not always occur - blood flow may continue without any net transfer, and sometimes net transfer may be to the placenta.(17) Initial neonatal care and stabilisation traditionally takes place on a resuscitation platform at the side of the room or in an adjacent room. Deferred cord clamping is thus often associated with a delay in neonatal care and this has led to concerns including delayed resuscitation and hypothermia (18) particularly for very preterm infants and infants assessed as requiring resuscitation. An alternate emerging strategy is to provide immediate neonatal care with the cord intact beside the woman using a mobile resuscitation trolley or on the mother's leg. (19-24)

Another potential mechanism of deferred clamping is allowing time for the infant to establish spontaneous breathing whilst still placentally supported. Immediate cord clamping before the infant has established breathing may be harmful since it can lead to large fluctuations in blood pressure, a period of hypoxia, and restricted cardiac function.(25) Animal and pilot human studies suggest that breathing and lung aeration before cord clamping can improve cardiovascular stability and oxygenation, and reduce intraventricular haemorrhage and infant mortality.(26-29) They also suggest that initial respiratory support before clamping the cord can improve cerebral oxygenation and blood pressure, and reduce cerebrovascular impairment compared with immediate cord clamping.(30, 31) This evidence has led to the rise of "physiological cord clamping" which defers clamping until after the onset of breathing. Yet, onset of breathing is not always easy to determine without the assistance of video or extra equipment, whilst timing to cord clamping can be easily measured. In an earlier study,(32) time of onset of breathing in preterm infants receiving gentle stimulation was related to time after birth – within a minute over 90% of preterm infants had begun spontaneous breathing.

For peer review only - http://bmjopen.bmj.com/site/about/guidelines.xhtml

Cord milking or stripping (pinching the umbilical cord close to the mother and moving the fingers towards the infant) may be a way to increase preterm blood volume without deferring clamping.(33) Yet, a preterm lamb model demonstrated that during cord milking there was a transient increase to carotid blood flow and pressure.(34) A recent trial comparing deferred cord clamping with cord milking was stopped early in the subgroup of extremely preterm infants (23-27 weeks), as the incidence of severe intraventricular haemorrhage was higher in the cord milking group.(35) Hence, the effect of cord milking in different populations needs further elucidation.

Current guidelines for cord management at birth and previous reviews of aggregate data

Current uncertainties in optimal cord management strategies are reflected in varying guidelines. The World Health Organization (WHO) recommends late cord clamping (36) unless resuscitation is required, the National Institute for Health and Care Excellence (NICE) recommends waiting for 30 seconds to 3 minutes if mother and baby are stable, (37) and the International Liaison Committee on Resuscitation Council (ILCOR) recommends a delay in cord clamping of at least 1 minute. If the baby is assessed as requiring resuscitation (which is the case in many preterm infants), (38) WHO recommends immediate clamping, (39) NICE recommends considering cord milking before clamping, and ILCOR concludes that there is insufficient evidence to make any recommendations. (38)

A 2012 Cochrane review of timing of cord clamping for preterm births (40) included 15 trials, with 738 infants, of which one trial (with 40 infants) compared cord milking with immediate cord clamping.(41) There was heterogeneity in the timing of cord clamping and gestational age at recruitment, and data were insufficient for reliable conclusions about any of the primary outcomes of the review. A systematic review and meta-analysis published in 2018 (including 18 trials with 2834 infants) compared the effect of deferred (≥30 seconds) versus early (<30 seconds) clamping in preterm infants, and found a reduction in the primary outcome of hospital mortality by 32% (Risk Ratio = 0.68, 95% Confidence Interval = 0.52-0.90). (16) There was heterogeneity in the definition of 'early cord clamping' ranging from less than 5 to 25 seconds, and "late cord clamping", ranging from 30 to 180 seconds. Recruitment age varied from 22 weeks to 36 weeks gestational age. Most analyses of infant and maternal morbidity were substantially underpowered. (16) The review concluded that while there is high quality evidence that deferred cord clamping improves outcomes, individual participant data analyses are urgently needed to further understand the benefits and potential harms of different cord management strategies, and to understand whether differential treatment options are advantageous for key subgroups of infants. (16)

This ongoing uncertainty about the optimal cord management strategy, and differential cord management strategies for key subgroups of infants (e.g. for those for which resuscitation and/or stabilisation is deemed necessary, or extremely preterm infants) has led to 117 planned, ongoing or published trials (in more than 15,000 preterm babies) that are comparing a range of cord management strategies. *Individual participant data (IPD) meta-analysis* is the gold standard for combining such trial data. IPD provides larger statistical power for estimation of treatment effects of rarer secondary endpoints and enables reliable subgroup analyses to examine hypotheses about differences in treatment effect, exploring interactions between treatment- and participant-level characteristics. (42) A *network meta-analysis (NMA)* facilitates data synthesis when there are a range of interventions available and permits indirect comparisons across all interventions by inferring the relative effectiveness of two competing treatments through a common comparator.(43, 44) NMA produces relative effect

For peer review only - http://bmjopen.bmj.com/site/about/guidelines.xhtml

estimates for each intervention compared with every other intervention in the network. These effect sizes can be used to obtain rankings of the effectiveness of the interventions.(45) Using IPD in a NMA (as opposed to aggregate data) can improve precision, increase information, and reduce bias. (46)

Objectives

The aims of this study are:

1) to evaluate the effectiveness of cord management strategies for preterm infants on neonatal mortality and morbidity, and to evaluate patient-level modifiers of treatment effect.
2) to evaluate, compare and rank the effectiveness of different cord management strategies for preterm infants on mortality and the key secondary outcomes intraventricular haemorrhage (any grade) and infant blood transfusions (any).

METHODS AND ANALYSIS

We will conduct a systematic review of randomised trials with individual participant data using pairwise and network meta-analysis, and a nested prospective meta-analysis. The lead investigator for all potentially eligible studies will be contacted and invited to collaborate and join the **i**ndividual participant data **Co**rd **M**anagement at **P**reterm birth (iCOMP) Collaboration. Eligible trials identified up to February 2019 are listed in Supplementary File 1. The Collaboration will undertake this project according to the methods recommended by the Cochrane Individual Participant Data, Multiple Interventions, and Prospective Meta-Analysis Methods Groups.(42, 47, 48) This protocol is registered on the Australian New Zealand Clinical Trials Registry (ANZCTR, ACTRN12619001305112) and the International Prospective Register of Systematic Reviews (PROSPERO, CRD42019136640). Reporting guidelines for NMA protocols by Chaimani et al (49) and PRISMA extension for protocols (50, 51) have been followed for reporting (PRISMA-P checklist provided in Supplementary File 2).

Eligibility criteria

Types of studies

Studies will be included if they are randomised trials. Cluster-randomised and quasi-random studies will be excluded. Studies must compare at least two of the interventions of interest (defined below).

Trial participants

Participants will be women giving birth preterm (before 37 completed weeks' gestation) and/or their babies. Individually randomised studies will be eligible for inclusion if the unit of randomisation was either the woman, or the baby. Women and babies will be included regardless of whether mode of delivery was vaginal or caesarean, and whether the birth was singleton or multiple. Correlations between multiples will be accounted for in the analyses. Babies will be included regardless of whether or not they received immediate resuscitation at birth.

Types of interventions and comparators in pairwise meta-analysis

For peer review only - http://bmjopen.bmj.com/site/about/guidelines.xhtml

For peer review only

For the pairwise meta-analysis we will include all trials that compare an intervention to enhance umbilical blood flow or allow more time for physiological transition to the comparator immediate cord clamping. This includes interventions assessing cord management strategies for timing of cord clamping, and other strategies such as cord milking. Trials will be included regardless of whether initial neonatal care is provided with the umbilical cord intact, or not. Different strategies (i.e. deferred cord clamping and cord milking) will be analysed in separate subgroups to assess comparability between the groups by assessing subgroup effects and heterogeneity. They will then be collapsed into one "cord management intervention" group if they are deemed comparable based on the previous subgroup assessments. If they are deemed non-comparable they will be analysed and interpreted separately.

Types of interventions and comparators in network meta-analysis

For the network meta-analysis we will include, as interventions of interest, strategies for timing of cord clamping, and other cord management strategies to influence umbilical flow and placental transfusion.

Thus, interventions of interest include:

- Immediate cord clamping without milking (≤15 seconds or trialist defined)
- Short deferral of cord clamping (>15 to <45 seconds) without milking
- Medium deferral of cord clamping (≥45 to <90 seconds) without milking
- Long deferral of cord clamping (≥ 90 seconds) without milking
- Cord milking or stripping before immediate cord clamping (intact cord milking)
- Cord milking or stripping before deferred cord clamping (intact cord milking)
- Cord milking or stripping after immediate cord clamping (cut cord milking)
- Cord milking or stripping after deferred cord clamping (cut cord milking)
- Physiological clamping (clamping after aeration of lungs)

If we identify other interventions not listed above we will include them if they are addressing cord management or related strategies to influence umbilical flow and placental transfusion. Again, trials will be included regardless of whether initial neonatal care is provided with the umbilical cord intact, or not. Studies evaluating collection and storage of residual placental blood that is then used for transfusion after birth will be excluded. All possible comparisons between eligible interventions are displayed in Figure 1. For interpretation purposes, immediate cord clamping will act as the basis comparison/ parameter.

Nodes that specify different timings of cord clamping were defined according to what timing is classified as immediate clamping, short deferral, medium deferral or long deferral according to the literature to date (as shown in Supplementary File 1), and after discussion with clinicians. Different timings are commonly compared in head-to-head comparisons, hence, their classification as different intervention nodes. Similarly, nodes that specify cord milking were classified after a review of current milking techniques described in the literature and after discussion with clinicians. If insufficient data are available, categories will be collapsed where possible. For instance, milking before and after immediate cord clamping could be collapsed into one single immediate cord milking category, or medium and long delay could be collapsed into a medium to long delay category. We consider the interventions of interest to be jointly randomisable (i.e. each participant could, in principle, be randomised to any one of the interventions of interest).

For peer review only - http://bmjopen.bmj.com/site/about/guidelines.xhtml

Types of outcome measures

Trials must report at least one of the clinical outcomes included in this review, as specified in the "measures" section below, to be included.

Eligibility for nested prospective meta-analysis

Studies are only included in the nested prospective meta-analysis if the investigator/s were blind to outcome data by intervention group at the time the main components of the protocol (i.e. objectives, aims and hypotheses, eligibility criteria, subgroup and sensitivity analyses and main outcomes) were initially agreed in January 2015.

Information sources and search strategy

The search strategy to identify potentially eligible studies includes a search of the Cochrane Collaboration Pregnancy and Childbirth Review Group's Trial Register. This register contains trials identified from: monthly searches of the Cochrane Central Register of Controlled Trials (CENTRAL) and CINAHL (EBSCO); weekly searches of Medline (Ovid) and Embase (Ovid); hand searches of specialty journals and major conferences proceedings; and current awareness alerts from further journals and BioMed Central. Further details can be found elsewhere.(52) We will identify ongoing trials that may be eligible by searching for published protocols in Medline and Embase, searching online registries of clinical trials, and personal contacts (for example, by asking collaborators to notify any unregistered studies they are aware of). The Chief Investigators of eligible trials will be invited to join the iCOMP Collaboration. They will also be asked if they know of any further planned, ongoing or completed studies. Databases will be searched from their inception. Preliminary searches using this search strategy have already been completed up to 13 February 2019, but the search will be updated regularly to include additional trials. The search strategy is outlined in more detail in Supplementary File 3.

Selection of studies for inclusion in the review

Two members of the iCOMP Secretariat (see project management section below) will independently assess all the potentially eligible studies identified for inclusion. Disagreements will be resolved by discussion or, if required, by consulting a third member of the iCOMP Secretariat. Studies that are not willing or able to provide IPD will be synthesised where possible using aggregate data.

Data collection, management, and confidentiality

Data receipt

Each participating trial will be asked to provide de-identified, individual participant level data. Clear instructions will be provided on which data are needed and the secure data transfer process. The preferred data format and coding for each variable will be supplied to the investigators, but data in any format that is most convenient will be accepted and recoded if necessary. Data management will comply with the University of Sydney Data Management Policy 2014, and has been approved by the University of Sydney Human Research Ethics Committee (2018/886). Depending on trialists' preference, data transfer will either take place via secure data transfer platforms, or shared via

For peer review only - http://bmjopen.bmj.com/site/about/guidelines.xhtml

institutional secure email using password-protected zip-files. Data for this project will be stored in perpetuity in a password-protected folder within the NHMRC Clinical Trials Centre's network. Only authorised project team members working within the NHMRC Clinical Trials Centre will have access to these data.

Data processing

Data checking: For each trial, range and internal consistency of all variables will be checked. Intervention details and missing data will be checked against any protocols, published reports, and data collection sheets. Integrity of the randomisation process will be assessed by examining the chronological randomisation sequence and the balance of participant characteristics across intervention groups. Any inconsistencies or missing data will be discussed with the trialists and resolved by consensus. Each included study will be analysed separately and the results sent to the trial investigators for verification prior to inclusion in the iCOMP database. All trial-specific outcomes generated from the IPD will be cross-checked against published information via a series of crosstabs.

Data re-coding: Outcome data may have been collected in different formats across trials. Therefore, the de-identified data from each of the trials will be extracted and re-formatted into a commonly coded dataset.

Data transformation and collating: Once the data from each of the trials are finalised, they will be combined into a single dataset, but a trial identifier code for each participant will be retained. New variables will be generated from the combined dataset as required to address the hypotheses to be tested.

Risk of bias assessment and certainty of evidence appraisal

Eligible studies will be assessed for risk of bias using the criteria described in the Cochrane Handbook(53): random sequence generation; allocation concealment; blinding of participants and personnel; blinding of outcome assessment; incomplete outcome data; selective reporting; and other bias. Uncertainties will be resolved where possible by contacting study authors. Certainty of evidence will be appraised using the GRADE approach (54) for the pairwise comparisons, and the rating approach suggested by Salanti and colleagues that is implemented in the CINeMA application for the network meta-analysis.(55)

Outcomes measures for pairwise meta-analysis

All outcome measures for the pairwise meta-analysis are listed in Table 1. The primary outcome will be death of the baby prior to hospital discharge. As outcomes for babies born very preterm (before 32 weeks' gestation) are different to those born moderately preterm (32 to 37 weeks), separate analyses will be conducted for these two groups of infants for the secondary outcomes. Where possible, definitions will be standardised, otherwise outcomes will be used as defined in individual trials. Secondary outcomes will include measures of neonatal and maternal morbidity, and health service use.

Covariates and subgroups for pairwise meta-analysis

For peer review only - http://bmjopen.bmj.com/site/about/guidelines.xhtml

Subgroup analyses will be conducted for the primary outcome of death (prior to hospital discharge) and two key secondary outcomes (IVH any grade and any infant blood transfusion). All included covariates and subgroups are listed in Table 1. The comparative effects of alternative cord management strategies may vary depending on key infant risk factors, and/or on the level and type of neonatal care available at the hospital of birth. Thus, there will be subgroup analyses based on participant level characteristics and based on hospital level characteristics. If data are insufficient for the pre-specified subgroup analyses, categories will be collapsed.

Data analysis for pairwise meta-analysis

The full Statistical Analysis Plan will be agreed on by the iCOMP Collaboration before any analyses are undertaken. Analyses will include all randomised participants for which data are available, and the primary analyses will be based on intention-to-treat. Analyses will be conducted using the open-source software R.(56)

For each outcome, a one-stage approach to analysis will be employed to include individual participant data from all eligible trials in a multilevel random or mixed effects regression model. Aggregate data will be included where individual participant data are unavailable.(57) Relative heterogeneity of treatment effects across trials will be estimated using I^2, with further inclusion in secondary models of participant level and trial level covariates to explain the sources of heterogeneity. Prediction intervals will be estimated to ascertain absolute heterogeneity. Forest plots will be presented by trial for the primary outcome, and for any secondary outcomes where there is evidence of heterogeneity across trials.

We will use a generalised linear modelling framework, with the choice of outcome distribution and link function dependent on outcome type. For example, binomial with log link will be used to estimate risk ratios for binary outcomes, and Gaussian with identity link for mean differences, with log-transformation of the data if appropriate. We will follow a similar approach for secondary outcomes. For estimation of subgroup effects, we will present forest plots of pooled treatment effects according to pre-specified subgroup variables, and estimate effects by including appropriate interaction terms between a subgroup variable and treatment arm in the regression models. The results of all comparative analyses will be presented using appropriate estimates of treatment effect along with 95% confidence intervals and two-sided p-values.

Outcome measures for network meta-analysis

The primary outcome for the network meta-analysis will be death of the baby during the initial hospital stay. If data availability permits, IVH (any grade) and blood transfusion (any) will be analysed as two key secondary outcomes.

Covariates and subgroups for network meta-analysis

Subgroup analyses will be conducted for the primary outcome (death before discharge), and the two key secondary outcomes (IVH any grade, blood transfusion). Gestational week at birth and highest level of available care will be considered as effect modifiers to improve consistency of the NMA model. There will be subgroup analyses assessing treatment effect by week of gestational age, and by comparing babies assessed as in need of immediate resuscitation versus not in need of immediate resuscitation.

For peer review only - http://bmjopen.bmj.com/site/about/guidelines.xhtml

Assessment of the transitivity assumption for network meta-analysis

Transitivity in the network will be assured by only including interventions that are regarded as jointly randomisable and by limiting our sample to preterm infants. Gestational age at birth, hospital setting (highest level of available neonatal care), as well as study year may act as effect modifiers and could influence the transitivity of the network. We will therefore investigate whether these variables are distributed evenly across comparisons. If we find any of those variables to be unevenly distributed, they will be included in the network as covariates to investigate their influence on the network and on possible inconsistency.

Data analysis for network meta-analysis

As for the pairwise meta-analysis, all analyses will be specified a-priori in a full Statistical Analysis Plan, all randomised participants for which data are available will be included, and the primary analyses will be intention-to-treat. Again, aggregate data will be included where IPD are unavailable.

We will calculate a two-step random-effects contrast-based network meta-regression to compare and rank all available interventions for the primary outcome death (during initial hospital stay) and, , for the two key secondary outcomes - IVH (any grade) and blood transfusion (any). Summary risk ratios with confidence and prediction intervals will be presented for each pairwise comparison in a league table. We will estimate the ranking probability of each intervention being at each rank, and we will use surface under the cumulative ranking curve (SUCRA) and mean ranks to obtain a treatment hierarchy. A frequentist approach to analysis will be used. Should models not converge, a Bayesian approach will be used instead, setting a weakly-informative prior $d \sim$ N (0, 5). Correlations induced by multi-arm studies will be accounted for using multivariate distributions.

As a second step, interactions between key covariates and effect estimates will be tested, assuming a common interaction across comparisons. If there are statistically significant interactions between covariates and treatment effects, we will provide probability rankings of intervention effects by subgroup for these covariates. A single heterogeneity parameter will be assumed for each network. Statistical heterogeneity will be assessed using the I^2 statistic.

Assessment of inconsistency for network meta-analysis

Global consistency will be assessed using the Q statistics for inconsistency and the design-by-treatment interaction model. Local consistency will be assessed using the loop-specific approach and the node-splitting approach to explore sources of inconsistency. Since tests of inconsistency are known to have low power, results will be interpreted with caution, and potential known sources for inconsistency will be explored even if there is no statistical evidence of inconsistency. Any detected inconsistency will be explored by including covariates specified above (gestational age at birth, hospital setting, as well as study year) into the model, and by excluding potential outlier studies in sensitivity analyses. In case of a judgement of excessive heterogeneity or inconsistency we would still report the resulting parameters, but would interpret the results as not reliable.

Assessment of compliance with the allocated intervention

For peer review only - http://bmjopen.bmj.com/site/about/guidelines.xhtml

Compliance with the interventions will be described for each trial. For studies of early versus deferred cord clamping this will be based on i) the time to cord clamping in each allocated group, and ii) the difference in time between early and deferred clamping. For studies comparing cord milking with no milking, this will be based on i) time to cord clamping in the allocated groups, and ii) reported compliance with cord milking in both groups.

Assessment of selection bias

We will perform a nested prospective meta-analysis as a sensitivity analysis, to detect potential differences between prospectively and retrospectively included studies that may point to selection or publication bias. We expect to also be able to include some unreported outcomes sourced from the individual participant data provided by the included studies, which may alleviate selective outcome reporting bias.(58) Additionally, comparison-adjusted and contour-enhanced funnel plots(59) will be utilised to examine whether there are differences in results between more and less precise studies.

Adjustments for multiplicity

There is only one primary outcome, and few key secondary outcomes for this study. For other secondary outcomes, no formal adjustments for multiplicity (i.e. the accumulation of type 1 error and thus higher likelihood of chance findings when assessing multiple outcomes) are planned. Instead, we will be taking the following approach outlined by Schulz and Grimes(60): as secondary outcomes examined in this study are interrelated, we will interpret the pattern of results, examining consistency of results across related outcomes, instead of focusing on any single, statistically significant result. All secondary outcomes will be reported. Subgroup analyses will be performed by testing for interactions and findings will be reported as exploratory.(61)

Planned sensitivity analyses

To assess whether results are robust to trial characteristics and methods of analysis, the following sensitivity analyses will be conducted for the primary outcome, if data are sufficient:

- Excluding trials with high risk of bias for sequence generation and/or concealment of allocation and/or loss to follow up for pairwise and network meta-analysis;
- Excluding trials with a significant conflict of interest (e.g. trials funded by pharmaceutical companies)
- For trials comparing early cord clamping with deferred clamping, analysis of outcomes weighted by degree of separation in mean actual timing of cord clamping between intervention and control groups for pairwise meta-analysis;
- Analysis of outcomes weighted by degree of separation in haemoglobin (at 24 hours) achieved between intervention and control groups for pairwise meta-analysis (as a surrogate for net placental transfusion);
- For trials with deferred cord clamping, an additional dose-response analysis assessing intended time of cord clamping deferral as a continuous variable will be performed;
- Exploratory analysis based on actual, rather than intended, timing of cord clamping for individual participants for pairwise and network meta-analysis;

For peer review only - http://bmjopen.bmj.com/site/about/guidelines.xhtml

- The impact of missing data on the effects of the included interventions for the primary outcome may be explored (if appropriate).

Project management

The iCOMP Collaboration will invite membership from representatives of each of the included trials contributing individual participant data, have a Secretariat, and invite methodological and clinical experts who will form an Advisory Group. The Secretariat will be responsible for data collection, management and analysis, and for communication within the Collaboration.

Public and patient involvement

Two consumer representatives have been invited to join the iCOMP collaboration, comment on this protocol and be involved in the interpretation of results.

Ethical issues

For each included trial, ethics approval has been previously granted by their respective Human Research Ethics Committees (or equivalent), and informed consent has been obtained from all participants. The Chief Investigators of the included trials remain the custodians of their own trial's data. Individual participant data from the included trials will be de-identified before sharing with the iCOMP Collaboration. Ethics approval for this project has been granted by the University of Sydney Human Research Ethics Committee (number: 2018/886).

Publication policy

The key methods for this meta-analysis protocol were agreed by the iCOMP Collaborators in January 2015, before unblinding of any outcome data from the studies included in the nested prospective meta-analysis. This manuscript was discussed at the iCOMP Collaborators' meeting held at the Pediatric Academic Societies meeting in San Diego in April 2015. At this meeting it was agreed the protocol should be expanded to include a retrospective systematic review and individual participant data and network meta-analysis with a nested prospective meta-analysis. The protocol was then revised, based on further discussion, and circulated to members of the iCOMP Collaboration for further comment and agreement prior to manuscript submission.

Participating trialists in the prospective meta-analysis, when reporting results from their own trials, will endeavour to include a statement that their trial is part of this prospective meta-analysis in any published manuscripts or conference abstracts. Any reports of the results of this meta-analysis will be published either in the name of the collaborative group, or by representatives of the collaborative group on behalf of the iCOMP Collaboration, as agreed by members of the collaborative group. Draft reports will be circulated to the collaborative group for comment and approval before submission for publication.

For peer review only

For peer review only - http://bmjopen.bmj.com/site/about/guidelines.xhtml

DISCUSSION

There is an urgency to conduct this systematic review and pairwise individual participant data and network meta-analysis so we can make sense of the numerous trials currently being undertaken, inform clinical practice, and identify the most promising interventions for further evaluation.

This meta-analysis offers an opportunity to reliably test important hypotheses that cannot be resolved by any of the individual trials, either alone or in simple combination. Coordinating international efforts in this way will help achieve consensus on the most important substantive clinical outcomes to assess in any future trials as needed. Unequivocal synthesised results, together with the identification of key determinants (e.g. effect modifiers), will be critical for translating evidence from this meta-analysis directly into practice. Figure 2 shows the network of comparisons available from the trials identified to date. We plan to complete study identification and individual participant data collection by early-2020, then conduct the analyses and disseminate results by mid-2021. Trials that are ongoing and therefore unable to provide data by March 2020 will remain members of the iCOMP collaboration. Their data will be included in future updates of iCOMP.

This study is only possible because trialists around the world have agreed to collaborate to share the individual participant data from their cord management trials. This collaborative approach will enable us to move beyond the traditional “one-size-fits-all” and towards precision medicine, to find the optimal intervention from a range of treatment options for each individual woman and her baby, based on their individual characteristics and risk factors.

Acknowledgements

Thanks to Sarah Somerset, Min Yang, Charlotte Lloyd, and Virginia Portillo for previous support for the Secretariat and input into earlier protocol drafts.

Competing interests

None known.

Non-financial competing interests

Lelia Duley, Anup Katheria, Catalina De Paco Matallana, Eugene Dempsey, Heike Rabe, John Kattwinkel, Judith Mercer, Justin Josephsen, Karen Fairchild, Ola Andersson, Shigeharu Hosono, Venkataseshan Sundaram, Vikram Datta, Walid El-Naggar, and William Tarnow-Mordi are Chief Investigators for potentially eligible trials.

Funding

Developing the protocol and establishing the collaborative group was supported by the UK National Institute of Health Research with a grant entitled “The Preterm Birth Programme” (number RPPG060910107). This grant presents independent research commissioned by the National Institute for Health Research (NIHR) under its Programme Grants for Applied Research funding scheme (RP-PG-0609-10107). The views expressed are those of the authors and not necessarily those of the NHS, the NIHR or the Department of Health. Funding for individual trials remains the responsibility of the trialists

For peer review only - http://bmjopen.bmj.com/site/about/guidelines.xhtml

For peer review only

themselves. Funding to undertake data collection and data analysis for the iCOMP Collaboration has been provided by the Australian National Health and Medical Research Council via a Project Grant (APP1163585).

Author contributions

Duley and Askie conceived the idea. Seidler, Askie, and Duley drafted the protocol. All remaining authors (Katheria, De Paco Matallana, Dempsey, Rabe, Kattwinkel, Mercer, Josephsen, Fairchild, Andersson, Hosono, Sundaram, Datta, El-Naggar, Tarnow-Mordi, Debray, Hooper, Kluckow, Polglase, Davis, Montgomery, Hunter, Barba and Simes) critically revised all drafts of the manuscript for intellectual content, and agreed and approved the final manuscript. Seidler is the guarantor of the review.

List of abbreviations

iCOMP – **i**ndividual participant data on **Co**rd **M**anagement at **P**reterm birth

CENTRAL – Cochrane Central Register of Controlled Trials

CINeMA – Confidence in Network meta-analysis

CPAP – continuous positive airway pressure

IPD – individual participant data

NICE – National Institute for Health and Care Excellence

NICU – neonatal intensive care unit

NIHR – National Institute for Health Research

NMA – network meta-analysis

PRISMA – Preferred Reporting Items for Systematic Review and Meta-Analysis

UK – United Kingdom

WHO – World Health Organization

ILCOR – International Liaison Committee on Resuscitation

PROSPERO – International prospective register of systematic reviews

For peer review only

For peer review only - http://bmjopen.bmj.com/site/about/guidelines.xhtml

iCOMP collaboration members:

Secretariat: Anna Lene Seidler[1], Lelia Duley[2], Alan Montgomery[2], Kylie E Hunter[1], Angie Barba[1], Ava Grace Tan-Koay[1], Lisa Askie[1]

Trialists: Amir Kugelman[3], Anu George[4], Anu Sachdeva[5], Anup Katheria[6], Arjan Te Pas[7], Ashish Kc[8], Bimlesh Kumar[9], Carl Backes[10], Catalina De Paco Matallana[11], Chamnan Tanprasertkul[12], Chayatat Ruangkit[13], Eugene Dempsey[14], G Ram Mohan[15], Gillian Gyte[16], Guillermo Carroli[17], Heidi Al-Wassia[18], Hytham Atia[19], Heike Rabe[20], Islam Nour[21], Jiangqin Liu[22], John Bauer[23], John Kattwinkel[24], Judith Mercer[25], Justin Josephsen[26], Karen Fairchild[24], Kellie Murphy[27], Kristy Robledo[1], Lakhbir Dhaliwal[15], Laura Perretta[28], Lin Ling[29], Manoj Varanattu[30], Maria Goya[31], Michael Meyer[32], Musa Silahli[33], Neelam Kler[34], Neil Finer[35], Ola Andersson[8], Omar Kamlin[36], Peter Giannone[37], Pharuhad Pongmee[38], Prisana Panichkul[39], Ronny Knol[40], Sandeep Kadam[41], Sangkae Chamnanvanakij[39], Shigeharu Hosono[42], Shiraz Badurdeen[36], Simone Pratesi[43], Thomas Ranjit[44], Venkataseshan Sundaram[15], Victor Lago Leal[45], Vikram Datta[46], Waldemar Carlo[47], Walid El-Naggar[48] &William Tarnow-Mordi[1].

Advisers: Graeme Polglase[49], John Simes[1], Martin Kluckow[49], Peter Davis[336], Stuart Hooper[49], Thomas P.A. Debray[50].

[1] National Health and Medical Research Council Clinical Trials Centre, University of Sydney, Sydney, Australia
[2] Nottingham Clinical Trials Unit, University of Nottingham, Nottingham, UK
[3] Bnai Zion Medical Center, Haifa, Israel
[4] Malankara Orthodox Syrian Church Medical College, Kerala, India
[5] All India Institute of Medical Science, New Delhi, India
[6] Sharp Healthcare, San Diego, California, USA
[7] Leiden University, Leiden, Netherlands
[8] Uppsala University, Uppsala, Sweden
[9] Lala Lajpat Rai Memorial Medical College, Uttar Pradash, India
[10] Ohio State University Wexner Medical Center, Columbus, Ohio, USA
[11] Clinic University Hospital Virgen de la Arrixaca, Murcia, Spain
[12] Thammasat University, Pathumthani, Thailand
[13] Mahidol University, Samut Prakan, Thailand
[14] Cork University Maternity Hospital, Cork, Ireland
[15] Post Graduate Institute of Medical Education & Research, Chandigarh, India
[16]Oldfield, Poulton le Fylde, UK
[17] Rosarino Center for Perinatal Studies, Rosario, Argentina
[18] King Abdulaziz University, Jeddah, Saudi Arabia
[19] Zagazig University, Zagazig, Egypt
[20] Brighton and Sussex Medical School, University of Sussex, Brighton, UK
[21] Mansoura University Children's Hospital, Mansoura, Egypt
[22] Tongji University School of Medicine, Shanghai, China
[23] University of Kentucky, Lexington, Kentucky, USA
[24] University of Virginia, Charlottesville, Virginia, USA

For peer review only - http://bmjopen.bmj.com/site/about/guidelines.xhtml

[25] The University of Rhode Island, Kingston, Rhode Island, USA
[26] St Louis University School of Medicine, St. Louis, Missouri, USA
[27] Mount Sinai Hospital, Toronto, Canada
[28] Weill Cornell Medical College, New York City, New York, USA
[29] Suining Central Hospital, Sichuan, China
[30] Jubilee Mission Medical College & Research Centre, Kerala, India
[31] Vall d'Hebron University Hospital, Barcelona, Spain
[32] Middlemore Hospital, Auckland, New Zealand
[33] Baskent University, Ankara, Turkey
[34] Sir Ganga Ram Hospital, New Delhi, India
[35] University of California, San Diego, California, USA
[36] The Royal Women's Hospital, Melbourne, Australia
[37] Ramathibodi Hospital, Mahidol University, Bangkok, Thailand
[38] Phramongkutklao Hospital, Ratchathewi, Bangkok, Thailand
[39] Erasmus MC, University Medical Centre, Rotterdam, Netherlands
[40] KEM Hospital, Pune, Maharashtra, India
[41] Jichi Medical University Saitama Medical Center, Saitama, Japan
[42] University of Florence, Florence, Italy
[43] St John's Medical College & Hospital, Bangalore, Kamataka, India
[44] University Hospital of Getafe & European University of Madrid, Madrid, Spain
[45] Lady Hardinge Medical College, New Delhi, India
[46] University of Alabama at Birmingham, Birmingham, Alabama, USA
[47] Department of Pediatrics, Dalhousie University, Halifax, Canada
[48] The Ritchie Centre, Obstetrics & Gynaecology, Monash University, Melbourne, Australia
[49] Faculty of Medicine and Health, University of Sydney, Sydney, Australia
[50] Julius Center for Health Sciences and Primary Care, University Medical Center Utrecht, Utrecht, the Netherlands

For peer review only

For peer review only - http://bmjopen.bmj.com/site/about/guidelines.xhtml

References

1. Blencowe H, Cousens S, Oestergaard MZ, Chou D, Moller AB, Narwal R, et al. National, regional, and worldwide estimates of preterm birth rates in the year 2010 with time trends since 1990 for selected countries: a systematic analysis and implications. Lancet. 2012;379(9832):2162-72.
2. March of Dimes P, Save the Children, WHO. Born too soon: the global action report on preterm birth. Geneva: World Health Organisation; 2012.
3. Chang HH, Larson J, Blencowe H, Spong CY, Howson CP, Cairns-Smith S, et al. Preventing preterm births: analysis of trends and potential reductions with interventions in 39 countries with very high human development index. Lancet. 2013;381(9862):223-34.
4. Petrou S, Mehta Z, Hockley C, Cook-Mozaffari P, Henderson J, Goldacre M. The impact of preterm birth on hospital inpatient admissions and costs during the first 5 years of life. Pediatrics. 2003;112(6 Pt 1):1290-7.
5. Mangham LJ, Petrou S, Doyle LW, Draper ES, Marlow N. The cost of preterm birth throughout childhood in England and Wales. Pediatrics. 2009;123(2):e312-27.
6. Zeitlin J, Draper ES, Kollee L, Milligan D, Boerch K, Agostino R, et al. Differences in rates and short-term outcome of live births before 32 weeks of gestation in Europe in 2003: results from the MOSAIC cohort. Pediatrics. 2008;121(4):e936-44.
7. Bhutta AT, Cleves MA, Casey PH, Cradock MM, Anand KJS. Cognitive and Behavioral Outcomes of School-Aged Children Who Were Born Preterm: A Meta-analysis. JAMA. 2002;288(6):728-37.
8. Aarnoudse-Moens CS, Weisglas-Kuperus N, van Goudoever JB, Oosterlaan J. Meta-analysis of neurobehavioral outcomes in very preterm and/or very low birth weight children. Pediatrics. 2009;124(2):717-28.
9. Anderson P, Doyle LW. Neurobehavioral outcomes of school-age children born extremely low birth weight or very preterm in the 1990s. JAMA. 2003;289(24):3264-72.
10. International Liaison Committee on Resuscitation (ILCOR). Neonatal Resuscitation- Part 7. 2015 International Consensus on Cardiopulmonary Resuscitation and Emergency Cardiovascular Care Science with Treatment Recommendations. In: Perlman JM, Wyllie J & Kattwinkel et al. Circulation. 2015;132(suppl 1):S204-S41.
11. Farrar D, Airey R, Law GR, Tuffnell D, Cattle B, Duley L. Measuring placental transfusion for term births: weighing babies with cord intact. BJOG. 2011;118(1):70-5.
12. Boere I, Roest A, E W, ten Harkel A, Haak M, Morley C, et al. Umbilical blood flow patterns directly after birth before delayed cord clamping. Arch Dis Child Fetal Neonatal Ed. 2015;100:F121–F125. doi:10.1136/archdischild-2014-307144.
13. Yao A, Moinian M, Lind J. Distribution of blood between infant and placenta after birth. The Lancet. 1969;294(7626):871-3.
14. Saigal S, O'Neill A, Surainder Y, Chua LB, Usher R. Placental transfusion and hyperbilirubinemia in the premature. Pediatrics. 1972;49(3):406-19.
15. Dawes GS. Foetal and neonatal physiology; a comparative study of the changes at birth [by] Geoffrey S. Dawes. Chapter 13: Chicago, Year Book Medical Publishers; 1968. 247 p. illus. 27 cm. p.
16. Fogarty M, Osborn DA, Askie L, Seidler AL, Hunter K, Lui K, et al. Delayed vs early umbilical cord clamping for preterm infants: a systematic review and meta-analysis. American Journal of Obstetrics and Gynecology. 2018;218(1):1-18.
17. Vijayaselvi R, Abraham A, Kumar M, Kuruvilla A, Mathews J, Duley L. Measuring umbilical flow and placental transfusion for preterm births: weighing babies at 33-36 weeks gestation with cord intact. 1st congress of joint European Neonatal Societies. 2015.
18. Committee Opinion No. 684: Delayed Umbilical Cord Clamping After Birth. Obstetrics & Gynecology. 2017;129(1):e5-e10.
19. Schoonakker B, . Dorling, J,. Oddie, S,. Batra, D,. Grace, N,. Duley, L, editor Bedside resuscitation of preterm infants with cord intact is achievable using standard resuscitaire. Available at

For peer review only - http://bmjopen.bmj.com/site/about/guidelines.xhtml

p430 of https://www.eiseverywhere.com/file_uploads/9206db9fe962868d47f709b38365ec5e_9349_abstract_book_-_25sett13-it-it.pdf. European Society for Pediatric Research; 2013; Oporto, Portugal.
20. Thomas M, Yoxall C, Weeks A, Duley L. Providing newborn resuscitation at the mother's bedside: assessing the safety, usability and acceptability of a mobile trolley. BMC Pediatrics. 2014;14:135.
21. Duley L, Dorling J, Pushpa-Rajah A, Oddie SJ, Yoxall CW, Schoonakker B, et al. Randomised trial of cord clamping and initial stabilisation at very preterm birth. Archives of Disease in Childhood - Fetal and Neonatal Edition. 2018;103(1):F6-F14.
22. Sawyer A, Ayers S, Bertullies S, Thomas M, Weeks AD, Yoxall CW, et al. Providing immediate neonatal care and resuscitation at birth beside the mother: parents' views, a qualitative study. BMJ Open. 2015;5(9).
23. Yoxall CW, Ayers S, Sawyer A, Bertullies S, Thomas M, D Weeks A, et al. Providing immediate neonatal care and resuscitation at birth beside the mother: clinicians' views, a qualitative study. BMJ Open. 2015;5(9).
24. Schoonakker B, . Dorling, J,. Oddie, S,. Batra, D,. Grace, N,. Duley, L. Bedside resuscitation of preterm infants with cord intact is achievable using standard resuscitaire. Available at p430 of https://www.eiseverywhere.com/file_uploads/9206db9fe962868d47f709b38365ec5e_9349_abstract_book_-_25sett13-it-it.pdf. European Society for Pediatric Research. 2013.
25. Kluckow M, Hooper SB. Using physiology to guide time to cord clamping. Seminars in Fetal and Neonatal Medicine. 2015;20(4):225-31.
26. Bhatt S, Alison BJ, Wallace EM, Crossley KJ, Gill AW, Kluckow M, et al. Delaying cord clamping until ventilation onset improves cardiovascular function at birth in preterm lambs. The Journal of physiology. 2013;591(8):2113-26.
27. Ersdal HL, Linde J, Mduma E, Auestad B, J P. Neonatal outcome following cord clamping after onset of spontaneous respiration. Pediatrics. 2014;134:265-72.
28. Nevill E, MP M. Effect of delayed cord clamping (DCC) on breathing and transition at birth in very preterm infants. Early Hum Dev 2015;91:407-11.
29. Polglase GR, Dawson JA, Kluckow M, Gill AW, Davis PG, te Pas AB, et al. Ventilation Onset Prior to Umbilical Cord Clamping (Physiological-Based Cord Clamping) Improves Systemic and Cerebral Oxygenation in Preterm Lambs. PLOS ONE. 2015;10(2):e0117504.
30. Polglase GR, Blank DA, Barton SK, Miller SL, Stojanovska V, Kluckow M, et al. Physiologically based cord clamping stabilises cardiac output and reduces cerebrovascular injury in asphyxiated near-term lambs. Arch Dis Child Fetal Neonatal Ed. 2017:https://doi.org/10.1136/archdischild-2017-313657 [Epub ahead of print].
31. Katheria AC, Brown MK, Faksh A, Hassen KO, Rich W, Lazarus D, et al. Delayed Cord Clamping in Newborns Born at Term at Risk for Resuscitation: A Feasibility Randomized Clinical Trial. The Journal of Pediatrics. 2017;187:313-7.e1.
32. Katheria A, Poeltler D, Durham J, Steen J, Rich W, Arnell K, et al. Neonatal Resuscitation with an Intact Cord: A Randomized Clinical Trial. Journal of Pediatrics. 2016;178:75-80.e3.
33. Hosono S, Mugishima H, Fujita H, Hosono A, Minato M, Okada T, et al. Umbilical cord milking reduces the need for red cell transfusions and improves neonatal adaptation in infants born at less than 29 weeks' gestation: a randomised controlled trial. Arch Dis Child Fetal Neonatal Ed. 2008;93(1):F14-9.
34. Blank DA, Polglase GR, Kluckow M, Gill AW, Crossley KJ, Moxham A, et al. Haemodynamic effects of umbilical cord milking in premature sheep during the neonatal transition. Archives of Disease in Childhood - Fetal and Neonatal Edition. 2017.
35. Katheria AC, Reister F, Hummler H, Essers J, Mendler M, Truong G, et al. LB 1: Premature Infants Receiving Cord Milking or Delayed Cord Clamping: A Randomized Controlled Non-inferiority Trial. American Journal of Obstetrics & Gynecology. 2019;220(1):S682.

For peer review only - http://bmjopen.bmj.com/site/about/guidelines.xhtml

36. WHO. WHO Recommendations for the Prevention and Treatment of Postpartum Haemorrhage. Geneva: World Health Organization. 2012.
37. National Institute for Health and Care Excellence. Preterm labour and birth. London; 2015.
38. European Resuscitation Council Guidelines for Resuscitation 2015 Section 7. Resuscitation and support of transition of babies at birth.
39. World Health Organisation. Guideline: delayed umbilical cord clamping for improved maternal and infant health and nutrition outcomes. Geneva, Switzerland; 2014.
40. Rabe H, Diaz-Rossello JL, Duley L, Dowswell T. Effect of timing of umbilical cord clamping and other strategies to influence placental transfusion at preterm birth on maternal and infant outcomes. Cochrane Database Syst Rev. 2012;8:CD003248.
41. Hosono S, Mugishima H, Fujita H, Hosono A, Minato M, Okada T, et al. Umbilical cord milking reduces the need for red cell transfusions and improves neonatal adaptation in infants born at less than 29 weeks' gestation: a randomised controlled trial. Arch Dis Child Fetal Neonatal Ed. 2008;93(1):F14-9.
42. Stewart LA, Tierney JF, Clarke M, on behalf of the Cochrane Individual Patient Data Meta-analysis Methods Group. Chapter 18: Reviews of individual patient data. In: Higgins J, Green S, editors. Cochrane Handbook for Systematic Reviews of Interventions: Version 5.1.0 [updated March 2011]. The Cochrane Collaboration, 2011. Available: www.cochrane-handbook.org.
43. Caldwell DM, Ades AE, Higgins JP. Simultaneous comparison of multiple treatments: combining direct and indirect evidence. Bmj. 2005;331(7521):897-900.
44. Lumley T. Network meta-analysis for indirect treatment comparisons. Statistics in medicine. 2002;21(16):2313-24.
45. Sutton A, Ades AE, Cooper N, Abrams K. Use of indirect and mixed treatment comparisons for technology assessment. PharmacoEconomics. 2008;26(9):753-67.
46. Debray TP, Schuit E, Efthimiou O, Reitsma JB, Ioannidis JP, Salanti G, et al. An overview of methods for network meta-analysis using individual participant data: when do benefits arise? Statistical methods in medical research. 2018;27(5):1351-64.
47. Ghersi D, Berlin J, Askie L. Chapter 19: Prospective meta-analysis. In: Higgins J, Green S, editors. Cochrane Handbook for Systematic Reviews of Interventions: Version 5.1.0 [updated March 2011]. The Cochrane Collaboration, 2011. Available: www.cochrane-handbook.org.
48. Higgins J, Deeks J, Altman D. Ch. 16.6: Indirect comparisons and multiple-treatments meta-analysis. Cochrane Handbook for Systematic Reviews of Interventions: Cochrane Collaboration; 2011.
49. Chaimani A, Caldwell DM, Li T, Higgins JP, Salanti G. Additional considerations are required when preparing a protocol for a systematic review with multiple interventions. Journal of clinical epidemiology. 2017;83:65-74.
50. Moher D, Shamseer L, Clarke M, Ghersi D, Liberati A, Petticrew M, et al. Preferred reporting items for systematic review and meta-analysis protocols (PRISMA-P) 2015 statement. Systematic Reviews. 2015;4(1):1.
51. Stewart LA, Clarke M, Rovers M, Riley RD, Simmonds M, Stewart G, et al. Preferred Reporting Items for a Systematic Review and Meta-analysis of Individual Participant Data: The PRISMA-IPD StatementPRISMA Extension for Individual Patient DataPRISMA Extension for Individual Patient Data. JAMA. 2015;313(16):1657-65.
52. CPaCG. Detailed search methods used to maintain and update the Specialised Register [Available from: https://pregnancy.cochrane.org/sites/pregnancy.cochrane.org/files/public/uploads/Cochrane%20Pregnancy%20and%20Childbirth%20Group%20search%20methods%20for%20Intervention%20reviews_0.pdf last accessed July 31, 2018.
53. Higgins J, Green S. Cochrane handbook for systematic reviews of interventions. Oxford: Wiley-Blackwell; 2008.

For peer review only - http://bmjopen.bmj.com/site/about/guidelines.xhtml

54. Guyatt G, Oxman AD, Akl EA, Kunz R, Vist G, Brozek J, et al. GRADE guidelines: 1. Introduction—GRADE evidence profiles and summary of findings tables. Journal of clinical epidemiology. 2011;64(4):383-94.
55. Salanti G, Higgins JP, Ades A, Ioannidis JP. Evaluation of networks of randomized trials. Statistical Methods in Medical Research. 2008;17(3):279-301.
56. Bhatt S, Alison BJ, Wallace EM, Crossley KJ, Gill AW, Kluckow M, et al. Delaying cord clamping until ventilation onset improves cardiovascular function at birth in preterm lambs. The Journal of physiology. 2013;591(8):2113-26.
57. Riley RD, Steyerberg EW. Meta-analysis of a binary outcome using individual participant data and aggregate data. Research Synthesis Methods. 2010;1(1):2-19.
58. Tierney JF, Stewart LA, Clarke M, on behalf of the Cochrane Individual Participant Data Meta-analysis Methods Group. Chapter 26: Individual participant data. In: Higgins JPT TJ, Chandler J, Cumpston M, Li T, Page MJ, Welch VA, editor. Cochrane Handbook for Systematic Reviews of Interventions version 60 (updated July 2019). Cochrane, 2019. Available from www.training.cochrane.org/handbook. [last accessed 15 January 2020].
59. Peters JL, Sutton AJ, Jones DR, Abrams KR, Rushton L. Contour-enhanced meta-analysis funnel plots help distinguish publication bias from other causes of asymmetry. Journal of clinical epidemiology. 2008;61(10):991-6.
60. Schulz KF, Grimes DA. Multiplicity in randomised trials I: endpoints and treatments. Lancet (London, England). 2005;365(9470):1591-5.
61. Schulz KF, Grimes DA. Multiplicity in randomised trials II: subgroup and interim analyses. Lancet (London, England). 2005;365(9471):1657-61.

For peer review only - http://bmjopen.bmj.com/site/about/guidelines.xhtml

Figure headings

Figure 1. Network of possible comparisons between cord management interventions

Figure 2. Illustration of network of currently available trials comparing different timing of cord clamping.

For peer review only

For peer review only - http://bmjopen.bmj.com/site/about/guidelines.xhtml

Table 1. Measures for individual participant data pairwise meta-analysis

**Outcomes**	
For all infants	
Primary outcome	• Death prior to hospital discharge
For infants born before 32 weeks' gestation	
Key secondary outcomes	• Death (at any time during duration of follow-up)<br>• Severe intraventricular haemorrhage on cranial ultrasound (grade 3-4)<br>• All grades of intraventricular haemorrhage on cranial ultrasound<br>• Necrotizing enterocolitis ≥ grade 2 (or trialist definition)<br>• Late onset sepsis (where possible defined as clinical sepsis > 72 hour after birth)<br>• Patent ductus arteriosus requiring treatment (medical and/or surgical)<br>• Chronic lung disease (at 36 weeks' postmenstrual age or trialist defined)<br>• Blood transfusion (yes/no)
Other secondary outcomes	• Death (within 7 days)<br>• Other forms of white matter brain injury (e.g. periventricular leukomalacia, porencephaly)<br>• Respiratory support (mechanical ventilation, CPAP, low nasal flow oxygen)<br>• Duration of respiratory support<br>• Supplemental oxygen at 36 weeks<br>• Retinopathy of prematurity requiring treatment (medical and/or surgical)<br>• Drug treatment for hypotension (yes/no)<br>• Blood transfusion (number, volume)<br>• Hypothermia on admission to neonatal unit<br>• Haemoglobin<br>• Haematocrit<br>• Polycythaemia<br>• Jaundice requiring treatment<br>• Birthweight<br>• Length of stay in Neonatal Intensive Care Unit (NICU)/ Special Care Unit (SCU)<br>• Length of stay in hospital<br>• Apgar scores at 1 and 5 minutes<br>• Long term developmental disability (assessed using the Bayley III, and/or other tools):<br>○ cerebral palsy<br>○ neurodevelopmental disability<br>○ score on cognitive scale<br>○ score on language scale<br>○ score on social/emotional scale<br>○ score on motor scale<br>○ score on behaviour scale<br>○ deafness<br>○ blindness
For infants born at or after 32 weeks' gestation	

For peer review only

For peer review only - http://bmjopen.bmj.com/site/about/guidelines.xhtml

Key secondary outcomes	• Death at any time (during duration of follow-up)<br>• Admission to NICU<br>• Blood transfusion (any, number, volume)
Other secondary outcomes	• Death (within 7 days)<br>• Haemoglobin<br>• Haematocrit<br>• Jaundice requiring treatment<br>• Length of stay in NICU/SCU<br>• Length of stay in hospital<br>• Duration of respiratory support (mechanical ventilation, CPAP, low flow nasal oxygen)<br>• Chronic lung disease<br>• Late onset sepsis (> 72 hour after birth)<br>• Patent ductus arteriosus requiring treatment (medical and/or surgical)<br>• Drug treatment for hypotension<br>• Hypothermia on admission to neonatal unit or postnatal ward<br>• Apgar score at 1 and 5 minutes<br>• Long term developmental disability (assessed using the Bayley III, and/or other tools):<br>○ cerebral palsy<br>○ neurodevelopmental disability<br>○ score on cognitive scale<br>○ score on language scale<br>○ score on social/emotional scale<br>○ score on motor scale<br>○ score on behaviour scale<br>○ deafness<br>○ blindness
For all women	
Secondary outcomes	• Maternal death<br>• Postpartum haemorrhage<br>• Postnatal sepsis requiring treatment<br>• Manual removal of placenta<br>• Retained placenta<br>• Not breast feeding when baby discharged from hospital<br>• Postnatal depression<br>• Blood transfusion
**Covariates/ Subgroups**	
Participant-level characteristics	
	• Gestation at birth<br>• Type of pregnancy: singleton; multiple<br>• Maternal age<br>• Mode of birth: caesarean before onset of labour; caesarean after onset of labour; vaginal<br>• Onset of labour: spontaneous onset or spontaneous prelabour ruptured membranes; not spontaneous onset or spontaneous prelabour ruptured membranes; not known whether spontaneous onset of labour or spontaneous prelabour ruptured membranes

For peer review only - http://bmjopen.bmj.com/site/about/guidelines.xhtml

	• Type of breathing onset: spontaneous breathing onset; supported lung aeration (ventilation); unknown<br>• Time of breathing onset relative to cord clamping: before cord clamping/milking; after cord clamping/milking; unknown<br>• Sex (male, female, uncertain/other)<br>• Ethnicity (trialist defined)<br>• Small for gestational age (trialist defined): yes/no<br>• Maternal antenatal/intrapartum sepsis requiring treatment (trialist defined): yes/no<br>• Assessed as needing resuscitation and/or stabilisation (yes/no)<br>• Type of uterotonic drug (if any)
Hospital / trial-level characteristics	
	• Highest level of neonatal unit available at site: neonatal intensive care unit, neonatal unit (some capacity to provide ventilation), special care baby unit (no ventilation available), no neonatal unit or special care baby unit<br>• Planned timing of uterotonic drug: before cord clamping; after/at cord clamping; timing mixed or not known<br>• Planned position of the baby relative to the placenta whilst cord intact: level with placenta (between level of woman's bed and her abdomen/anterior thigh); more than 20 cm below level of placenta; position mixed or not known<br>• Need for immediate resuscitation at birth: infants requiring immediate resuscitation at birth excluded; infants requiring immediate resuscitation at birth included; unclear whether infants requiring immediate resuscitation at birth included or excluded<br>• Type of consent waiver of consent: deferred consent; informed consent or assent; type of consent unclear<br>• Study year

For peer review only

For peer review only - http://bmjopen.bmj.com/site/about/guidelines.xhtml

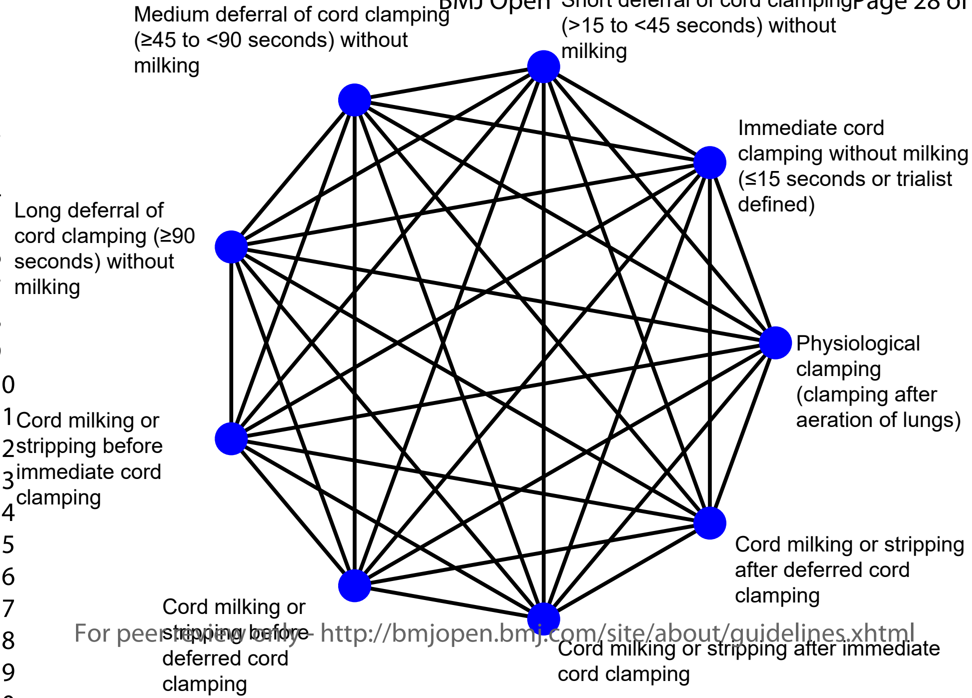

For peer review only - http://bmjopen.bmj.com/site/about/guidelines.xhtml

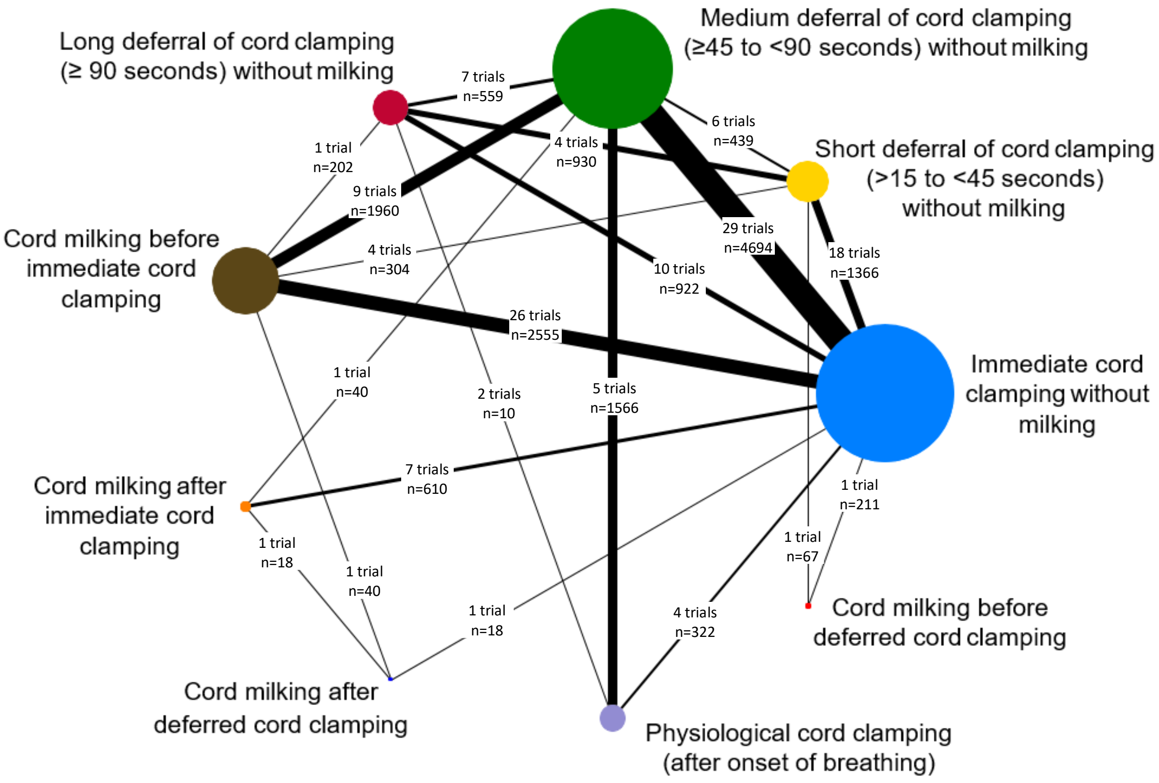

Long deferral of cord clamping (≥ 90 seconds) without milking
Medium deferral of cord clamping (≥45 to <90 seconds) without milking
Short deferral of cord clamping (>15 to <45 seconds) without milking
Cord milking before immediate cord clamping
Immediate cord clamping without milking
Cord milking after immediate cord clamping
Cord milking before deferred cord clamping
Cord milking after deferred cord clamping
Physiological cord clamping (after onset of breathing)
7 trials n=559
6 trials n=439
1 trial n=202
4 trials n=930
9 trials n=1960
29 trials n=4694
18 trials n=1366
4 trials n=304
10 trials n=922
26 trials n=2555
1 trial n=40
2 trials n=10
5 trials n=1566
7 trials n=610
1 trial n=211
1 trial n=67
1 trial n=18
1 trial n=40
1 trial n=18
4 trials n=322

review only

iCOMP Trial Master List January 2020

This table shows all trials that we have identified as being eligible for inclusion in iCOMP. All trials that are part of the iCOMP collaboration have been marked in blue.

Table: Eligible randomised trials to date for the pairwise & network meta-analysis with individual participant data on Cord Management at Preterm Birth (iCOMP)

Trial Country (PI)	Publication year	Start year/ completion year	Sample size	Participants	Intervention	Comparator	Primary outcome/s
Argentina [1] (Carroli)	n/a	2016/2020	700	Singletons, 24-30[6] weeks' GA	DCC: at 90 sec	DCC: ~30 sec	Sepsis (proven and very probable)
Australia [2] (Badurdeen)	n/a	2018/2020	120 (not all preterm)	≥32 weeks' GA*, require resuscitation at delivery	DCC: resuscitation prior to cord clamping (for PPV, clamping delayed until at least 60 sec after colour change of pedicap/neostat; for CPAP, clamping occurs at least 2 min after delivery)	ICC (followed by resuscitation)	Average heart rate between 60-120 sec after birth
Australia [3] (McDonnell)	1997	1994/1994	46	26 to 33 weeks' GA	DCC: 30 sec	ICC	Venous haematocrit
Australia [4] (Kamlin)	n/a	2014/2015	27 (not all preterm)	32-42 weeks' GA*	Arm 1: DCC at 90-180 sec<br>Arm 2: DCC 10 sec after crying and breathing established	DCC: <60 sec	Heart rate 90 sec after birth
Australia [5] (Tarnow-Mordi 2009)	n/a (Pilot for Tarnow-Mordi 2017)	2009/2010	100	<32 weeks' GA	Arm 1: Cord milking during resuscitation - cord cut long (3cm from placenta/ introitus)<br>Arm 2: DCC at 30-60 sec. If baby in extremis, immediate clamping.	ICC: within 10 sec	Haemoglobin 6 hours after birth

iCOMP

Trial Country (PI)	Publication year	Start year/ completion year	Sample size	Participants	Intervention	Comparator	Primary outcome/s
					Arm 3: DCC at 30-60 sec + milking		
Australia [6] (Tarnow-Mordi 2017)	2017	2010/2017	1634	<30 weeks' GA	DCC: ≥60 sec	ICC within 10 sec	Composite: Death or major morbidity (severe brain injury, severe ROP, NEC, or late onset sepsis) at 36 weeks' PMA
Austria [7] (Urlesberger)	n/a	2018/2021	80 (not all preterm)	>=28 weeks' GA*, caesarean	DCC: <30 sec, cord milking after long clamping at 30cm, 1x 10cm/sec	Standard care (cord cutting, no milking)	Changes in cerebral blood volume within 15 min after birth
Bangladesh [8] (Yasmeen)	2014	2012/2013	40	<37 weeks' GA	DCC: 3 minutes	DCC: 1 minute	Haemoglobin, iron and ferritin
Canada [9] (Chu/Murphy)	n/a	2007/2010	296	Singletons, 24-32 weeks' GA	DCC: at 30-45 sec	ICC	Composite: IVH or late onset sepsis
Canada [10] (El-Naggar)	2018	2011/2014	73	24-30^{6} weeks' GA	UCM: x3, at or below the level of the placenta, ~20 cm milked, before clamping	ICC	Systemic blood flow (Superior vena cava flow at 4-6 hours after birth)
Canada [11] (Saigal)**	1972	n/a	125 (preterm)	Premature infants 28-36 weeks GA and weighing 1020g-3250g OR full-term infants 28-42 weeks GA and weighing 2685g-4350g*	Arm 1: DCC at 1min<br>Arm 2: DCC at 5min	ICC	RBC volume, blood volume, haematocrit, plasma volume
China [12] (Dai)	2014	n/a	52 (preterm)	Singletons, term and preterm infants*	DCC: Wait until cord pulsation ceased	ICC: 5-10 sec	RBC count (72-96 hrs after birth), Anaemia (2 wks), clinically significant pathological polycythaemia, white blood cell count (72-96 hrs after birth), fetal bilirubin from birth to day 5, jaundice within 24 hrs of birth, Apgar

iCOMP

Trial Country (PI)	Publication year	Start year/ completion year	Sample size	Participants	Intervention	Comparator	Primary outcome/s
							(1 min, 5 min), respiratory distress, rectal temperature 5 min after birth, neonate well-being at 1 month
China [13] (Dong)	2016	2015/2015	90	Singletons, <32 weeks' GA	DCC: 45 sec	ICC: <10 sec	Severe IVH – grades 3 and 4
China [14] (Hao)	n/a	2018/2019	48	30-31^{6} weeks' GA	UCM	DCC	Cerebral haemodynamics 15 min after birth
China [15] (Hu)	2015 (master's thesis)	n/a	120	28-35 weeks' GA, vaginal birth	Arm 1: DCC at 30 sec<br>Arm 2: DCC at 60 sec<br>Arm 3: DCC at 120 sec	ICC < 10 sec	Haematocrit and haemoglobin levels at 24 hrs and 1 wk after birth
China [16] (Hua)	2010?	2009/2011	176 (49 of those preterm)	Any GA*	Normal birth<br>Arm 1: DCC – wait until cord ceases pulsing<br>Arm 2: DCC – at 90 sec<br>Asphyxia<br>Arm 1: DCC – wait until cord ceases pulsing, resuscitate on bed site with cord intact	Normal birth<br>ICC <10 sec<br>Asphyxia<br>ICC <10 sec, resuscitate after on irradiation table	Haemoglobin 1 month after birth
China [17] (Li)	2018	2017/2017	102	delivered vaginally between 28^{0}-36^{6} weeks', and premature prolonged rupture of membranes	UCM: x4 at a speed of 10cm/sec, then clamped	ICC	Incidence of certain or probable infection in neonates
China [18] (Liu)	n/a	2019/2019	948 (not all preterm)	34^{0}-38^{6} weeks' GA*, caesarean section	DCC: 60 sec	ICC: within 10 sec	Rate of respiratory distress within 24 hours after birth
China [19] (Shi)	2017	n/a	60 preterm (and 460 term)	Single foetus deliveries*	DCC	ICC: 5-10 sec	Hemoglobin (newborn cord blood & after 24 hrs), neonatal complications, bleeding volume, third labour

For peer review only

iCOMP

Trial Country (PI)	Publication year	Start year/ completion year	Sample size	Participants	Intervention	Comparator	Primary outcome/s
							time, incidence of placental adhesion and peeling
China [20] (Xie)	n/a	2017/2019	300	Singletons, <34 weeks' GA	UCM: x2-3, 25cm/2 sec, below placenta level, before clamping	ICC	Haemoglobin, Haematocrit, and ferritin level at 48 hours
Egypt [21] (Allam)	n/a	2018/2019	210	Singletons, 30-34 weeks' GA	DCC: until cord stops pulsing or 1-2 min	ICC: <5 sec	Fetal haemoglobin, bilirubin, death
Egypt [22] (Nour 2017a)	n/a	2017/2019	90	<34 weeks' GA	UCM: x3 at 10cm/sec, below placenta level, cord held 20-25cm from baby	ICC	Peripheral venous CD34 at admission
Egypt [23] (Nour 2017b)	n/a	2017/2018	90	<34 weeks' GA	Arm 1: ICC, with placental insufficiency<br>Arm 2: DCC at 60sec, with placental insufficiency	Normal placenta with DCC at 60 sec	Peripheral venous CD34 at admission
Germany [24] (Nelle)	1998	n/a	19	PT <1500g*, born by caesarean section	DCC: 30 sec, 30 cm below placenta	ICC	Mean Blood Pressure, left ventricular output, mean cerebral blood flow velocity in the arteria carotis interna, haemoglobin, haematocrit , systemic and cerebral haemoglobin transport, systemic vascular resistance
Germany [25] (Rabe 2000)	2000	2006/2008	40	<33 weeks' GA	DCC: 45 sec	DCC: 20 sec	Feasibility, effects on post-partal adaption and anaemia of prematurity
India [26] (Aghai 2018)	n/a	2018/2020	1400 (not all preterm)	Depressed neonates, 35-42 weeks' GA*	UCM: x4, 30cm over 2 sec	ICC: immediately after birth	Number of infants with moderate to severe HIE or death
India [27] (Anusha)	n/a	2017/2019	148	birth weight <1500g*	DCC: 30 sec	ICC: within 10 sec	Haemodynamic stability, haematological status, serum ferritin, and requirement of

Trial Country (PI)	Publication year	Start year/ completion year	Sample size	Participants	Intervention	Comparator	Primary outcome/s
							blood transfusion between birth and 6 months of age
India [28] (Bhriguvanshi)	n/a	2017/2018	236	> 28 weeks' GA, requiring resuscitation*	UCM: x3 towards baby at 10cm/sec, then clamped	ICC: within 30 sec	Haemoglobin and haematocrit at birth and 6 weeks of age
India [29] (Chopra)	2018	2013/2015	142	growth retarded babies (IUGR) ≥ 35 weeks' GA*	DCC: 60 sec	ICC: 10 sec	Haemoglobin and ferritin levels
India [30] (Das/Sundaram)	2018	2012/2013	461	30-33^6 weeks' GA	DCC: 60 sec below placenta. If baby depressed, immediate clamping keeping cord long, milked x2-3 during resuscitation	ICC: within 10 sec	Composite of mortality or abnormal neurological examination at 40 weeks PMA
India [31] (Datta)	2017	2011/2013	120	Singletons, 34-36^6 weeks' GA	DCC: at >30-<60 sec	ICC: <20 sec	Neurobehavioural Assessment of Preterm Infant at 37 weeks' post-conceptional age
India [32] (Dhaliwal)	2014	n/a	300	34-37 weeks' GA	DCC: 60 sec	ICC: <10 sec	Risk of neonatal mortality & abnormal neurological examination at 40 weeks' GA
India [33] (Dipak)	2017	2012/2013	78	27-31^6 weeks' GA	Arm 1: DCC: 60 sec<br>Arm 2: DCC: 60 sec with intramuscular ergometrine	ICC: <10 sec	Hematocrit 4 h after birth
India [34] (George/Isac)	n/a	2017/2018	180 (not all preterm)	Mothers at 34-40^6 weeks' GA*	UCM: milking whole length at 10cm/sec x3, then clamped	ICC	Infant haemoglobin and haematocrit at 72hrs and 6 weeks
India [35] (Gupta)	n/a	2018/2020	110	<34 weeks' GA	DCC: 30 sec	ICC	Ferritin and PCV at 8 weeks
India [36] (Kumar)	2015	2013/2014	200	32-36^6 weeks' GA, vaginal or caesarean	UCM: x3, 10cm/sec	ICC	Haemoglobin and ferritin at 1.5 months

For peer review only - http://bmjopen.bmj.com/site/about/guidelines.xhtml

iCOMP

Trial Country (PI)	Publication year	Start year/ completion year	Sample size	Participants	Intervention	Comparator	Primary outcome/s
India [37] (Ram Mohan)	2018	2015/2016	60	<37 weeks' GA, requiring resuscitation	UCM: 20-25 cm umbilical cord x3 at 10cm/sec within 30 sec of birth	No milking	Haemoglobin and serum ferritin at 6 weeks
India [38] (Rana/Agarwal)	2018	2013/2013	100	<34 weeks' GA	DCC: 120 sec	ICC: ≤30 sec	serum total bilirubin and haematocrit levels at 48 hrs and 7 days
India [39] (Ranjit)	2015	2010/2010	100	30-36^6 weeks' GA	DCC: >2min	ICC	Haematocrit and serum ferritin at 6 weeks
India [40] (Kumar Mangla/ Thukral)	n/a	2016/2017	144 (not all preterm)	Late preterm and term neonates*	Deferred UCM: cord clamped at 60 sec	UCM: Cord milking in 10 sec	Venous haematocrit at 48 hours of life
India [41] (Upadhyay 2010)	2013	2010/2011	170 (not all preterm)	>35 weeks' GA*	UCM: x3 at 10cm/sec, then clamped at ~25 cm of length within 30 sec of birth	ICC: <30 sec	Haemoglobin and serum ferritin at 6 weeks
India [42] (Varanattu)	n/a	2018/2019	250	<32 weeks' GA	UCM: x3 over 20 sec at 20cm/2sec with 2 second pause between	ICC: clamped immediately	Haemoglobin levels at birth and IVH (incidence and severity) at 7 days
Iran [43] (Armanian)	2017	2014/2015	63	≤34 weeks' GA	DCC: at 30-45 sec	ICC: at 5-10 sec	Time of cord clamping
Iran [44] (Mojaveri)	2017	2014/2015	70	<32 weeks' GA, caesarean, birth weight < 1500g, not requiring advanced resuscitation	DCC: at 30-45 sec	ICC: <10 sec	IVH (days 3 to 7), survival infant (up to 28 days)
Iran [45] (Mirzaeian)	n/a	2017/2018	160	28-34 weeks' GA	UCM: milked x3 in 10 sec	ICC	Amount of transfused blood, bilirubin levels
Iran [46] (Sekhavat)	2008	n/a	52	26-34 weeks' GA	DCC: 30-60 sec	ICC: 10-15 sec	Blood pressure, haematocrit, blood glucose

iCOMP

Trial Country (PI)	Publication year	Start year/ completion year	Sample size	Participants	Intervention	Comparator	Primary outcome/s
Iran [47] (Shahgheibi)	n/a	2017/2018	90	Women with preterm labour	DCC: 180 sec	DCC: 30 sec	blood parameters, weaning from ventilator, NICU discharge time
Ireland [48] (Dempsey)	n/a	2015/2016	45	<32 weeks' GA	Arm 1: DCC at 60 sec on mobile resuscitation trolley at/below placenta level<br>Arm 2: UCM – Cord stripped 3 times at 20cm/2 sec at/below placenta level	ICC: <20 sec	Neonatal: Brain activity (6 & 12 hours post-partum, EEG and NIRS)<br>Maternal: hemoglobin at 24-36 hours post-partum
Israel [49] (Kugelman)	2007	2004/2005	65	$24\text{-}35^6$ weeks' GA	DCC: 30-45 sec, below placenta level	ICC: <10 sec	Haematocrit, blood pressure
Italy [50] (Pratesi)	2018	2016/2017	40	$23\text{-}29^6$ weeks' GA	Bedside assistance with intact placental circulation	UCM: x4, 10cm/sec, before clamping	Feasibility (recruitment rate, compliance, completeness, receiving echographic assessment) and safety
Japan [51] (Hosono 2008)	2008	2001/2002	40	24-28 weeks' GA, singletons	UCM: 20 cm of the cord, x2-3, before clamping, 20cm/2sec	ICC	Probability of not needing transfusion, number of RBC transfusions
Japan [52] (Hosono 2016)	2016	2008/2016	203	$24\text{-}27^6$ weeks' GA	UCM: cord cut 30 cm from infant, cord milked x1	ICC: <30 sec	1) Probability needing transfusion and death<br>2) Amount of blood transfusion first 4 weeks
Korea [53] (Song)	2017	2012/2015	66	$24\text{-}32^6$ weeks' GA	UCM: x4 at 20cm/2sec, with 2 sec pause between	ICC: immediately after delivery	Short term safety: Apgar score, prevalence of hypothermia, early intubation, initial blood gas analyses, bilirubin levels, duration of phototherapy, use of cross-transfusion, respiratory distress.

iCOMP

Trial Country (PI)	Publication year	Start year/ completion year	Sample size	Participants	Intervention	Comparator	Primary outcome/s
Macedonia [54] (Zisovska)	2008	n/a	57	Premature newborns	DCC: 1 min	ICC	Hematological parameters, number of RBC transfusions
Nepal [55] (Andersson)	2017	2014/2017	540 (not all preterm)	34-41 weeks' GA*	DCC: at ≤180 sec	ICC: ≤30 sec	Haemoglobin at 8±1 months
Nepal [56] (Ashish KC)	n/a	2016/2016	1510 (not all preterm)	Singletons, normal vaginal delivery, ≥33 weeks' GA*	DCC: at 180 sec	DCC: <60 sec	Neonatal heart rate continuously until 10 min after birth and at 1,3&5 min
Netherlands [57] (Knol)	n/a	2018/2019	64	<32 weeks' GA	Physiology-based cord clamping:stabilisation while the cord is intact, cord clamped when infant is respiratory stable (regular spontaneous breathing, heart rate >100 bpm, oxygen>90%, supplemental oxygen <40%)	ICC/DCC: immediately or delayed 30-60 sec, depending on clinical condition of infant	Time needed to stabilise the infant
Netherlands [58] (Te Pas)	n/a	2019/2020	660	<30 weeks' GA	Physiology-based cord clamping: Resuscitation with cord intact, clamp when infant is stable (heart rate >100 bpm, oxygen>85%, supplemental oxygen <40%)	ICC/DCC: immediately or delayed 30-60 sec, depending on clinical condition of infant	Intact survival at NICU discharge without cerebral injury (IVH ≥ grade 2 and/or PVL ≥ grade 2 and/or periventricular venous infarction) and/or NEC (Bell stage ≥ 2)
Netherlands [59] (Ultee)	2008	n/a	37	34 to 36^{6} weeks' GA	DCC: 3 min	ICC: <30 sec	blood glucose levels at 1,2, and 3hrs of age, haemoglobin and haematocrit at 1hr and 10 weeks, ferritin at 10 weeks
New Zealand [60] (Meyer/Nevill)	n/a	2016/2020	120	<31 weeks' GA, not breathing regularly at 15sec	Positive pressure ventilation and continuous positive	DCC: 50sec with thermal wrap	RBC transfusion rates

iCOMP

Trial Country (PI)	Publication year	Start year/ completion year	Sample size	Participants	Intervention	Comparator	Primary outcome/s
					airway pressure until cord clamping at 50s		
Pakistan [61] (Malik)	2013	2009-2009	80	30-37 weeks' GA	DCC: 120 sec	DCC: 30 sec	Haematocrit
Saudi Arabia [62] (Al-Wassia)	n/a	2017/2019	180	<32 weeks' GA	UCM: milked 20cm segment over 2-3 sec x3	DCC: 60 sec	IVH at 28 days
Saudi Arabia [63] (Gomaa)	n/a	2016/2018	200	24 to 34^6 weeks' GA	DCC: 45-60 sec, baby at level or below placenta	UCM: milked x4-5 from maternal end of cord to baby abdomen, 2 sec pause between milking	Haematological parameters - haematocrit
South Africa [64] (Hofmeyr 1988)	1988	n/a	38	Singleton, <35 weeks' GA	Arm 1: DCC at 60sec<br>Arm 2: DCC at 60sec + ergometrine	ICC	PVH/IVH at 6-72hrs after birth, Apgar score at 5min, birthweight, systolic blood pressure at 5min, cord blood gas and death.
South Africa [65] (Hofmeyr 1993)	1993	n/a	86	<2000 g birthweight*	DCC: 1-2 min	ICC	death of the baby, PVH/IVH at 6-72hrs after birth, Apgar score at 5min, cord-pH, bilirubin
South Africa [66] (Tiemersma)	2015	2012/2012	104 (not all preterm)	Birth weight <2500g ± 500g*	DCC: 2-3 minutes	ICC: within 30 sec	Haemoglobin from cord blood and at 2 months
Spain [67] (De Paco Matallana)	n/a	2011/2014	100	24- 34 weeks' GA	DCC: 45-60 sec	ICC: <10 sec	Neonatal haemoglobin, haematocrit and bilirubin levels (within 7 days after birth)
Spain [68] (Domingo Puiggrós)	n/a	2014/2016	40	<34 weeks' GA, caesarean	UCM: x3 at 20 cm/2sec	DCC: 30 sec	Haemoglobin at 1 and 24 hrs

For peer review only - http://bmjopen.bmj.com/site/about/guidelines.xhtml

iCOMP

Trial Country (PI)	Publication year	Start year/ completion year	Sample size	Participants	Intervention	Comparator	Primary outcome/s
Spain [69] (Leal)	2019	2013/2016	138	24^{0}-36^{6} weeks' GA	UCM: nearly 20cm cord milked towards umbilicus x4 before clamping	ICC: <20 sec	Requirement of RBC transfusions or phototherapy
Spain [70] (Socias)	n/a	2014/2017	150	26-32^{6} weeks' GA	DCC: 30-60 sec	ICC: <30sec	RBC transfusions (number & volume), IVH, postpartum haemorrhage
Switzerland [71] (Baenziger)	2007	1996/1997	39	24-32 weeks' GA	DCC: 60-90 sec, below placenta, syntocinon	ICC: <20 sec	Cerebral oxygenation at 4, 24 and 72 hrs of age
Taiwan [72] (Shen)	n/a	2015/2019	100	<30 weeks' GA	UCM: milked once after ICC, 20cm section at speed of 10cm/sec and clamped at 2-3cm.	ICC and no milking	Neonate's haemoglobin, haematocrit, and mean arterial pressure at admission
Thailand [73] (Chamnanvanakij)	2017	2015/2016	46	25-34 weeks' GA	UCM: x3-4, 30 cm, before clamping	DCC: at 60 sec	Haematocrit level 2 hrs after birth
Thailand [74] (Jomjak)	n/a	2018/2018	110	Singleton, 24-36^{6} weeks' GA	DCC	ICC	Haematocrit at 2 and 48 hrs
Thailand [75] (Mungkornkaew)	2015	2014/2014	200 (not all preterm)	Singleton, 34-42 weeks' GA*, vaginal delivery	DCC: 2 minutes	DCC: 1 minute	Fetal haematocrit
Thailand [76] (Panichkul)	n/a	2015/2016	70	34-36 weeks' GA	DCC: at 60 sec	ICC: at 10 sec	Haematocrit 2 hours after birth
Thailand [77] (Prachukthum)	n/a	2016/2018	120	28-33^{6} weeks' GA	Arm 1: UCM x3 before cord clamping at 45 sec<br>Arm 2: DCC at 45 sec, followed by UCM x3	DCC: 45 sec	RBC transfusion received
Thailand [78] (Ruangkit)	2019	2016/2017	47	Multiples, 28-36 weeks' GA	DCC: at 30-60 sec	ICC: <10 sec	Haematocrit level at birth
Thailand [79] (Salae)	2016	2014/2015	86	34-36^{6} weeks' GA	DCC: at 2 minutes	ICC: within 30 sec	Haematocrit at 48 hours

For peer review only - http://bmjopen.bmj.com/site/about/guidelines.xhtml

Trial Country (PI)	Publication year	Start year/ completion year	Sample size	Participants	Intervention	Comparator	Primary outcome/s
Thailand [80] (Tanthawat)	n/a	2016/2016	40	<32 weeks' GA	UCM: Cut cord at 30cm, cord milking x1 at 10cm/sec, clamp and cut cord at 1-2cm from umbilical stump	ICC: <10 sec	Haemoglobin and haematocrit level at admission
Turkey [81] (Alan)	2014	2011/2013	44	≤32 weeks' GA ≤1500 g	UCM: at 25-30 cm x3 at 5cm/s before clamping	ICC: <10 sec	Number and volume of packed RBC transfusions received by infant during first 35 days of life
Turkey [82] (Gokmen)	2011	2008/2009	42	24-31^6 weeks' GA	DCC: 30-45 sec	ICC: 5-10 sec	peripheral blood hematopoietic progenitor cells before any blood product administered to infants
Turkey [83] (Kilicdag)	2015	2012/2013	54	≤32 weeks' GA	UCM: x4 before clamping (20cm/2sec)	ICC	absolute neutrophil counts
Turkey [84] (Silahli)	2018	2015/2016	75	≤32 weeks' GA	UCM: at 20 cm x3, before clamping	ICC: <10 sec	Thymic size
UK [85] (Aladangady)	2006	n/a	46	24-32^6 weeks' GA	DCC: 30-90 sec, below placenta, oxytocic agent, with ventilation/ resuscitation if necessary	ICC	Infants' blood volume
UK [86] (Duley)	2018	2013/2015	261	<32 weeks' GA	DCC: after at least 2 min	ICC: <20 sec	Death before hospital discharge, intraventricular haemorrhage
UK (Holland)	Not published	1998/2001	?	<33 weeks' gestation	DCC: 40-90 s	ICC (?)	Median arterial/alveolar PO2 ratio over the first 24 hrs of life
UK [87] (Kinmond)	1993	n/a	36	>27 & <32 weeks' GA, vaginal delivery	DCC: 30 sec, 20 cm below placenta	ICC: 10 sec median	Initial packed cell volume, peak serum bilirubin concentrations, red

For peer review only

iCOMP

Trial Country (PI)	Publication year	Start year/ completion year	Sample size	Participants	Intervention	Comparator	Primary outcome/s
							cell transfusions, respiratory impairment
England [88] (Medina)	2013	n/a	51	24-31 weeks' GA	DCC	ICC	Haemodynamic parameters, included vena cava blood flow, ventricular outflow, and flow velocity.
UK [89] (Rabe)	2011	2006/2008	58	Singleton, <34 weeks' GA	UCM: x4	DCC: at 30 sec	neonatal blood haematocrit and haemoglobin at 1 hr after birth
USA [90] (Backes)	2016	2009/2013	40	Singletons, 22^5-27^6 weeks' GA	DCC: 30-45 sec, below placenta	ICC: <10 sec	Safety, feasibility, haematological and circulatory outcomes
USA [91] (Bauer)	n/a	2014/2019	300	>24 and <30 weeks' GA	Arm 1: DCC at 45 sec and indomethacin within 6 hrs<br>Arm 2: DCC at 45 sec and placebo within 6 hrs	Arm 3: ICC and indomethacin<br>Arm 4: ICC and placebo	Fraction of survivors with no severe IVH (grades 3 or 4) or PVL within first 60 days of life
USA [92] (Berens)	n/a	2018/2019	100 (not all preterm)	≥35 weeks GA*, at least 1 previous child that received phototherapy for hyperbilirubinemia	DCC: 60 sec	ICC: <15 sec	Neonatal bilirubin level 24 hours after birth
USA [93] (Bienstock)	n/a	2011/2013	22	24^0-32^6 weeks' GA	UCM: x4 over 10 min	ICC	Haemoglobin within 24 hours of birth and through NICU stay
USA [94] (March/deVeciana)	2013	2009/2011	113	Singletons, 24-28^6 weeks' GA	UCM: 10cm, immediately after delivery, ~20cm actively milked x 3	ICC	RBC transfusion within 28 days of life
USA [95] (Driggers)	n/a	2011/2013	2	Infants delivered at 24^0 to 28^6 weeks' GA	Arm 1: DCC at 30 sec<br>Arm 2: UCM x4 in 10 sec	Arm 3: ICC	Adverse neonatal event: composite of BPD, NEC,

For peer review only - http://bmjopen.bmj.com/site/about/guidelines.xhtml

Trial Country (PI)	Publication year	Start year/ completion year	Sample size	Participants	Intervention	Comparator	Primary outcome/s
							grade 3 or 4 IVH or PVL, or death prior to discharge
USA [96] (Elimian)	2014	2008/2011	200	Singletons, 24-34 weeks' GA	DCC: at 30-35 sec (3-4 passes of milking toward the neonate was allowed)	ICC: <5 sec	Need for blood transfusion
USA [97] (Ibrahim)	2000	n/a	32	Birthweight 501g-1250g, 24 to <29 weeks' GA	DCC: 20 sec	ICC	Number of blood transfusions
USA [98] (Josephsen)	n/a	2012/2016	80	24-27^{6} weeks' GA	UCM: below level of placenta and ~20 cm cord milked x3 over 10-20 sec before clamping	ICC	Haemoglobin and haematocrit concentrations (within 4 hrs birth) Incidence and number blood transfusions until discharge
USA [99] (Katheria 2011)	2014 & 2017	2011/2013	60	<32 weeks' GA	UCM: x3, below placenta, about 20cm of cord over 2 sec	ICC	Superior vena cava flow at 6 hours
USA [100] (Katheria 2013)	2015 & 2017	2013/2018	197	23-31^{6} weeks' GA	UCM: x4 at 20 cm/2 sec	DCC: at 45-60 sec	Superior vena cava flow at <12 hrs
USA [101] (Katheria 2016)	2016	2014/2015	150	<32 weeks' GA	CPAP + DCC at 60s	DCC: 60s	Peak haematocrit in first 24 hours of life
USA [102] (Katheria 2017)	n/a	2017/2022	1200	23-32^{6} weeks' GA	UCM: x4 at 20cm/2 sec	DCC: at least 60 sec	Incidence of IVH or death at discharge, up to 6 months corrected gestational age
USA [103] (Katheria 2019)	n/a	2019/2020	1000 (not all preterm)	Non-vigorous newborns born at 35-42 weeks' GA*	UCM: x4, entire umbilical length over 2 sec.	ICC: within 30 sec	Admission to NICU in the first 48 hrs of life
USA [104] (Kattwinkel)	n/a	2016/2021	940	23^{0}-28^{6} weeks' GA	DCC: Assisted ventilation (face mask, CPAP or PPV) prior to DCC at 120 sec	DCC: 30-60 sec, assisted ventilation only after cord clamping	IVH (7-10 days)

iCOMP

Trial Country (PI)	Publication year	Start year/ completion year	Sample size	Participants	Intervention	Comparator	Primary outcome/s
USA [105] (Krueger)	2015	2012/2013	67	Singletons, 22-31^{6} weeks' GA	DCC & UCM: cord milking x4 with 4-5 sec between each, then DCC at 30 sec	DCC: 30 sec, without cord milking	Initial fetal haematocrit
USA [106] (Martin)	n/a	2012/2014	72	Singletons, 23-37 weeks' GA	Arm 1: DCC at 60 sec<br>Arm 2: DCC at 40 sec	DCC: 20 sec	IVH number and severity (15 months)
USA [107] (Mercer 2003)	2003	1998/2001	32	Singletons, 24-31^{6} weeks' GA	DCC: 30-45 sec	ICC: 5-10 sec	Initial mean blood pressure on arrival in NICU
USA [108] (Mercer 2006)	2006	2003/2004	72	Singletons, <32 weeks' GA	DCC: 30-45 sec	ICC: 5-10 sec	BPD, suspected NEC
USA [109] (Mercer 2008)	2011 & 2016 & 2018	2008/2014	211	Singletons, 24-31^{6} weeks' GA	DCC & UCM: milking x1 then DCC at 30-45 sec. If clamping cannot be deferred, cord milked x2-3 quickly	ICC: <10 sec	IVH, late onset sepsis
USA [110] (Oh)	2011	2000/2001	33	Singletons, 24-27^{6} weeks' GA	DCC: at 30-45 sec	ICC: <10 sec	venous haematocrit at 4 hours of age
USA [111] (Perlman)	n/a	2015/2019	150	28-34^{6} weeks' GA	DCC: at 60 sec	DCC: at 30 sec	Haematocrit 1 hour after birth
USA [112] (Smith)	n/a	2014/2018	282	23^{0}-34^{6} weeks' GA	UCM: x4, before clamping	DCC: at 30 sec	Haemoglobin & haematocrit in NICU from admission to discharge
USA [113] (Strauss)	2008	n/a	158[101]	≤36 weeks' GA	DCC: 60 sec	ICC	RBC volume/mass, per biotin labelling
USA [114] (Yared/Young)	n/a	2015/2016	39	Very low birth weight (500 to 1500 grams)*	DCC: at 60 sec	DCC: at 30 sec	IVH (during NICU admission up to 6 months)
Thailand [115] (Pongmee)	2010 (abstract)		43	<35 weeks' GA	UCM: x2 along 30 cm after cutting	ICC	Initial haematocrit, need for blood transfusion

* only those born <37 weeks gestation eligible

** PI advised individual participant data not available due to time elapsed since trial

PI = Principal Investigator cm = centimetres sec = seconds min = minutes

For peer review only - http://bmjopen.bmj.com/site/about/guidelines.xhtml

iCOMP

NICU = neonatal intensive care unit
DCC = deferred cord clamping
CPAP = continuous positive airway pressure
PVL = periventricular leukomalacia
IVH = intraventricular haemorrhage
NEC = necrotising enterocolitis
GA = gestational age
UCM = umbilical cord milking
PO2 = partial pressure of oxygen
ROP = retinopathy of prematurity
PVH = periventricular haemorrhage
IUGR = intrauterine growth retardation
PMA = postmenstrual age
PBCC = physiological based cord clamping
PPV = positive pressure ventilation
BPD = bronchopulmonary dysplasia
HIE = hypoxic ischemic encephalopathy
PCV = polycythaemia
ICC = immediate cord clamping
RBC = red blood cell
NIRS = near-infrared spectroscopy
EEG = electroencephalogram

1. ISRCTN registry website: **Early compared to delayed umbilical cord clamping in very small prematurely born babies: A study to know which one is better for infant health. http://www.isrctn.com/ISRCTN12219110. Accessed August 27, 2018.**
2. ANZCTR registry website: **Resuscitating newborn infants with the umbilical cord intact- The Baby-Directed Umbilical Cord Cutting (Baby-DUCC) Trial. http://www.anzctr.org.au/ACTRN12618000621213.aspx. Accessed 20th February, 2019.**
3. McDonnell M, Henderson-Smart D: **Delayed umbilical cord clamping in preterm infants: a feasibility study**. *Journal of paediatrics and child health* 1997, **33**(4):308-310.
4. ANZCTR website: **CORD CLAMPING STUDY: Early versus delayed cord clamping and its effects on infant heart rate and oxygen saturation in the first minutes after birth. https://www.anzctr.org.au/Trial/Registration/TrialReview.aspx?id=365659. Access 20th February, 2019.**
5. Australian New Zealand Clinical Trials Registry: **Australian Placental Transfusion Pilot Study: investigating standard cord clamping procedures versus three methods of autologous placental blood transfusion in pre term infants. https://www.anzctr.org.au/Trial/Registration/TrialReview.aspx?ACTRN=12609000248268. Accessed February 20, 2019.**
6. Tarnow-Mordi W, Morris J, Kirby A, Robledo K, Askie L, Brown R, Evans N, Finlayson S, Fogarty M, Gebski V *et al*: **Delayed versus Immediate Cord Clamping in Preterm Infants**. *New England Journal of Medicine* 2017, **377**(25):2445-2455.
7. ClinicalTrials.gov registry: **C-UCM and Cerebral Oxygenation and Perfusion. Available at https://clinicaltrials.gov/ct2/show/NCT03748914. Accessed February 20, 2019.**
8. Yasmeen S, Shahidullah M, Mannan M, Dey A, Chowdhury R, Haque A, Hassan MZ: **Iron Status in Early versus Delayed Cord Clamping Groups of Preterm Neonates Delivered in a Tertiary Level Hospital**. *Journal of Armed Forces Medical College, Bangladesh* 2015, **10**(2):62-65.
9. Chu K, Whittle W, Windrim R, Shah P, Murphy K: **The DUC trial: A pilot randomized controlled trial of immediate vs. delayed umbilical cord clamping in preterm infants born between 24 and 32 weeks gestation**. *American Journal of Obstetrics and Gynecology* 2011, **204**(1 Suppl):S201.
10. El-Naggar W, Simpson D, Hussain A, Armson A, Dodds L, Warren A, Whyte R, McMillan D: **Cord milking versus immediate clamping in preterm infants: a randomised controlled trial**. *Archives of Disease in Childhood - Fetal and Neonatal Edition* 2019, **104**(2):F145-F150.
11. Saigal S, O'Neill A, Surainder Y, Chua LB, Usher R: **Placental transfusion and hyperbilirubinemia in the premature**. *Pediatrics* 1972, **49**(3):406-419.
12. Dai S, Jin X, Qi HJ: **Effect of different cord clamping times on the prognosis of newborns (不同断脐时间对新生儿预后的影响)**. *Modern Pract Med* 2014, **26**:69-71.
13. Dong XY, Sun XF, Li MM, Yu ZB, Han SP: **Influence of delayed cord clamping on preterm infants with a gestational age of <32 weeks [in Chinese]**. *Zhongguo Dang Dai Er Ke Za Zhi* 2016, **18**:635-638.

For peer review only - http://bmjopen.bmj.com/site/about/guidelines.xhtml

14. Chinese Clinical Trial Registry website: **Effect of delayed cord clamping versus unbilical cod milking on cebral blood flow in preterm infant: a randomized, double-blind controlled trial. http://www.chictr.org.cn/showprojen.aspx?proj=30981. Accessed February 20, 2019.**
15. Hu X, Xu X: **The Effects of Different Cord Clamping Time in Preterm Infants by Vaginal Delivery [Master's Degree Thesis]** Zhejiang University; 2015.
16. Clinical Trials website: **Immediate VS Delayed Cord Clamping on Newborns**. *https://clinicaltrialsgov/ct2/show/NCT01029496 last accessed August 30, 2018*.
17. Li J, Yu B, Wang W, Luo D, Dai Q-l, Gan X-q: **Does intact umbilical cord milking increase infection rates in preterm infants with premature prolonged rupture of membranes?** *The Journal of Maternal-Fetal & Neonatal Medicine* 2018:1-7.
18. Chinese Clinical Trial Registry website: **Delayed cord clamping prevents respiratory distress of infants delivered by selective cesarean section, a randomized controlled trial. http://www.chictr.org.cn/showprojen.aspx?proj=30199. Accessed February 20, 2019**.
19. Shi W, Peng J, Chen N: **Effect of delayed cord clamping on outcome of delivery**. *Henan Journal of Preventive Medicine* 2017, **28**:85-87.
20. Clinical Trials website: **The Study on Umbilical Cord Milking to Prevent and Decrease the Severity of Anemia in Preterms**. *https://clinicaltrialsgov/ct2/show/NCT03023917 last accessed August 31, 2018*.
21. Australian New Zealand Clinical Trials Registry: **Delayed fetal cord clamping in preterm labour, Risk and benefits. https://www.anzctr.org.au/Trial/Registration/TrialReview.aspx?id=374731. Accessed February 21, 2019.**
22. ClinicalTrials.gov registry: **Impact of Umbilical Cord Milking in Preterm Neonates With Placental Insufficiency**. *https://clinicaltrialsgov/ct2/show/NCT03731611 Accessed February 21, 2019*.
23. ClinicalTrials.gov registry: **Effect of Delayed Cord Clamping in Preterm Neonates With Placental Insufficiency, available at https://clinicaltrials.gov/ct2/show/NCT03731546. Last accessed Feb 21, 2019.**
24. Nelle M, Fisher S, Conze S, Beedgen B, Linderkamp O: **Effects of late cord clamping on circulation in prematures (VLBWI)**. *Pediatric Research* 1998, **44**(3):454.
25. Rabe H, Wacker A, Hülskamp G, Hörnig-Franz I, Schulze-Everding A, Harms E, Cirkel U, Louwen F, Witteler R, Schneider HP: **A randomised controlled trial of delayed cord clamping in very low birth weight preterm infants**. *Eur J Pediatr* 2000, **159**(10):775-777.
26. ClinicalTrials.gov registry: **Umbilical Cord Milking in Neonates Who Are Depressed at Birth (MIDAB). Available at https://clinicaltrials.gov/ct2/show/NCT03657394. Last accessed February 21, 2019.**
27. Clinical Trials Registry India website: **Comparison of advantages and disadvantages of early and delayed umbilical cord clamping in low birth weight newborns. http://ctri.nic.in/Clinicaltrials/pdf_generate.php?trialid=16653&EncHid=&modid=&compid=%27,%2716653det%27 last accessed February 21, 2019.**
28. Clinical Trials Registry India website: **The effects of umbilical cord squeezing in newborn babies requiring some form of intervention to help and support breathing at birth. http://www.ctri.nic.in/Clinicaltrials/pdf_generate.php?trialid=18641&EncHid=&modid=&compid=%27,%2718641det%27, last accessed February 21, 2019.**
29. Chopra A, Thakur A, Garg P, Kler N, Gujral K: **Early versus delayed cord clamping in small for gestational age infants and iron stores at 3 months of age-a randomized controlled trial**. *BMC pediatrics* 2018, **18**(1):234.

For peer review only - http://bmjopen.bmj.com/site/about/guidelines.xhtml

30. Das B, Sundaram V, Tarnow-Mordi W, Ghadge A, Dhaliwal LK, Kumar P: **Placental transfusion in preterm neonates of 30–33 weeks' gestation: a randomized controlled trial**. *Journal of Perinatology* 2018, **38**:496–504.
31. Datta V, Kumar A, Yadav R: **A randomized controlled trial to evaluate the role of brief delay in cord clamping in preterm neonates (34-36 weeks) on short-term neurobehavioural outcome**. *Journal of Tropical Pediatrics* 2017, **63**(6):418–424.
32. Dhaliwal LK, Anjumunnisa S, Kumar P, Saha PK, Venkataseshan S: **Effects of placental transfusion in preterm birth**. *J Matern Fetal Neonatal Med* 2014, **27**(S1):369-370.
33. Dipak NK, Nanavat RN, Kabra NK, Srinivasan A, Ananthan A: **Effect of Delayed Cord Clamping on Hematocrit, and Thermal and Hemodynamic Stability in Preterm Neonates: A Randomized Controlled Trial.** *Indian Pediatr* 2017, **54**(2):112-115.
34. Clinical Trials Registry India website: **Effect of umbilical cord milking of late preterm and term infants on maternal and neonatal outcomes in a tertiary care hospital in South India: A randomized control trial. http://www.ctri.nic.in/Clinicaltrials/pmaindet2.php?trialid=19631 last accessed February 21, 2019**.
35. Clinical Trials Registry India website: **Early versus Delayed Cord clamping in IUGR Preterms a Randomised controlled study. http://ctri.nic.in/Clinicaltrials/showallp.php?mid1=25064&EncHid=&userName=015204 last accessed February 21, 2019.**
36. Kumar B, Upadhyay A, Gothwal S, Jaiswal V, Joshi P, Dubey K: **Umbilical Cord Milking and Hematological Parameters in Moderate to Late Preterm Neonates: A Randomized Controlled Trial**. *Indian Pediatr* 2015, **52**(9):753-757.
37. Clinical Trials Registry India website: **Umbilical cord milking in preterm neonates requiring resuscitation: A randomized controlled trial** *http://wwwctrinicin/Clinicaltrials/pmaindet2php?trialid=10609 last accessed August 31, 2018*.
38. Rana A, Agarwal K, Ramji S, Gandhi G, Sahu L: **Safety of delayed umbilical cord clamping in preterm neonates of less than 34 weeks of gestation: a randomized controlled trial**. *Obstetrics & Gynecology Science* 2018, **61**(6):655-661.
39. Ranjit T, Nesargi S, Rao PNS, Sahoo JP, Ashok C, Chandrakala BS, Bhat S: **Effect of Early versus Delayed Cord Clamping on Hematological Status of Preterm Infants at 6 wk of Age**. *The Indian Journal of Pediatrics* 2015, **82**(1):29-34.
40. Clinical Trials Registry India website: **Comparison of Umbilical cord milking with Delayed cord clamping in late preterm and term neonates: Randomized control trial. www.ctri.nic.in/Clinicaltrials/pmaindet2.php?trialid=14204. Accessed August 31, 2018**.
41. CLinical Trials Registry India website: **Effect of cord milking on hemodynemic status and morbidities in neonates more than 35 weeks of gestation.** *http://wwwctrinicin/Clinicaltrials/pmaindet2php?trialid=3182 last accessed August 31, 2018*.
42. ClinicalTrials.gov registry: **Effect of Intact Umbilical Cord Milking on Neonatal and First Year Neurodevelopmental Outcomes in Very Preterm Infants. (Cord Milking)**. *https://clinicaltrialsgov/ct2/show/NCT03200301 last accessed February 22, 2019*.
43. Armanian AM, Ghasemi Tehrani H, Ansari M, Ghaemi S: **Is "Delayed Umbilical Cord Clamping" Beneficial for Premature Newborns?** *Int J Pediatr* 2017, **5**(5):4909-4918.
44. Kazemi MV, Akbarianrad Z, Zahedpasha Y, Mehraein R, Mojaveri MH: **Effects of Delayed Cord Clamping on Intraventricular Hemorrhage in Preterm Infants**. *Iran J Pediatr* 2017 **27**(5):e6570.
45. Iranian Registry of Clinical Trials website: **Investigation and comparison of neonatal complications of two methods of umbilical cord milking and early cord clamping in neonates**. *http://enirctir/trial/29424 last accessed February 22, 2019*.

iCOMP

46. Sekhavat L, Tabatabaii A: **Immediate and delayed cord clamping in infants born between 26 and 34 weeks**. *Journal of Maternal-Fetal and Neonatal Medicine* 2008, **21**((Suppl 1)):181–182.
47. Iranian Registry of Clinical Trials website: **The delayed umbilical cord clamping effects on early outcome in Preterm neonates**. http://enirctir/trial/17924 *last accessed February 22, 2019*.
48. ISRCTN registry website: **Clamping the umbilical cord in premature deliveries (CUPID)**. http://wwwisrctncom/ISRCTN92719670 *last accessed August 27, 2018*.
49. Kugelman A, Borenstein-Levin L, Riskin A, Chistyakov I, Ohel G, Gonen R, Bader D: **Immediate versus delayed umbilical cord clamping in premature neonates born < 35 weeks: a prospective, randomized, controlled study**. *Am J Perinatol* 2007, **24**(5):307-315.
50. Pratesi S, Montano S, Ghirardello S, Mosca F, Boni L, Tofani L, Dani C: **Placental circulation intact trial (PCI-T)-resuscitation with the placental circulation intact versus cord milking for very preterm infants: a feasibility study**. *Frontiers in pediatrics* 2018, **6**:364.
51. Hosono S, Mugishima H, Fujita H, Hosono A, Minato M, Okada T, Takahashi S, Harada K: **Umbilical cord milking reduces the need for red cell transfusions and improves neonatal adaptation in infants born at less than 29 weeks' gestation: a randomised controlled trial**. *Arch Dis Child Fetal Neonatal Ed* 2008, **93**(1):F14-19.
52. UMIN clinical trials registry: **A multicenter randomized control study of the effect of umbilical cord milking**. https://uploaduminacjp/cgi-open-bin/ctr_e/ctr_viewcgi?recptno=R000001193 *last accessed 31 August, 2018*.
53. Song SY, Kim Y, Kang BH, Yoo HJ, Lee M: **Safety of umbilical cord milking in very preterm neonates: a randomized controlled study**. *Obstet Gynecol Sci* 2017, **60**(6):527-534.
54. Zisovska E, Lazarevska L, Spasova L, Zivkovik J: **The effect of delayed cord clamping on the need for blood transfusion: P17**. *Transfusion Alternatives in Transfusion Medicine* 2008, **10**.
55. Clinical Trials website: **Effect of Timing of Umbilical Cord Clamping on Anaemia at 8 and 12 Months and Later Neurodevelopment.** https://clinicaltrialsgov/ct2/show/NCT02222805 *last accessed August 27, 2018*.
56. ISRCTN registry website: **Effect of timing of cord clamping in newborns during first 10 minutes of birth**. http://wwwisrctncom/ISRCTN10944304 *last accessed August 27, 2018*.
57. Knol R, Brouwer E, Klumper F, Van den Akker T, DeKoninck P, Hutten J, Lopriore E, Van Kaam A, Polglase GR, Reiss I: **Effectiveness of Stabilization of Preterm Infants With Intact Umbilical Cord Using a Purpose-Built Resuscitation Table-Study Protocol for a Randomized Controlled Trial**. *Frontiers in pediatrics* 2019, **7**:134.
58. ClinicalTrials.gov registry: **Aeration, Breathing, Clamping Study 3 (ABC3)**. https://clinicaltrialsgov/ct2/show/NCT03808051 *last accessed February 22, 2019*.
59. Ultee CA, van der Deure J, Swart J, Lasham C, van Baar AL: **Delayed cord clamping in preterm infants delivered at 34 36 weeks' gestation: a randomised controlled trial**. *Arch Dis Child Fetal Neonatal Ed* 2008, **93**(1):F20-23.
60. Australian New Zealand Clinical Trials Registry: **Provision of breathing support during delayed cord clamping in preterm infants.** https://wwwanzctrorgau/Trial/Registration/TrialReviewaspx?id=369228*, last accessed November 06, 2019*.
61. Malik AU, Shahnawaz K, Riaz A: **Comparison between the Efficacy of Early and Delayed Umbilical Cord Clamping in Preterm Infants**. *Pak J Med Health Sci* 2013, **7**(4):992-995.

For peer review only - http://bmjopen.bmj.com/site/about/guidelines.xhtml

62. ClinicalTrials.gov registry: **Deferred Cord Clamping Compared to Umbilical Cord Milking in Preterm Infants**. *https://clinicaltrialsgov/ct2/show/NCT02996799 last accessed February 22, 2019*.

63. ClinicalTrials.gov registry: **The Hematologic Impact of Umbilical Cord Milking Versus Deferred Cord Clamping in Premature Neonates**. *https://clinicaltrialsgov/ct2/show/NCT03147846 last accessed February 22, 2019*.

64. Hofmeyr GJ, Bolton KD, Bowen DC, Govan JJ: **Periventricular/intraventricular haemorrhage and umbilical cord clamping. Findings and hypothesis.** *S Afr Med J* 1988, **73**(2):104-106.

65. Hofmeyr GJ, Gobetz L, Bex PJM, Van Der Griendt M, Nikodem C, Skapinker R, Delahunt T: **Periventricular/intraventricular hemorrhage following early and delayed umbilical cord clamping. A randomized controlled trial.** . *Online J Curr Clin Trials* 1993(110).

66. Tiemersma S, Heistein J, Ruijne R, Lopez G, van Lobenstein J, van Rheenen P: **Delayed cord clamping in South African neonates with expected low birthweight: a randomised controlled trial**. *Tropical Medicine & International Health* 2015, **20**(2):177-183.

67. ISRCTN registry website: **DElayed COrd CLAmping versus early cord clamping in preterm infants born between 24 and 34 weeks**. *http://wwwisrctncom/ISRCTN66018314 last accessed August 27, 2018*.

68. Clinical Trials website: **Umbilical Cord Milking vs Delayed Cord Clamping in Preterm Infants Born by Cesarean Section**. *https://clinicaltrialsgov/ct2/show/NCT02187510 last accessed August 30, 2018*.

69. Lago Leal V, Pamplona Bueno L, Cabanillas Vilaplana L, Nicolas Montero E, Martin Blanco M, Fernandez Romero C, El Bakkali S, Pradillo Aramendi T, Sobrino Lorenzano L, Castellano Esparza P *et al*: **Effect of Milking Maneuver in Preterm Infants: A Randomized Controlled Trial**. *Fetal diagnosis and therapy* 2019, **45**(1):57-61.

70. Clinical Trials website: **Timing of Umbilical Cord Occlusion in Premature Babies(<33 w). Delayed vs Early. (CODE-P)**. *https://clinicaltrialsgov/ct2/show/NCT02187874 last accessed August 27, 2018*.

71. Baenziger O, Stolkin F, Keel M, von Siebenthal K, Fauchere JC, Das Kundu S, Dietz V, Bucher HU, Wolf M: **The influence of the timing of cord clamping on postnatal cerebral oxygenation in preterm neonates: a randomized, controlled trial**. *Pediatrics* 2007, **119**(3):455-459.

72. ISRCTN registry website: **Can squeezing blood from the umbilical cord into the baby reduce death and illness in premature babies? http://www.isrctn.com/ISRCTN17228884 last accessed February 26, 2019**.

73. Thai Clinical Trials Registry website: **Effect of delayed cord clamping versus cord milking in infants born at < 34 weeks gestation: a randomized controlled trial**. *http://wwwclinicaltrialsinth/indexphp?tp=regtrials&menu=trialsearch&smenu=fulltext&task=search&task2=view1&id=1277 last accessed August 31, 2018*.

74. Thai Clinical Trials Registry website: **Delayed cord clamping reduced anemic outcome in preterm neonate, http://www.clinicaltrials.in.th/index.php?tp=regtrials&menu=trialsearch&smenu=fulltext&task=search&task2=view1&id=3833 last accessed Feb 25, 2019.**

75. Mungkornkaew S, Siwadune T: **The Difference of Hematocrit in Term and Preterm Vaginal Births in Different Timing of Delayed Cord Clamping**. *Thai Journal of Obstetrics and Gynaecology* 2015:223-230.

76. Thai Clinical Trials Registry website: **Effects of delayed versus early cord clamping in late preterm infants: a randomized controlled trial**. *http://wwwclinicaltrialsinth/indexphp?tp=regtrials&menu=trialsearch&smenu=fulltext&task=search&task2=view1&id=1279 last accessed August 29, 2018*.

iCOMP

77. Thai Clinical Trials Registry website: **Comparison of Three Types of Placental transfusion in Preterm Infants: A Randomized Controlled Trial**. *http://wwwclinicaltrialsinth/indexphp?tp=regtrials&menu=trialsearch&smenu=fulltext&task=search&task2=view1&id=4427 last accessed November 06, 2019*.
78. Thai Clinical Trials Registry website: **A Randomized Controlled Trial of Immediate versus Delayed Umbilical Cord Clamping in Preterm Infants of Multiple Births**. *http://wwwclinicaltrialsinth/indexphp?tp=regtrials&menu=trialsearch&smenu=fulltext&task=search&task2=view1&id=2189 last accessed August 29, 2018*.
79. Salae R, Tanprasertkul C, Somprasit C, Bhamarapravatana K, Suwannarurk K: **Efficacy of delayed versus immediate cord clamping in late preterm newborns following normal labor: a randomized control trial**. *J Med Assoc Thai* 2016, **99**(4):S159-S165.
80. Thai Clinical Trials Registry website: **The Effect of One-Time Umbilical Cord Milking and Early Cord Clamping in Preterm Infants: A Randomized Controlled Trial (One-Time Umbilical Cord Milking)**. *http://clinicaltrialsinth/indexphp?tp=regtrials&menu=trialsearch&smenu=fulltext&task=search&task2=view1&id=2347 last accessed August 31, 2018*.
81. Alan S, Arsan S, Okulu E, Akin IM, Kilic A, Taskin S, Cetinkaya E, Erdeve O, Atasay B: **Effects of Umbilical Cord Milking on the Need for Packed Red Blood Cell Transfusions and Early Neonatal Hemodynamic Adaptation in Preterm Infants Born ≤1500 g: A Prospective, Randomized, Controlled Trial**. *Journal of Pediatric Hematology/Oncology* 2014, **36**(8):e493-e498.
82. Gokmen Z, Ozkiraz S, Tarcan A, Kozanoglu I, Ozcimen Emel E, Ozbek N: **Effects of delayed umbilical cord clamping on peripheral blood hematopoietic stem cells in premature neonates**. *Journal of Perinatal Medicine* 2011, **39**:323–329.
83. Kilicdag H, Gulcan H, Hanta D, Torer B, Gokmen Z, Ozdemir SI, Antmen BA: **Is umbilical cord milking always an advantage?** *The Journal of Maternal-Fetal & Neonatal Medicine* 2016, **29**(4):615-618.
84. Silahli M, Duman E, Gokmen Z, Toprak E, Gokdemir M, Ecevit A: **The relationship between placental transfusion, and thymic size and neonatal morbidities in premature infants - A Randomized Control Trial**. *JPMA The Journal of the Pakistan Medical Association* 2018, **68**(11):1560-1565.
85. Aladangady N, McHugh S, Aitchison TC, Wardrop CA, Holland BM: **Infants' blood volume in a controlled trial of placental transfusion at preterm delivery**. *Pediatrics* 2006, **117**(1):93-98.
86. Duley L, Dorling J, Pushpa-Rajah A, Oddie SJ, Yoxall CW, Schoonakker B, Bradshaw L, Mitchell EJ, Fawke JA: **Randomised trial of cord clamping and initial stabilisation at very preterm birth**. *Archives of Disease in Childhood - Fetal and Neonatal Edition* 2018, **103**(1):F6-F14.
87. Kinmond S, Aitchison TC, Holland BM, Jones JG, Turner TL, Wardrop CA: **Umbilical cord clamping and preterm infants: a randomised trial**. *BMJ (Clinical research ed)* 1993, **306**(6871):172-175.
88. Medina IMF: **Late clamping of the umbilical cord in premature neonates: The real haemodynamic benefits**. *Enfermeria Clinica* 2014, **24**(5):305-308.
89. Rabe H, Jewison A, Alvarez RF, Crook D, Stilton D, Bradley R, Holden D: **Milking compared with delayed cord clamping to increase placental transfusion in preterm neonates: a randomized controlled trial**. *Obstetrics & Gynecology* 2011, **117**(2):205-211.
90. Backes CH, Huang H, Iams JD, Bauer JA, Giannone PJ: **Timing of umbilical cord clamping among infants born at 22 through 27 weeks' gestation**. *J Perinatol* 2016, **36**(1):35-40.

 For peer review only - http://bmjopen.bmj.com/site/about/guidelines.xhtml

iCOMP

91. Clinical Trials website: **Effects of Delayed Cord Clamp and/or Indomethacin on Preterm Infant Brain Injury**. *https://clinicaltrialsgov/ct2/show/NCT02221219 last accessed August 27, 2018.*
92. ClinicalTrials.gov registry: **Relationship Between Delayed Cord Clamping at Birth and Neonatal Bilirubin Levels in Parturients With a Prior Child Requiring Therapy for Neonatal Jaundice, https://clinicaltrials.gov/ct2/show/NCT03741803 last accessed February 25th, 2019**.
93. Clinical Trials website: **Milking the Umbilical Cord Versus Immediate Clamping in Pre-term Infants < 33 Weeks**. *https://clinicaltrialsgov/ct2/show/NCT01819532 last accessed August 30, 2018.*
94. March MI, Hacker MR, Parson AW, Modest AM, de Veciana M: **The effects of umbilical cord milking in extremely preterm infants: A randomized controlled trial**. *J Perinatol* 2013, **33**(10):763-767.
95. ClinicalTrials.gov registry: **Delayed Umbilical Cord Clamping Versus Cord Milking in Preterm Neonate, https://clinicaltrials.gov/ct2/show/NCT01393834 last accessed 25th February, 2019.**
96. Clinical Trials website: **A Randomized Controlled Trial of Immediate Versus Delayed Cord Clamping in the Preterm Neonate**. *https://clinicaltrialsgov/ct2/show/NCT00579839 last accessed August 27, 2018.*
97. Ibrahim HM, Krouskop RW, Lewis DF, Dhanireddy R: **Placental transfusion: umbilical cord clamping and preterm infants**. *Journal of Perinatology* 2000, **20**(6):351.
98. Josephsen J, Vlastos E, Potter S, Al-Hosni M: **Milking the umbilical cord in extreme preterm infants**. *Am J Obstet Gynecol* 2014, **10**(1 Suppl):S403-404.
99. Katheria AC, Leone TA, Woelkers D, Garey DM, Rich W, Finer NN: **The effects of umbilical cord milking on hemodynamics and neonatal outcomes in premature neonates**. *J Pediatrics* 2014, **164**(5):1045-1050.
100. Katheria AC, Truong G, Cousins L, Oshiro B, Finer NN: **Umbilical cord milking versus delayed cord clamping in preterm infants**. *Pediatrics* 2015, **136**(1):61-69.
101. Katheria A, Poeltler D, Durham J, Steen J, Rich W, Arnell K, Maldonado M, Cousins L, Finer N: **Neonatal Resuscitation with an Intact Cord: A Randomized Clinical Trial**. *Journal of Pediatrics* 2016, **178**:75-80.e73.
102. ClinicalTrials.gov registry: **Premature Infants Receiving Milking or Delayed Cord Clamping: PREMOD2. Available at https://clinicaltrials.gov/ct2/show/NCT03019367. Accessed August 23, 2019.**
103. ClinicalTrials.gov registry: **Umbilical Cord Milking in Non-Vigorous Infants (MINVI). Available at https://clinicaltrials.gov/ct2/show/NCT03631940. Accessed August 23, 2019.**
104. Clinical Trials website: **VentFirst: A Multicenter RCT of Assisted Ventilation During Delayed Cord Clamping for Extremely Preterm Infants**. *https://clinicaltrialsgov/ct2/show/NCT02742454 last accessed August 27, 2018.*
105. Krueger MS, Eyal FG, Peevy KJ, Hamm CR, Whitehurst RM, Lewis DF: **Delayed cord clamping with and without cord stripping: A prospective randomized trial of preterm neonates**. *Am J Obstet Gynecol* 2015, **212**(3):394.e391-395.
106. Clinical Trials website: **Optimal Timing of Cord Clamping in Preterm Pregnancy Following Vaginal or Cesarean Delivery (CordClamp)**. *https://clinicaltrialsgov/ct2/show/NCT01766908 last accessed August 27, 2018.*
107. Mercer JS, McGrath MM, Hensman A, Silver H, Oh W: **Immediate and delayed cord clamping in infants born between 24 and 32 weeks: a pilot randomized controlled trial**. *Journal of perinatology* 2003, **23**(6):466.

For peer review only - http://bmjopen.bmj.com/site/about/guidelines.xhtml

108. Mercer JS, Vohr BR, McGrath MM, Padbury JF, Wallach M, Oh W: **Delayed cord clamping in very preterm infants reduces the incidence of intraventricular hemorrhage and late-onset sepsis: a randomized, controlled trial**. *Pediatrics* 2006, **117**(4):1235-1242.
109. Mercer JS, Erickson-Owens DA, Vohr BR, Tucker RJ, Parker AB, Oh W, Padbury JF: **Effects of placental transfusion on neonatal and 18 month outcomes in preterm infants: a randomized controlled trial**. *J Pediatrics* 2016, **168**:50-55.
110. Oh W, Fanaroff AA, Carlo WA, Donovan EF, McDonald SA, Poole WK, Eunice Kennedy Shriver National Institute of Child Health and Human Development Neonatal Research Network: **Effects of delayed cord clamping in very-low-birth-weight infants**. *J Perinatol* 2011, **31**:S68-S71.
111. Clinical Trials website: **Delayed Cord Clamping in Preterm Neonates (DCC)**. *https://clinicaltrialsgov/ct2/show/NCT02478684 last accessed August 29, 2018*.
112. Clinical Trials website: **Delayed Clamping and Milking the Umbilical Cord in Preterm Infants**. *https://clinicaltrialsgov/ct2/show/NCT02092103 last accessed August 31, 2018*.
113. Strauss RG, Mock DM, Johnson KJ, Cress GA, Burmeister LF, Zimmerman MB, Bell EF, Rijhsinghani A: **A randomized clinical trial comparing immediate versus delayed clamping of the umbilical cord in preterm infants: short-term clinical and laboratory endpoints**. *Transfusion* 2008, **48**(4):658-665.
114. ClinicalTrials.gov registry: **Delayed Cord Clamping in Very Low Birth Weight Infants (DCC). Available at https://clinicaltrials.gov/ct2/show/NCT02337088. Accessed August 23, 2019.**
115. Pongmee P, Nuntnarumit P: **Effects of umbilical cord milking on initial hematocrit and the need for blood transfusion in preterm infants. In: Pediatric Academic Societies Annual Meeting; 2010 May 1-4; Vancouver, Canada. 2010**.

For peer review only

Supplementary File 2: PRISMA-P checklist

**Section and topic**	**Item no.**	**Checklist item**	**Page number**
Title	1a	Identify the report as a protocol of a systematic review	p.1
	1b	If protocol is for an update of a previous review, identify as such	Not applicable
Registration	2	If registered, provide the name of the registry and registration number.	p.2
Authors	3a	Provide name, institutional affiliation, email address of all protocol authors, provide physical mailing address of corresponding author.	p.1
	3b	Describe contributions of protocol authors and identity the guarantor of the review.	p.16
Amendments	4	If the protocol represents an amendment of a previously completed or published protocol, identify as such and list changes; otherwise, state plan for documenting important protocol amendments.	p.11, p.12
Support	5a	Indicate sources of financial or other support for the review.	p.16
	5b	Provide name for review funder and/or sponsor.	p.16
	5c	Describe roles of funder(s), sponsor(s), and/or institution(s), if any, in developing the protocol.	p.16
Rationale	6	Describe the rational for the review in the context of what is already known.	p.4-5
Objectives	7	Provide an explicit statement of the question(s) the review will address with reference to participants, interventions, comparators and outcomes (PICO)	p.6
Eligibility criteria	8	Specify the study characteristics (such as PICO, study design, setting time frame) and report characteristics (such as years considered, language, publication status) to be used as criteria for eligibility for the review.	p.6-8
Information sources	9	Describe all intended information sources (such as electronic databases, contact with study authors, trial registers or other grey literature sources) with planned dates of coverage.	p.8-9
Search strategy	10	Present draft of search strategy to be used for at least one electronic database, including planned limits, such that it could be repeated.	Supplementary File 3

For peer review only

Study records	11a	Describe the mechanism(s) that will be used to manage records and data throughout the review.	p.8 ff.
	11b	State the process that will be used for selecting studies (such as two independent reviewers) through each phase of the review (that is, screening, eligibility and inclusion in meta-analysis).	p.8
	11c	Describe planned method of extracting data from reports (such as piloting forms, done independently, in duplicate), any processes for obtaining and confirming data from investigators.	p.8 ff.
Data items	12	List and define all variables for which data will be sought (such as PICO items, funding sources), and pre-planned data assumptions and simplifications.	p.9 ff.
Outcomes and prioritization	13	List and define all outcomes for which data will be sought, including prioritization of main and additional outcomes, with rationale.	p.9 ff.
Risk of bias in individual studies	14	Describe anticipated methods for assessing risk of bias of individual studies, including whether this will be done at the outcome or study level, or both; state how this information will be used in data synthesis.	p.9
Data synthesis	15a	Describe criteria under which study data will be quantitatively synthesised.	p.12, p.13
	15b	If data are appropriate for quantitative synthesis, describe planned summary measures, methods of handling data and methods of combining data from studies, including any planned exploration of consistency (such as I^2, Kendall's τ).	p.12, p.13
	15c	Describe any proposed additional analyses (such as sensitivity or subgroup analyses, meta-regression).	p.14
	15d	If quantitative synthesis is not appropriate, describe the type of summary planned.	n.a.
Meta-bias(es)	16	Specify any planned assessment of meta-bias(es) (such as publication bias across studies, selective reporting within studies).	p.14
Confidence in cumulative evidence	17	Describe how the strength of the body of evidence will be assessed (such as GRADE).	p.9

For peer review only

iCOMP search strategy

1. We used search results from a prospective meta-analysis we had previously planned to conduct on cord clamping in preterm infants. Regular searches were conducted from 2010 to 2017.
2. We used search results up to November 2018 from a recently updated Cochrane review on cord clamping in preterm infants, on which some of us are authors (Rabe H, Gyte GML, Díaz-Rossello JL, Duley L. Effect of timing of umbilical cord clamping and other strategies to influence placental transfusion at preterm birth on maternal and infant outcomes. Cochrane Database of Systematic Reviews 2019, Issue 9. Art. No.: CD003248. DOI: 10.1002/14651858.CD003248.pub4.)
3. We conducted new independent searches for the period from November 2018 onwards.

Details of all searches are elucidated below.

1. Search methods – for previously planned PMA (up to September 2017)

We regularly searched the World Health Organisation (WHO) International Clinical Trials Registry Platform (ICTRP) from the period January 2010 to September 2017. In total, fourteen searches were conducted over this period, using a combination of the search terms shown below.

1.1 ICTRP

1. placental transfusion
2. cord clamp*
3. umbilical cord clamp*
4. cord milking
5. milking
6. umbilical cord
7. preterm
8. pre-term
9. prematur*

2. Search methods - Cochrane review update (8th November 2018)

The following sources were searched: Cochrane Pregnancy and Childbirth's Trials Register, ClinicalTrials.gov and WHO ICTRP. Further details of each are provided below.

2.1 Pregnancy and Childbirth's Trials Register

- Searched 8 November 2018 by the Cochrane Pregnancy and Childbirth Information Specialist.
- For detailed information about the registry and search strategies, please go to https://pregnancy.cochrane.org/pregnancy-and-childbirth-groups-trials-register.

2.2 ICTRP

cord AND clamp
cord and clamping
cord AND milking
cord AND stripping

2.3 ClinicalTrials.gov

Advanced search

Interventional studies | cord clamping
Interventional studies | cord milking
Interventional studies | cord stripping

For peer review only

3. Search methods (13th February 2019)

We searched Ovid MEDLINE, Embase, EBM Reviews - Cochrane Central Register of Controlled Trials, WHO ICTRP and ClinicalTrials.gov using the search strings below.

3.1 Ovid MEDLINE(R)

1. umbilical-cord.mp. or exp umbilical cord/
2. (Clamp$ OR Milk$).af.
3. (Placenta$ adj2 transfus$).af
4. 2 or 3
5. exp Infant, Premature/ or preterm*.mp.
6. prematur*.mp.
7. exp Infant, Low Birth Weight/ or exp Infant, Very Low Birth Weight
8. exp Infant, Extremely Low Birth Weight
9. 5 or 6 or 7 or 8
10. 1 and 4 and 9
11. limit 10 to (humans and clinical trial, all)
12. limit 11 to ed=20181001-20190213

3.2 Embase

1. umbilical-cord.mp. or exp umbilical cord/
2. (Clamp$ OR Milk$).af.
3. (Placenta$ adj2 transfus$).af
4. 2 or 3
5. exp Infant, Premature/ or preterm*.mp.
6. prematur*.mp.
7. exp Infant, Low Birth Weight/ or exp Infant, Very Low Birth Weight
8. exp Infant, Extremely Low Birth Weight
9. 5 or 6 or 7 or 8
10. 1 and 4 and 9
11. limit 10 to (human and randomized controlled trial)
12. limit 11 to yr="2018 -Current"

3.3 EBM Reviews - Cochrane Central Register of Controlled Trials

1. umbilical-cord.mp. or exp umbilical cord/
2. (Clamp$ OR Milk$).af.
3. (Placenta$ adj2 transfus$).af
4. 2 or 3
5. exp Infant, Premature/ or preterm*.mp.
6. prematur*.mp.
7. exp Infant, Low Birth Weight/ or exp Infant, Very Low Birth Weight
8. exp Infant, Extremely Low Birth Weight
9. 5 or 6 or 7 or 8
10. 1 and 4 and 9
11. limit 10 to yr="2018 -Current"

For peer review only

3.4 WHO ICTRP

Search string
Basic search
1. placental transfusion<br>(limit date of registration from 1/11/2018 onwards)
2. cord clamp<br>(limit date of registration from 1/11/2018 onwards)
3. cord clamping<br>(limit date of registration from 1/11/2018 onwards)
4. milking<br>(limit date of registration from 1/11/2018 onwards)
Advanced search
5. Title: umbilical cord<br>Condition: preterm OR premature<br>Recruitment Status: All<br>(limit date of registration from 1/11/2018 onwards)
6. Condition: preterm OR premature<br>Intervention: "umbilical cord"<br>Recruitment Status: All<br>(limit date of registration from 1/11/2018 onwards)

3.5 Clinicaltrials.gov

Search string
Basic search
1. Other terms: "placental transfusion"<br>First posted from 11/01/2018 to 02/13/2019 (MM/DD/YYYY)
2. Other terms: "cord clamp"<br>First posted from 11/01/2018 to 02/13/2019 (MM/DD/YYYY)
3. Other terms: "cord clamping"<br>First posted from 11/01/2018 to 02/13/2019 (MM/DD/YYYY)
4. Other terms: milking<br>First posted from 11/01/2018 to 02/13/2019 (MM/DD/YYYY)
5. Condition or disease: Preterm Birth<br>Other terms: "umbilical cord"<br>First posted from 11/01/2018 to 02/13/2019 (MM/DD/YYYY)

For peer review only
